# Global distributions of age- and sex-related arterial stiffness: systematic review and meta-analysis of 167 studies with 509,743 participants

**DOI:** 10.1016/j.ebiom.2023.104619

**Published:** 2023-05-23

**Authors:** Yao Lu, Sophia J. Kiechl, Jie Wang, Qingbo Xu, Stefan Kiechl, Raimund Pechlaner, Global Pulse Wave Velocity Study Group, David Aguilar, Khamis M. Al-Hashmi, Rafael O. Alvim, Ibrahim S. Al-Zakwani, Christina Antza, Arrigo F.G. Cicero, Maja Avramovska, Petar Avramovski, Hyun Jae Baek, Magnus Bäck, Kent Bailey, Marcelo P. Baldo, Rosângela F.L. Batista, Athanasios Benetos, Emelia J. Benjamin, Daniel Bia, Claudio Borghi, Shani Botha-Le Roux, Yolandi Breet, David Burgner, Viviane C. Cardoso, Marina Cecelja, Indre Ceponiene, Chen-Huan Chen, Michael Cheung, Hao-min Cheng, Jaegeol Cho, Phil Chowienczyk, Eduardo B. Coelho, Orsolya Cseprekal, Amilcar BT Da Silva, Frédéric Dallaire, Roberto De Sá Cunha, Alejandro Diaz, Albano V.L. Ferreira, Jean Ferrières, Yoshihiko Furuta, Manuel A. Gómez-Marcos, Leticia Gómez-Sánchez, Julian Halcox, Craig Hanis, Karl-Heinz Herzig, Edgar Jaeggi, Maryam Kavousi, Ursula Kiechl-Kohlendorfer, Hack-Lyoung Kim, Mi-Kyung Kim, Yu-Mi Kim, Eva Kis, Michael Knoflach, Vasilios Kotsis, Teruhide Koyama, Michaela Kozakova, Ruan Kruger, Iftikhar J. Kullo, Sun-Seog Kweon, Irene Lambrinoudaki, Chang Liu, Markus Loeffler, Jeongok G. Logan, Jane Maddock, Pedro Magalhães, João Maldonado, Francesco U.S. Mattace-Raso, Alex Messner, Michelle L. Meyer, Jie Mi, José Geraldo Mill, Gary F. Mitchell, Jian-Jun Mu, Iram F. Muhammad, Johannes Nairz, Atsushi Nakagomi, Mieko Nakamura, Peter M. Nilson, Toshiharu Ninomiya, Carlo Palombo, Alexandre C. Pereira, Telmo Pereira, Daniel P. Capingana, Anna K. Poon, Nicole Probst-Hensch, Arshed A. Quyyumi, George S. Reusz, Moo-Yong Rhee, Cecilia C.C. Ribeiro, Ernst Rietzschel, Paulo R.H. Rocha, Enrique Rodilla, Marta Rojek, Jean-Bernard Ruidavets, Joost H.W. Rutten, Yasuaki Saijo, Paolo Salvi, Arno Schmidt-Trucksäss, Markus Scholz, Min-Ho Shin, Patrick Segers, Kimon Stamatelopoulos, Irina D. Strazhesko, Minoru Sugiura, Olga N. Tkacheva, Hirofumi Tomiyama, Elaine M. Urbina, Inge C.L. van den Munckhof, Ramachandran S. Vasan, Melissa A. Wake, Goya Wannamethee, Andrew Wong, Akira Yamashina, Yinkun Yan, Divanei Zaniqueli, Fang Zhu, Yanina Zócalo

**Affiliations:** a Clinical Research Center, The Third Xiangya Hospital, Central South University, Changsha, China; b School of Life Course Sciences, King's College London, London, United Kingdom; c Department of Neurology, Medical University of Innsbruck, Innsbruck, Austria; d Department of Neurology, Hochzirl Hospital, Zirl, Austria; e Research Centre on Vascular Ageing and Stroke, Innsbruck, Austria; f Centre for Clinical Pharmacology, William Harvey Research Institute, Barts and the London School of Medicine and Dentistry, Queen Mary University of London, United Kingdom

**Keywords:** Pulse wave velocity, Arterial stiffness, Hypertensive end-organ damage, All-cause mortality, Cardiovascular disease, Risk factors, Prevention, Reference values

## Abstract

**Background:**

Arterial stiffening is central to the vascular ageing process and a powerful predictor and cause of diverse vascular pathologies and mortality. We investigated age and sex trajectories, regional differences, and global reference values of arterial stiffness as assessed by pulse wave velocity (PWV).

**Methods:**

Measurements of brachial-ankle or carotid-femoral PWV (baPWV or cfPWV) in generally healthy participants published in three electronic databases between database inception and August 24th, 2020 were included, either as individual participant-level or summary data received from collaborators (n = 248,196) or by extraction from published reports (n = 274,629). Quality was appraised using the Joanna Briggs Instrument. Variation in PWV was estimated using mixed-effects meta-regression and Generalized Additive Models for Location, Scale, and Shape.

**Findings:**

The search yielded 8920 studies, and 167 studies with 509,743 participants from 34 countries were included. PWV depended on age, sex, and country. Global age-standardised means were 12.5 m/s (95% confidence interval: 12.1–12.8 m/s) for baPWV and 7.45 m/s (95% CI: 7.11–7.79 m/s) for cfPWV. Males had higher global levels than females of 0.77 m/s for baPWV (95% CI: 0.75–0.78 m/s) and 0.35 m/s for cfPWV (95% CI: 0.33–0.37 m/s), but sex differences in baPWV diminished with advancing age. Compared to Europe, baPWV was substantially higher in the Asian region (+1.83 m/s, P = 0.0014), whereas cfPWV was higher in the African region (+0.41 m/s, P < 0.0001) and differed more by country (highest in Poland, Russia, Iceland, France, and China; lowest in Spain, Belgium, Canada, Finland, and Argentina). High vs. other country income was associated with lower baPWV (−0.55 m/s, P = 0.048) and cfPWV (−0.41 m/s, P < 0.0001).

**Interpretation:**

China and other Asian countries featured high PWV, which by known associations with central blood pressure and pulse pressure may partly explain higher Asian risk for intracerebral haemorrhage and small vessel stroke. Reference values provided may facilitate use of PWV as a marker of vascular ageing, for prediction of vascular risk and death, and for designing future therapeutic interventions.

**Funding:**

This study was supported by the excellence initiative VASCage funded by the 10.13039/501100004955Austrian Research Promotion Agency, by the 10.13039/501100001809National Science Foundation of China, and the Science and Technology Planning Project of Hunan Province. Detailed funding information is provided as part of the Acknowledgments after the main text.

**Research in context**

**Evidence before this study**

Several studies investigated geographical variation in arterial stiffness as measured by pulse wave velocity (PWV) in healthy participants, but no survey on a global scale is available. We searched MEDLINE (via PubMed), Web of Science, and Embase from database inception to August 24, 2020, using search terms pertaining to arterial stiffness and pulse wave velocity without language restrictions. We additionally scanned reference lists of relevant articles and reviews and included additional unpublished datasets provided by the Study Group. We contacted researchers for individual participant or summary data of brachial-ankle or carotid-femoral PWV (baPWV or cfPWV). Study quality as assessed by the Joanna Briggs instrument was high for 83% of 167 included studies. Global age-standardised baPWV was 12.5 m/s and cfPWV 7.45 m/s.

**Added value of this study**

We provide reference PWV values, details necessary to use PWV in clinical routine, and comparisons between geographical regions, countries, measurement methods and by study quality.

**Implications of all the available evidence**

Arterial stiffness as measured by PWV is a surrogate of vascular ageing and a strong predictor of cardiovascular disease and death independent of traditional risk factors. High PWV in China and other Asian countries may contribute to excess incidence of ischemic and haemorrhagic stroke. Arterial stiffness is an appealing target for pharmacological and lifestyle intervention, however, large-scale controlled trials are needed to guide clinicians.

## Introduction

Cardiovascular disease (CVD) is the leading cause of mortality globally and a major contributor to reduced quality of life.^1^ Effective primary prevention of CVD requires early identification of individuals at high risk and early intervention. Arterial stiffening represents a sensitive marker of vascular pathology and reflects atherosclerosis, vascular calcification, inflammation, and genuine vascular ageing featured by smooth muscle cell senescence and fragmentation and degeneration of elastic fibres (Central Illustration).^2^ Arterial stiffness precedes increases in blood pressure^3^ and is a strong predictor of CVD and death.^4^ Higher aortic stiffness is associated with higher risk of heart failure,^5^ atrial fibrillation,^6^ aortic aneurysms, impaired coronary artery perfusion, and stroke.^2^^,^^7^ Arterial stiffness augments central blood pressure, which damages the microcirculation of organs with low vascular resistance, in particular kidney and brain, entailing cerebral microbleeds, haemorrhagic stroke, lacunar stroke, and cognitive impairment.^2^ Aortic stiffness has also been associated with entorhinal tau deposition,^8^ a hallmark of Alzheimer's disease.

**Central Illustration:**
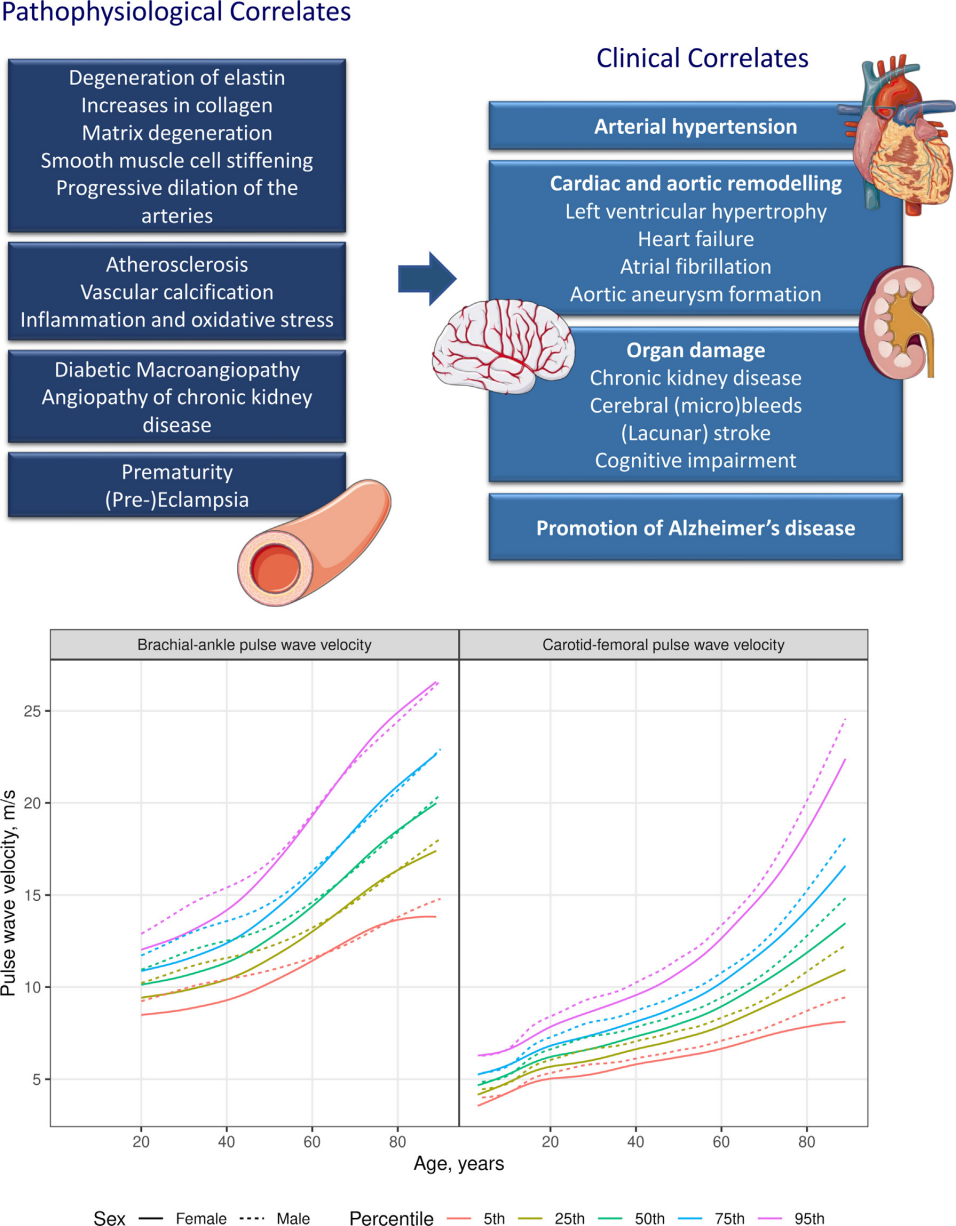
Pathophysiological and clinical correlates of arterial stiffness and trajectories of pulse wave velocity by age and sex. Arterial stiffness represents a sensitive marker of vascular ageing reflecting diverse vascular pathologies (top left). Arterial stiffening increases central blood pressure and pulse pressure, predisposing to cardiac disease by increasing afterload and decreasing coronary perfusion and damaging the microcirculation of the low vascular resistance organs kidney and brain (top right). Pulse wave velocity (PWV) is a validated non-invasive measure of arterial stiffness and powerfully predicts both CVD and all-cause mortality. Here we provide age- and sex-specific regional and global (bottom part of figure) distributions and reference values for brachial-ankle and carotid-femoral PWV to facilitate routine clinical use of PWV. This figure includes content from Servier Medical Art by Servier, licensed under a Creative Commons Attribution 3.0 unported license.

Pulse wave velocity (PWV) is a validated, non-invasive measure of arterial stiffness^2^^,^^9^ that represents the speed of the arterial pressure wave propagation along an artery and increases in parallel with stiffness of the vessel wall. Aortic PWV is a powerful predictor of CVD independent of traditional risk factors^10^^,^^11^ and of all-cause mortality with a 15% increase for each one m/s increase in PWV.^4^ Carotid-femoral PWV (cfPWV) and brachial-ankle PWV (baPWV) are the two most commonly used types of PWV. While both reflect stiffness of central conduit arteries, baPWV is also affected by stiffness of peripheral arteries.^9^

Detailed assessment of age- and sex-related variation and reference values of PWV by world region and country are needed to promote PWV measurement in clinical routine. We previously reported baPWV trajectories across the whole lifespan in 80,415 Chinese community dwellers.^12^ Here, we performed a comprehensive global meta-analysis of PWV trajectories of unprecedented size (n = 509,743). We included all available studies that measured baPWV, cfPWV, or both in community-dwelling or generally healthy individuals to systematically assess global and regional distributions and age trajectories of PWV and create a compendium of global PWV distributions.

## Methods

For this systematic review and meta-analysis, we estimated distributions and age trajectories of baPWV and cfPWV based on data from generally healthy individuals examined worldwide, including individual-participant data and summary data provided by collaborators (n = 248,196) as well as summary data extracted from published reports (n = 274,629), in this order of preference. All researchers identified by the systematic literature search were contacted and invited to join the Global PWV Study Group. Further studies or unpublished data suggested by collaborators and studies cited by studies identified via the systematic review were also considered if inclusion criteria were fulfilled.

### Data collection

The systematic review identified studies by searching the electronic databases of MEDLINE (via PubMed), Web of Science, and Embase from database inception to August 24, 2020, without language restrictions. The search strategies were composed of keywords relating to values of cfPWV or baPWV as detailed in the Appendix (p 8). Data on generally healthy humans mainly included data derived from community-based observational studies. Reviews, case reports, repeated studies, patient series, duplicate data, or studies with sample sizes below 100 were not considered. Included studies are shown in the Appendix (pp 9–35). Study quality was graded using the Joanna Briggs Instrument for Analytical Cross Sectional Studies as shown in the Appendix (pp 36–41). The systematic literature search, data extraction, and quality grading were performed by two researchers working independently, with disagreements resolved by consulting a third, senior researcher.

Covariables considered were age, sex, country and year of study execution, study size, and details of the PWV measurement method used. Individual-participant and summary data were allocated to groups defined by decade of age, sex, study, and study country. Data extracted from published reports were transformed if needed as detailed in the Appendix (p 3). Participants aged 90 years or older were included in a ≥90 age group.

World regions to which countries belonged were based on the World Health Organization world region classification with “Region of the Americas” split into the Northern and Southern American continent, and Western Pacific and South-East Asia region merged to form an Asia region due to only one study available for baPWV and for cfPWV each in the South-East Asia region. Only world regions with at least two studies available were considered. Country income was taken from World Bank Country and Lending Groups. The impact of measurement device was assessed, and measurement method was classified as oscillometry, tonometry, or ultrasound. Measurements derived from magnetic resonance imaging (n = 2 studies), aortic PWV, or pulse contour analysis (e.g., the UK Biobank Study) were not considered. PWV is calculated as distance over transit time and the classification of different procedures for deriving distance is shown in the Appendix (p 4). If different PWV types (baPWV or cfPWV) were reported in the same original publication, data were analysed as in separate individual studies. If year of study performance was given as a range, the midpoint of the range was used; if not available (n = 7 of 167 studies, 4.2%), year of publication was used instead.

### Statistical analysis

Primary outcome parameters were averages of PWV by age, sex, and geographical covariables, differences in PWV between defined subgroups, and 95th percentiles of PWV to be employed as reference values.

Global and country-wise age- and sex-conditional distributions of PWV and reference values were derived from individual-participant data (n = 178,073 data points and participants, 45 studies, 24 countries) using Generalized Additive Models for Location, Scale and Shape (GAMLSS).^13^ These flexible semi-parametric models represent a generalization of the LMS method and are preferred for the construction of reference curves.^14^ More detail is given in the Appendix (p 3).

Averages of PWV by age, sex, geographical and other covariables were derived from all available data (n = 509,743 participants, 13,082 of which had both baPWV and cfPWV measurements, 167 studies, 34 countries) using meta-regression of single means based on inverse variance weighted linear mixed effects models as implemented by the metafor R package.^15^ Random effects included random intercepts for individual studies and an autoregressive AR (1) covariance structure for decade of age. Fixed nonlinear age effects were modelled using restricted cubic splines with four evenly spaced knots. Effects were derived by linear combinations of model parameters and Wald-type tests, and likelihood ratio tests of nested models used to test for any difference among subgroups. Age-standardised PWV globally and within subgroups was derived by weighted pooling of model predictions using the age distribution of the World Health Organization World 2000–2025 Standard Million population. Sensitivity analyses included calculation of results after exclusion of unpublished studies.

P-values are 2-sided and an alpha level of 0.05 is used. Analysis was conducted using R version 4.2.0 (R Foundation for Statistical Computing, Vienna, Austria).

### Role of the funding sources

The funding sources of the study had no role in study design, data collection, data analysis, data interpretation, writing of the report, or the decision to submit the report for publication.

## Results

The systematic literature search yielded 16,817 results (8920 after exclusion of duplicate studies; Appendix p 5), which after applying inclusion criteria resulted in 114 eligible studies. Of the studies and additional datasets suggested by the Global PWV Study Group or referenced by included studies, a further 53 fulfilled inclusion criteria. Characteristics of included studies are shown in the Appendix (pp 9–35). The global meta-analysis of arterial stiffness included 509,743 participants of 167 studies conducted in 34 countries (Appendix p 5). Respective numbers for baPWV were 356,450 (78 studies, 7 countries) and for cfPWV 166,375 (93 studies, 32 countries).

Global age-standardised average baPWV was 12.5 m/s (95% confidence interval: 12.1–12.8 m/s) and cfPWV, 7.45 m/s (95% CI: 7.11–7.79 m/s), with higher levels in males than in females of 0.77 m/s (95% CI: 0.75–0.78 m/s) for baPWV and 0.35 m/s (95% CI: 0.33–0.37 m/s) for cfPWV.

**Fig. 1:**
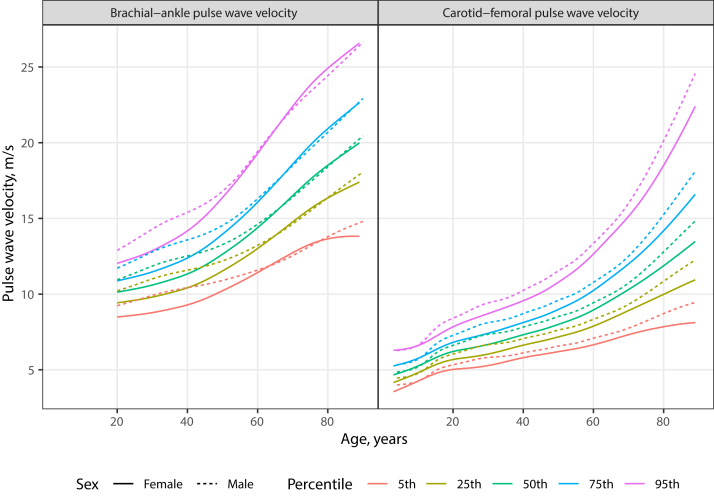
**Global age- and sex-dependent distributions of baPWV and cfPWV**. For both baPWV and cfPWV, levels and variability increased with age. Males showed a faster increase than females in baPWV after age 20, but sex differences vanished at higher ages. For cfPWV, males featured higher values beginning in adolescence, and the sex difference remained constant throughout the remaining lifespan. This analysis is based on n = 178,073 individual data points and participants from 45 studies conducted in 24 countries.

The global age- and sex-dependent distribution of PWV is shown in Fig. 1 and individual quantiles and reference values are given in the Appendix (pp 42–47). Estimates of cfPWV age trajectories based on the current cross-sectional data indicated an increase of cfPWV during adolescence, followed by a slower ascent until approximately age 60 years, and a steep ascent thereafter. For baPWV, a similar dynamic was apparent, but no individual participant data below age 20 years were available. Sex differences in cfPWV emerged only after adolescence, with higher mean cfPWV in males than females, which remained constant during the remaining lifespan. For baPWV, males had a markedly faster increase than females during young adulthood, but at higher ages this pattern reversed.

**Fig. 2:**
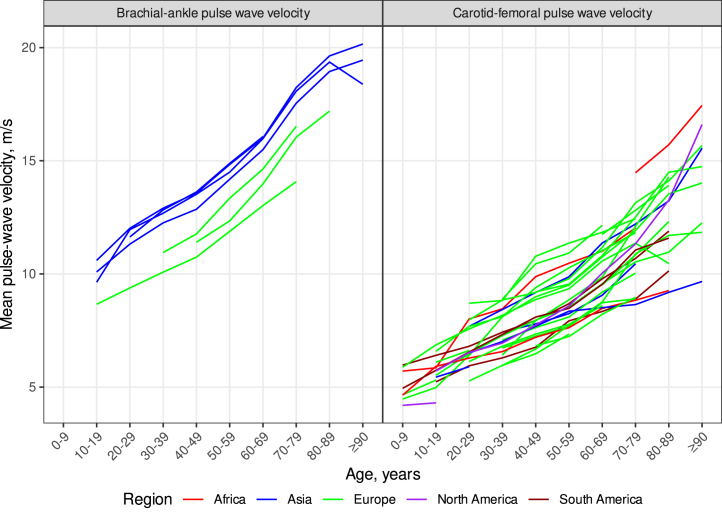
**Trajectories of PWV over the lifespan by country and world region**. Individual lines represent individual countries and line colours, world regions. Age trajectories of baPWV were relatively uniform within Asia and Europe individually whereas inter-country differences were more pronounced for cfPWV trajectories. This analysis is based on data from n = 509,743 participants of 167 studies conducted in 34 countries.

**Fig. 3:**
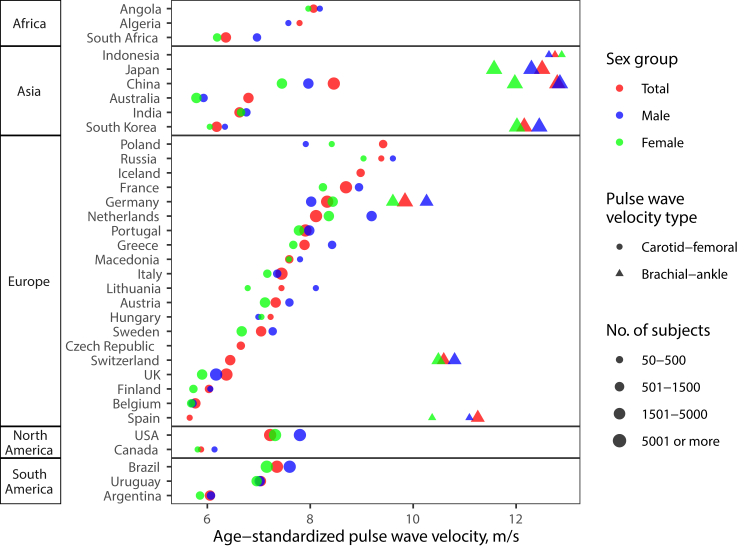
**Age-standardised PWV by country and world region**. Countries are ordered according to average PWV. baPWV was uniformly lower in European as compared to Asian countries. There was a wide range of cfPWV averages between countries within the same region. Poland, Russia, Iceland, France, and China featured the highest cfPWV. Estimates for the “Total” sex group include PWV summary data extracted from the literature without reporting of PWV by sex and may therefore differ from pooled estimates of included data in men and in women.

**Fig. 4:**
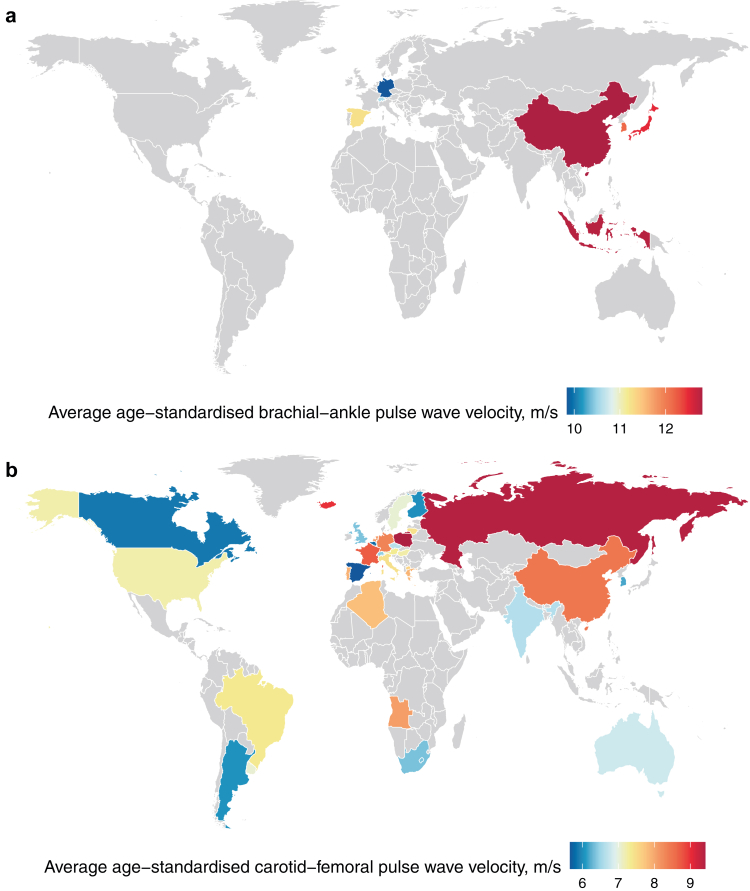
***a*:World map ofbaPWV by country.** For Indonesia, data from fewer than 1000 participants were available. ***b*: World map of cfPWV by country.** For the following countries, data from fewer than 1000 participants were available: Algeria, Angola, Canada, Hungary, Iceland, Lithuania, North Macedonia, Poland, Russia, and Spain.

**Fig. 5:**
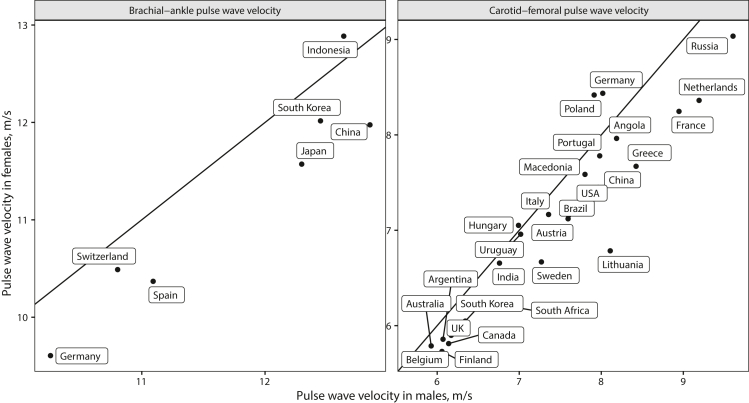
**Sexdifferences in PWV by country.** Age-standardized PWV values in males (x axis) vs. in females (y axis) are shown by country. Males featured higher baPWV and cfPWV in most countries and sex differences were more uniform between countries for baPWV than for cfPWV. Variability in PWV due to sex was less than variability due to country. Diagonal lines indicate identical PWV in males and females. Algeria is not shown because no data on cfPWV in Algerian females was available.

Investigating geographical differences, similar baPWV levels and distributions were found within Asian countries and within European countries (Fig. 2, Fig. 3) with age-standardised baPWV substantially higher in Asia vs. Europe (+1.83 m/s, P = 0.0014, Table 1). For cfPWV, age-standardised values were similar between world regions (Table 2) except for Africa (+0.41 m/s; P < 0.0001) but country-specific values were more variable (Fig. 2, Fig. 3) with cfPWV highest in Poland, Russia, Iceland, France, and China and lowest in Spain, Belgium, Canada, Finland, and Argentina (Fig. 4a and b). Distributions by country are displayed in the Appendix (pp 6–7). Of note, the United States of America had the steepest increase in cfPWV after age 60 (Appendix p 7). Country-specific sex differences in PWV are summarized in Fig. 5 and indicated higher PWV in males in all countries except Germany, Hungary, Indonesia, and Poland.

**Table 1:** Age-standardised baPWV by world region, country, and other covariables.

Variable	Category	Mean PWV (95% CI)	No. participants	No. studies	P	Diff. to ref. category (95% CI)
All data		12.5 (12.1–12.8)	356,450	78		
Country	China	12.8 (12.3–13.3)	227,799	29	0.029	0 (ref)
	Germany	9.84 (7.74–11.94)	8478	1		−2.96 (−5.09 to −0.83)
	Indonesia	12.8 (10.6–14.9)	132	1		−0.04 (−2.21 to 2.13)
	Japan	12.5 (12.0–13.0)	62,837	30		−0.29 (−0.85 to 0.27)
	South Korea	12.2 (11.5–12.8)	51,327	14		−0.64 (−1.33 to 0.05)
	Spain	11.3 (9.7–12.8)	2815	2		−1.54 (−3.09 to 0.00)
	Switzerland	10.6 (8.5–12.7)	3062	1		−2.21 (−4.35 to −0.06)
Country income	High Income	12.3 (11.9–12.7)	128,519	48	0.048	0 (ref)
	Other Income	12.8 (12.3–13.3)	227,931	30		0.55 (0.01–1.08)
Device	Sphygmocor	12.2 (10.0, 14.5)	11,014	1	0.12	0 (ref)
	Omron	12.6 (12.2, 12.9)	213,674	66		0.32 (−1.89, 2.54)
	Parks Doppler	10.8 (8.6, 13.0)	6719	1		−1.46 (−4.56, 1.64)
	Vasera	12.0 (11.0, 13.0)	6655	5		−0.24 (−2.65, 2.18)
	Vicorder	9.84 (7.67, 12.02)	8478	1		−2.40 (−5.49, 0.69)
	Other	12.5 (11.4, 13.6)	109,910	4		0.24 (−2.22, 2.71)
Measurement method	Tonometry	12.2 (9.9–14.6)	11,014	1	0.35	0 (ref)
	Oscillometry	12.5 (12.1–12.9)	338,717	76		0.25 (−2.08 to 2.58)
	Ultrasound	10.8 (8.5–13.1)	6719	1		−1.46 (−4.72 to 1.80)
Path length measure	Difference, Measured	11.9 (11.0–12.9)	129,958	6	0.31	0 (ref)
	Absolute, Calculated	12.7 (12.1–13.3)	49,243	19		0.81 (−0.26 to 1.88)
	Absolute, Measured	12.1 (11.1–13.2)	28,903	5		0.22 (−1.16 to 1.61)
	Difference, Calculated	11.9 (11.1–12.8)	42,090	8		−0.00 (−1.24 to 1.24)
	Not specified	12.6 (12.2–13.0)	106,256	40		0.68 (−0.33 to 1.68)
Region	Europe	10.7 (9.6–11.8)	14,355	4	0.0020	0 (ref)
	Asia	12.6 (12.2–12.9)	342,095	74		1.83 (0.71–2.94)
Study quality	High Quality	12.5 (12.1–12.9)	322,153	67	0.68	0 (ref)
	Other Quality	12.3 (11.6–13.1)	34,297	11		−0.16 (−0.94 to 0.61)
Study size (n)	2000+	12.3 (11.8–12.8)	325,430	35	0.077	0 (ref)
	500–2000	12.3 (11.8–12.8)	27,460	28		0.03 (−0.55 to 0.61)
	≤500	13.1 (12.4–13.8)	3560	15		0.79 (0.08–1.51)
Study year	≤2015	12.5 (12.1, 12.9)	335,545	65	0.35	0 (ref)
	>2015	12.2 (11.5, 12.9)	20,905	13		−0.34 (−1.05, 0.37)

Mean PWV indicates age-standardised average PWV values in meters per second and 95% confidence intervals. Number of participants and number of studies give the count of studies and participants used for each estimate in each subgroup. P (any diff.) is for any difference in standardised average PWV between category subgroups. No. = number of. Diff. = difference. Ref. = reference.

**Table 2:** Age-standardised cfPWV by world region, country, and other covariables.

Variable	Category	Mean PWV (95% CI)	No. participants	No. studies	P (any diff.)	Diff. to ref. category (95% CI)
All data		7.45 (7.11–7.79)	166,375	93		
Country	China	8.46 (7.68–9.24)	35,749	9	<0.0001	0 (ref)
	Algeria	7.79 (6.84–8.75)	424	2		−0.67 (−1.88 to 0.55)
	Angola	8.06 (6.44–9.69)	737	2		−0.39 (−2.19 to 1.41)
	Argentina	6.05 (3.79–8.31)	1710	1		−2.41 (−4.79 to −0.03)
	Australia	6.80 (5.17–8.43)	3619	2		−1.66 (−3.45 to 0.13)
	Austria	7.33 (5.68–8.98)	2696	2		−1.13 (−2.95 to 0.69)
	Belgium	5.77 (3.47–8.06)	2444	1		−2.69 (−5.10 to −0.28)
	Brazil	7.35 (6.56–8.14)	26,027	9		−1.10 (−2.19 to −0.02)
	Canada	5.88 (3.55–8.21)	315	1		−2.58 (−5.04 to −0.12)
	Czech Republic	6.65 (4.37–8.93)	1031	1		−1.81 (−4.20 to 0.59)
	Finland	6.03 (4.39–7.68)	1046	2		−2.43 (−4.23 to −0.62)
	France	8.69 (7.80–9.59)	5454	7		0.24 (−0.92 to 1.40)
	Germany	8.33 (6.06–10.59)	8456	1		−0.13 (−2.51 to 2.25)
	Greece	7.89 (6.74–9.04)	3651	4		−0.57 (−1.94 to 0.80)
	Hungary	7.23 (6.28–8.18)	445	1		−1.23 (−2.44 to −0.02)
	Iceland	8.98 (6.66–11.30)	940	1		0.52 (−1.91 to 2.96)
	India	6.63 (4.30–8.95)	1770	1		−1.83 (−4.28 to 0.62)
	Italy	7.44 (6.49–8.39)	9044	5		−1.02 (−2.22 to 0.19)
	Lithuania	7.44 (5.01–9.87)	355	1		−1.02 (−3.56 to 1.52)
	Macedonia	7.59 (5.32–9.86)	558	1		−0.87 (−3.25 to 1.52)
	Netherlands	8.11 (7.15–9.07)	8998	6		−0.35 (−1.56 to 0.87)
	Poland	9.42 (7.77–11.06)	920	2		0.96 (−0.85 to 2.76)
	Portugal	7.91 (6.58–9.23)	5877	3		−0.55 (−2.08 to 0.97)
	Russia	9.38 (7.10–11.66)	279	1		0.92 (−1.48 to 3.32)
	South Africa	6.36 (5.20–7.52)	2876	4		−2.10 (−3.48 to −0.71)
	South Korea	6.18 (5.02–7.34)	1608	4		−2.28 (−3.65 to −0.90)
	Spain	5.66 (3.36–7.95)	265	1		−2.80 (−5.21 to −0.39)
	Sweden	7.05 (4.74–9.35)	3056	1		−1.41 (−3.83 to 1.00)
	Switzerland	6.44 (4.83–8.06)	2039	2		−2.01 (−3.79 to −0.24)
	United Kingdom	6.37 (5.32–7.42)	11,069	5		−2.09 (−3.37 to −0.80)
	United States of America	7.22 (6.51–7.93)	19,374	11		−1.24 (−2.26 to −0.21)
	Uruguay	7.04 (4.79–9.29)	3543	1		−1.42 (−3.79 to 0.96)
Country income	High Income	7.32 (6.98–7.66)	96,245	64	<0.0001	0 (ref)
	Other Income	7.74 (7.38–8.09)	70,130	30		0.41 (0.26–0.57)
Device	Sphygmocor	7.00 (6.55, 7.46)	53,799	36	0.0008	0 (ref)
	Complior	8.48 (7.97, 8.98)	45,591	28		1.47 (0.83, 2.12)
	Omron	6.77 (5.49, 8.05)	6382	4		−0.23 (−1.57, 1.11)
	Parks Doppler	6.62 (5.35, 7.89)	10,268	4		−0.38 (−1.71, 0.95)
	Pulsepen	8.06 (6.77, 9.35)	18,977	4		1.06 (−0.29, 2.41)
	Vasera	6.75 (4.17, 9.33)	339	1		−0.26 (−2.86, 2.35)
	Vicorder	6.76 (5.71, 7.82)	15,815	6		−0.24 (−1.37, 0.89)
	Other	7.00 (6.18, 7.81)	15,204	10		−0.01 (−0.92, 0.90)
Measurement method	Tonometry	7.62 (7.25–7.98)	132,698	76	0.080	0 (ref)
	Oscillometry	6.77 (5.90–7.64)	22,536	11		−0.85 (−1.76 to 0.07)
	Ultrasound	6.67 (5.51–7.83)	11,141	6		−0.94 (−2.14 to 0.25)
Path length measure	Difference, Measured	6.94 (6.39–7.49)	58,595	28	0.13	0 (ref)
	Absolute, Calculated	6.77 (3.93–9.61)	339	1		−0.17 (−3.05 to 2.71)
	Absolute, Measured	7.88 (7.31–8.44)	50,391	27		0.94 (0.18–1.70)
	Absolute, Measured, Corrected Or Alternative Distance	8.10 (6.95–9.25)	13,175	6		1.16 (−0.10 to 2.42)
	Not specified	7.46 (6.93–7.98)	43,875	31		0.52 (−0.22 to 1.25)
Region	Europe	7.50 (7.07–7.93)	68,623	47	<0.0001	0 (ref)
	Africa	7.91 (7.46–8.36)	4037	8		0.41 (0.25–0.57)
	Asia	7.56 (6.82–8.30)	42,746	16		0.06 (−0.77 to 0.88)
	North America	7.10 (6.25–7.95)	19,689	12		−0.40 (−1.32 to 0.53)
	South America	7.19 (6.30–8.08)	31,280	11		−0.31 (−1.28 to 0.65)
Study quality	High Quality	7.43 (7.06–7.79)	135,861	76	0.44	0 (ref)
	Other Quality	6.94 (5.75–8.14)	7097	6		−0.48 (−1.71 to 0.75)
	Unknown	7.89 (7.00–8.77)	23,417	11		0.46 (−0.48 to 1.40)
Study size (n)	2000+	7.71 (7.06–8.36)	104,436	21	0.52	(ref)
	500–2000	7.30 (6.87–7.73)	56,693	52		−0.41 (−1.15 to 0.34)
	≤500	7.58 (6.91–8.25)	5246	20		−0.13 (−1.04 to 0.77)
Study year	≤2015	7.52 (7.16, 7.89)	122,684	78	0.33	0 (ref)
	>2015	7.11 (6.35, 7.88)	43,691	15		−0.41 (−1.23, 0.42)

Presentation of results is as described in the legend to Table 1. No. = number of. Diff. = difference. Ref. = reference.

Oscillometry was the dominant method for measuring baPWV, while tonometry was the dominant method for cfPWV. Both methods yielded comparable PWV values (Tables 1 and 2), and devices from different manufacturers also tended to yield similar PWV values, except for Complior devices, which on average appeared to have higher cfPWV measurements than Sphygmocor devices. Although found differences may be due to differences in examined participants as opposed to differences in measurement devices, prior studies involving direct comparisons of measurement devices also found higher measured PWV with Complior devices.^16^ Whereas baPWV showed no appreciable differences according to how path length in the calculation of PWV was derived, with the different derivation methods depicted in the Appendix (p 4), cfPWV based on the difference between heart-carotid and heart-femoral distance was on average lower than cfPWV based on the absolute carotid-femoral distance (Table 2). When repeating the main analysis while excluding unpublished studies, results remained very similar and the main conclusions were unchanged (Appendix p 48).

No significant differences in age-standardised cfPWV or baPWV were observed according to study quality, study size, or study year.

## Discussion

Arterial stiffening is paramount to vascular ageing and is associated with atherosclerosis, vascular calcification and inflammation, diabetes and chronic kidney disease, microangiopathy, and genuine vascular ageing represented by smooth muscle cell senescence and extracellular matrix elastin loss.^2^ Further possible determinants of arterial stiffening include statin therapy,^17^ thyroid disorders, and autoimmune disease.^18^ PWV, a simple, non-invasive measure of arterial stiffness, is one of the strongest predictors of mortality and CVD independent of vascular risk factors.^4^^,^^19^^,^^20^ Arterial stiffening contributes to systolic hypertension, heart failure, atrial fibrillation,^6^ aortic aneurysm formation,^21^ coronary artery disease and stroke,^2^^,^^7^ is involved in elevated CVD risk related to preeclampsia and prematurity,^22^^,^^23^ and facilitates hypertensive end-organ damage as chronic kidney disease, lacunar stroke, and vascular cognitive impairment.^24^, ^25^, ^26^, ^27^

Here, we aggregated global PWV measurements to facilitate clinical use of PWV. In our meta-analysis, global average baPWV age-standardised to the age structure of the World Health Organization World 2000–2025 Standard Million population was 12.5 m/s, and cfPWV, 7.45 m/s. Country-specific age-standardised averages are provided in Fig. 3 and Tables 1 and 2. Global age- and sex-dependent PWV distributions are shown in Fig. 1, and country-specific distributions in the Appendix (pp 6–7). PWV distributions were characterised by the cross-sectional mean values continuously increasing from young adulthood to approximately age 60 years, with steeper increases during adolescence and at older ages coinciding with periods of physiological hormonal transitions. Although this study did not assess hormone levels, prior studies found free testosterone associated with higher aortic stiffness in men and oestradiol with lower stiffness in women.^28^ Before adolescence, cfPWV showed an approximately linear increase and no sex differences. Whereas males featured higher cfPWV and baPWV than females after adolescence, the sex difference was relatively constant for cfPWV over the remaining lifespan, but was greatest during young adulthood for baPWV and diminished afterwards due to accelerated increases in baPWV in females after age 40 years during menopause (Fig. 1).^29^ Sex differences in age-standardised PWV (especially cfPWV) were heterogeneous between countries and males featured higher PWV than females in all countries except Poland, Germany, Indonesia, and Hungary (Fig. 5). Age-dependent increases in PWV differed between countries and were steepest for cfPWV in the USA (Appendix p 7). Differences in PWV between countries may be due to differences in the prevalence of vascular risk factors, many of which are related to PWV,^11^ the impact of diet and exercise,^30^^,^^31^ heart rate,^32^ and racial or ethnic and genetic variation. Indeed, relevant heritability of cfPWV of approximately h^2^ = 0.4 was found in 1480 participants of the Framingham offspring cohort aged on average 60 years old.^33^ Although few of the studies provided information on the race or ethnicity of participants, cfPWV was highest in Africa (Table 2), which is consistent with previous reports of higher PWV in individuals of African American as compared to those of European descent.^34^

The most commonly used types of PWV are baPWV and cfPWV. Both are highly reproducible, with coefficients of variation in the range of 3.6%–7.4%.^35^, ^36^, ^37^ Measurement of PWV is reliable in the presence of atrial fibrillation,^32^ and bilateral severe peripheral artery disease prohibiting baPWV measurement is rare. In two large Chinese cohorts,^12^ 16/9897 (0.16%) and 159/77,973 (0.20%) of participants featured ankle-brachial index <0.9 on both sides (unpublished data). While baPWV is higher than cfPWV,^38^ both are correlated (r = 0.73)^38^ and are excellent predictors of cardiovascular events.^10^^,^^11^^,^^38^ Both cfPWV and baPWV measure the speed of the pulse pressure wave along the aorta and central medium-sized proximal muscular arteries, but baPWV additionally includes variability due to the wave speed along the lengthy muscular arteries of the leg. We found baPWV to vary relatively less than cfPWV (Fig. 3), which likely reflects the limited variation of muscular artery PWV with age.^39^ Measurement of baPWV is simpler than that of cfPWV, ideally requiring only the wrapping of blood pressure cuffs on extremities.^38^ Differences in PWV according to measurement method, device manufacturer, or path length measure used were not evident for baPWV (Table 1). For cfPWV, Complior devices and absolute path length measurements tended to yield higher PWV measurements (Table 2).

Two key findings of our study merit discussion: 1) Asian compared to European countries had substantially higher baPWV (+1.83 m/s) (Fig. 3 and Table 1). Higher baPWV is associated with augmented central systolic and pulse pressure and may contribute to higher risk of intracerebral haemorrhage and stroke due to small vessel disease in certain Asian countries and to the high risk of stroke in China.^40^^,^^41^ It is tempting to speculate that reducing arterial stiffness might improve prevention of stroke in these countries. Importantly, China was also among the countries with the highest cfPWV (Table 2). 2) High country income was robustly associated with both lower cfPWV and baPWV. This is in line with higher socio-economic status at the level of individuals being associated with slower age-related increases in PWV,^42^ and with the fact that high-income countries feature lower incidence of and mortality from major cardiovascular events.^43^

Strengths of this study include its unprecedented size and global scope. It included most published and several unpublished studies on cfPWV and baPWV values, in part with individual data or summary statistics provided by members of the Global PWV Study Group, and thereby offers a hitherto unavailable compendium of global and regional PWV trajectories and distributions. One earlier analysis reported reference and normal values of PWV based on data from 11,092 individuals in 8 European countries without overt CVD or certain CVD risk factors, and by presence and severity of arterial hypertension.^44^ In contrast, the reference values provided here are intended to be representative of the general population of each country considered and include participants with CVD and with risk factors in the prevalences present in each of these populations. Reference values may enable researchers and clinicians to put newly measured PWV values into the context of the relevant population. Weaknesses include that for many countries representative data on PWV were not available, that part of our data were extracted from publications with varying quality, and that we did not delineate the relative contributions to regional differences in PWV of genetic and environmental factors.

In summary, herein we provide global and regional age- and sex-dependent distributions and reference values of baPWV and cfPWV in generally healthy people, which may aid increased clinical use of PWV as a measure of vascular ageing, predictor of hypertensive end-organ damage, cardiovascular disease, and death. Smaller intervention studies have reported improvement of arterial stiffness by caloric restriction,^45^ diet^31^ (e.g., low-sodium high-potassium diet)^46^ and dietary supplements (e.g., curcumin, and nicotinamide), exercise,^30^^,^^45^ and drugs inhibiting angiotensin-II or reducing systemic inflammation. Arterial stiffness is an appealing target for prevention, however, large-scale intervention studies are needed to guide clinicians.

## Contributors

SK, YL, RP, and QX conceptualised and designed the analysis. YL, SJK, JW, and SK were responsible for data collection. RP, YL, SJK, and SK drafted the manuscript. JW and QX revised the manuscript. RP, YL, JW, SK, and SJK verified the data underlying the work. RP and SK performed statistical analyses and prepared the figures. SJK and JW performed quality assessments of included studies.

All authors contributed substantially to the conception of the work or the acquisition or interpretation of data and to revising the work for important intellectual content. All authors had full access to all the data in the study. All authors approve of the final version to be published and agree to be accountable for the decision to submit for publication and for all aspects of the work in ensuring that questions related to the accuracy or integrity of any part of the work are appropriately investigated and resolved.

## Data sharing statement

Data that were extracted from published reports are available from the authors upon reasonable request with publication. Data from individual included studies (listed in Appendix pp 9–35) that are not publicly available may be available for use in independent scientific research upon reasonable request to the relevant member(s) of the Global PWV Study Group (listed below). Data may be provided following review and approval of a research proposal (including a statistical analysis plan) and completion of a data sharing agreement.

## Declaration of interests

We declare no competing interests.

## Appendix A Supplementary data

Supplementary Appendix

Protocol for a systematic review and meta analysis

Supplementary Appendix to Protocol
