# Supplementary material for: Global distributions of age- and sex-related arterial stiffness: systematic review and meta-analysis of 167 studies with 509,743 participants
Source: eBioMedicine. 2023 May 23;92:104619. doi: 10.1016/j.ebiom.2023.104619 (PMC10327869; doi:10.1016/j.ebiom.2023.104619)
Supplement: Supplementary Appendix [file mmc1.pdf]

<u>Supplementary Appendix</u>

Global distributions of age- and sex-related arterial stiffness: systematic review and meta-analysis of 167 studies with 509743 participants

Yao Lu, Sophia J. Kiechl, Jie Wang, Qingbo Xu, Stefan Kiechl, Raimund Pechlaner, on behalf of the *Global Pulse Wave Velocity Study Group*

Table of Contents

Supplemental Methods....................3

Supplemental Figures....................4

Supplemental Figure 1: Distance measurement in pulse-wave velocity calculation....................4
Supplemental Figure 2: Study flow chart....................5
Supplemental Figure 3a: Age- and sex- dependent distributions of baPWV by country....................6
Supplemental Figure 3b: Age- and sex- dependent distributions of cfPWV by country....................7

Supplemental Tables....................8

Supplemental Table 1: Literature search strategy....................8
Supplemental Table 2: Description of studies included....................34
Supplemental Table 3: Quality rating of included studies....................40
Supplemental Table 4a: Reference values for baPWV by age, sex and country....................42
Supplemental Table 4b: Reference values for cfPWV by age, sex and country....................46

References....................47

Supplemental Methods

Pre-processing of individual participant data

Individual participant data points that indicated PWV > 55 m/s or < 0 m/s were excluded, and 0.1% of data were trimmed from both extremes of the overall distribution to remove outliers.

Data transformation of data extracted from the literature

Data extracted from the literature that were not given as means and standard deviations (SDs) were all given as quantile measures, in particular medians and interquartile ranges, and means and SDs were derived from quantile measures using single regression imputation based on models fitted in individual participant data, utilizing all information on the distributions of PWV available.

For extracted data that were not reported in decades of age, data were pooled into decades of age by weighted averaging where age granularity was finer than decades. Where age granularity was coarser than decades, decade groups were created and the total number of subjects was distributed across decade groups proportionally to the World Health Organization World 2000-2025 Standard Million population where age was reported as a range, or proportional to the central 80% of probability mass of a normal distribution defined by reported means and SDs where age was reported by mean and SD. Values of means and SDs of PWV in the resulting decade categories were derived such that the weighted means of mean and SD of PWV over decade groups were identical to those reported by each publication, and simultaneously such that relative differences in mean and SD of PWV between decile groups were consistent with those observed in individual-participant and summary data received in decade groups from collaborators. For this, general purpose optimization was used to align the ratios of mean and SD of PWV between any two contiguous decile groups with the median of the ratios observed in studies received from collaborators, using as reference either studies from the same country as that for which decile categories were created, or, when such studies were not available, the same world region.

Generalized Additive Models for Location, Scale and Shape (GAMLSS)

Distributions of PWV conditional on age, sex and region were fitted using flexible semi-parametric GAMLSS models within the framework of the R gamlss package[1].

The distribution family most appropriate for the current data was determined separately for cfPWV and baPWV by fitting all positive real-valued distributions available in the package and minimizing Bayesian Information Criterion. The Box-Cox Cole and Green (BCCG) distribution was consistently best-fitting for both cfPWV and baPWV. Fit of the optimal distribution family was investigated using worm plots (de-trended Q-Q plots) and plots of residuals against fitted values and against age.

The three parameters μ, σ, and ν that govern location, scale, and skewness of the BCCG distribution conditional on age were fitted using identity, log, and identity link functions and were fitted using the same model formula for all three moments. The formula consisted of separate smooth P-spline fits of age for each sex by means of penalised varying coefficient terms[2] and random intercepts for individual studies.

Only individual participant data were used to fit GAMLSS models. When there were fewer than 100 subjects within any subgroup defined according to decade of age, sex, and country for carotid-femoral and brachial-ankle PWV individually, data belonging to that subgroup were not considered and neither were countries with fewer than three distinct age decade groups available.

To avoid over-weighting of countries for which the largest datasets were available and to improve convergence of the iterative model fitting algorithm when estimating results for the total pooled age- and sex-conditional PWV distributions, only for this analysis were models fitted to stratified subsamples of the data. Subsamples included the lower of all subjects available and 1000 subjects, but no fewer than 100 subjects, for each subgroup defined as above, performing weighted sampling to achieve approximately equal contributions of the different studies with data available for each subgroup.

Model fit was investigated by comparing fitted and empirical quantiles using marginal and conditional worm plots and by comparing fitted and sample PWV values. Statistical moments of the fitted conditional distributions were investigated for smoothness of age trajectories and for consistency of differences by age, sex and country.

Supplemental Figures

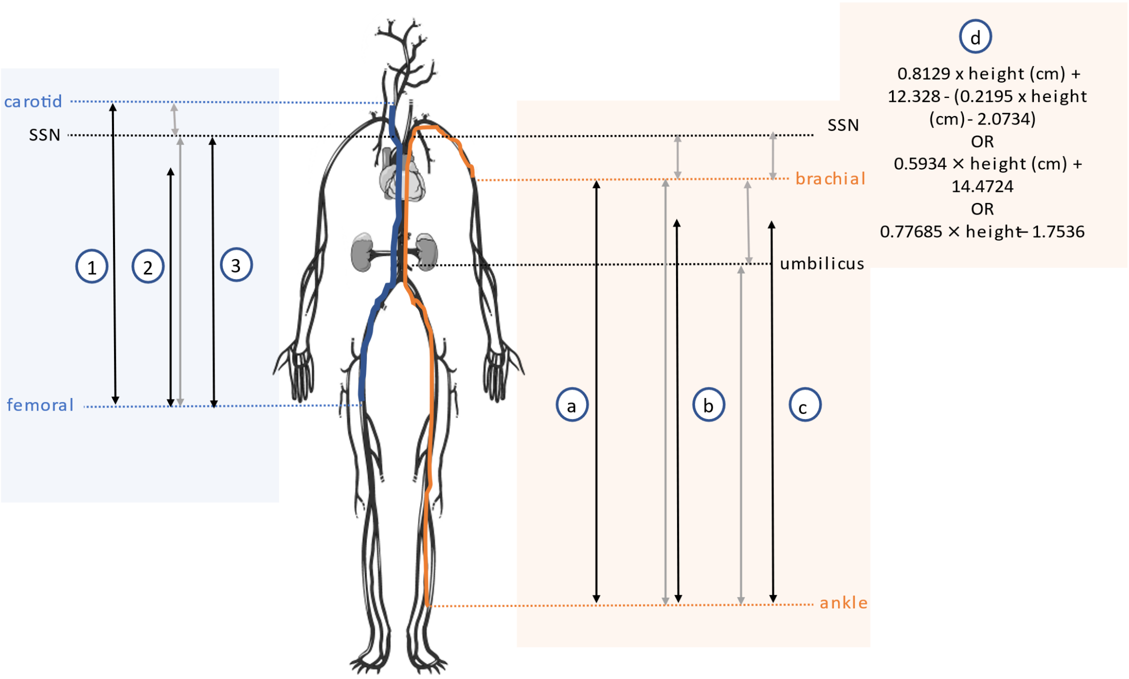

Supplemental Figure 1: Distance measurement in carotid-femoral and brachial-ankle pulse wave velocity calculation.

The path length used in PWV velocity calculation often involved differences in distances between landmarks (2, b, c) to account for the pulse pressure wave traveling in different directions from the heart to the two measuring sites (eg, suprasternal notch-femoral artery minus suprasternal notch-carotid artery for cfPWV (2)), but absolute distances were also used (1, a) (eg carotid-femoral artery (1)). The distances used were partly measured on the body surface (1-3, a-c) and partly derived from body height using regression equations (d), resulting in 4 principal ways to derive path length. A fifth path length derivation category was used for studies that applied a corrective multiplicative constant to path length or used non-standard distances (eg heart-femoral).

This figure includes content from Servier Medical Art by Servier, licensed under a Creative Commons Attribution 3·0 unported license.

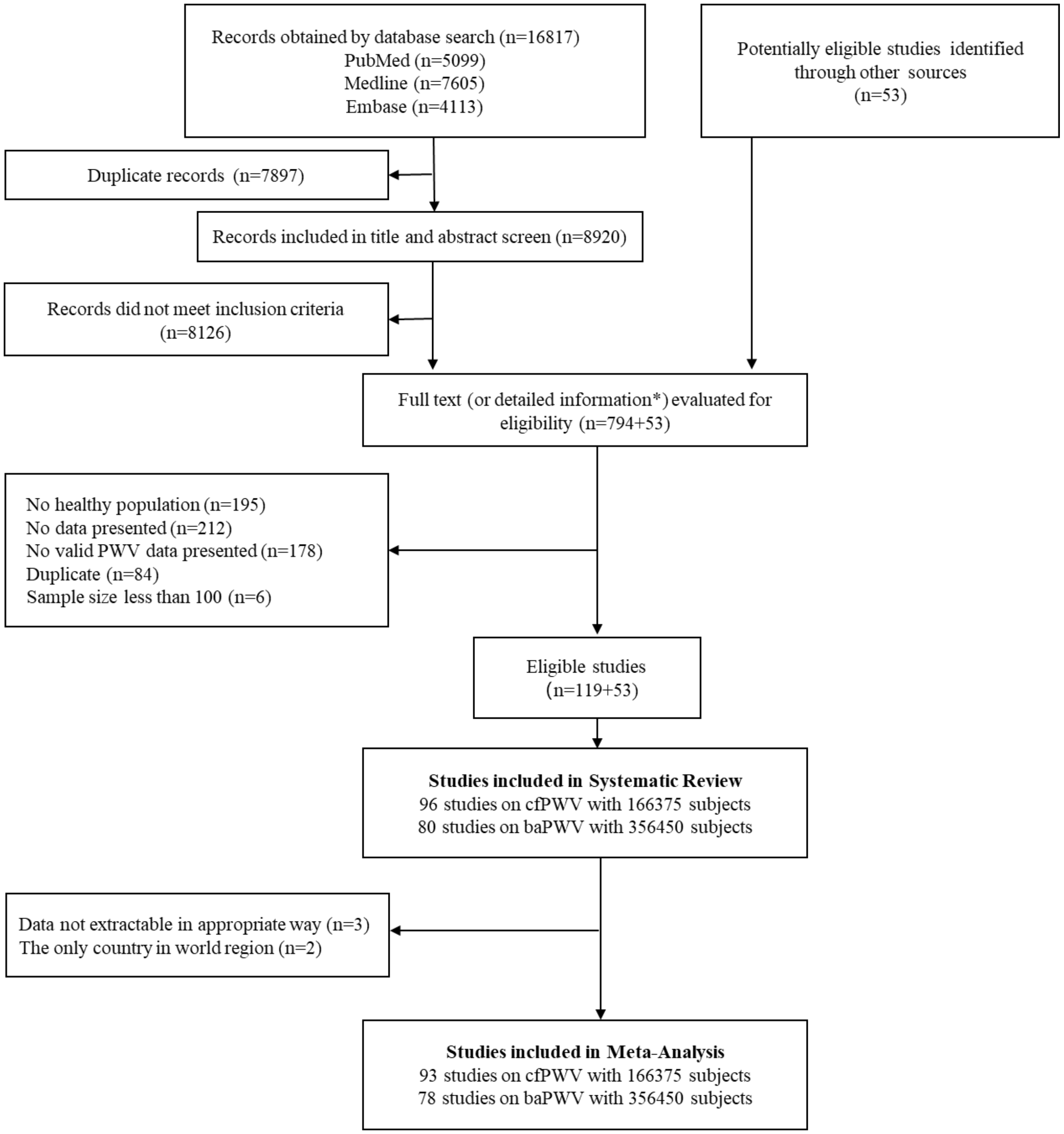

***Supplemental Figure 2:* Study flow chart.**

Potentially eligible studies identified through other sources included studies identified within the references of included studies or suggested by collaborators.

* data from 8 of these studies were not fully published and their eligibility assessment was based on information provided by the collaborators study data were received from.

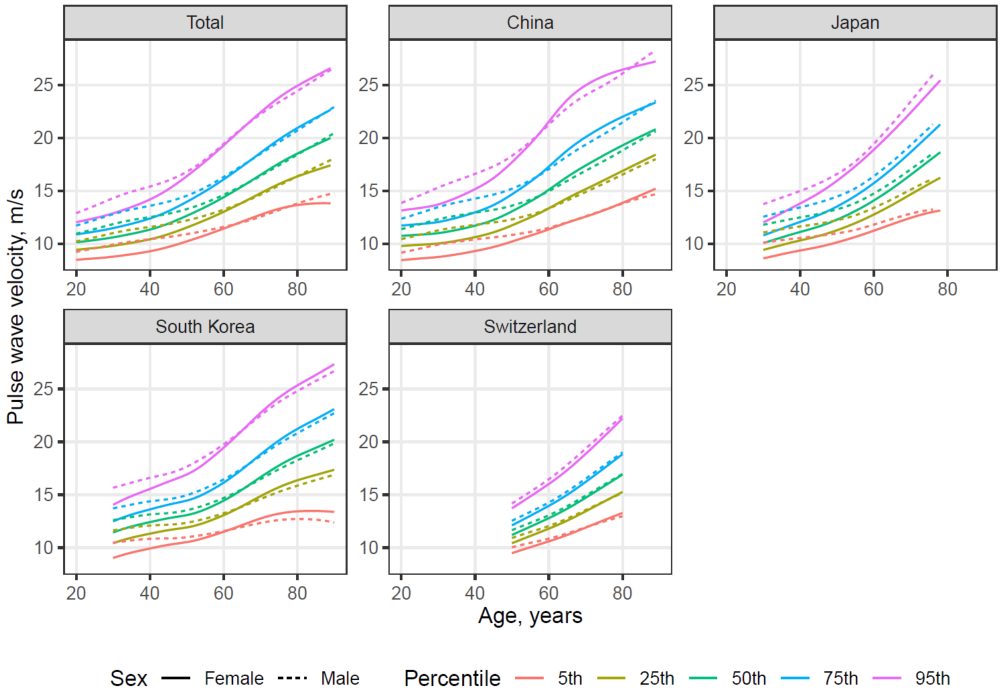

***Supplemental Figure 3a*: Age- and sex- dependent distributions of baPWV by country.**

Asian countries featured similar baPWV distributions, with little variability between countries.

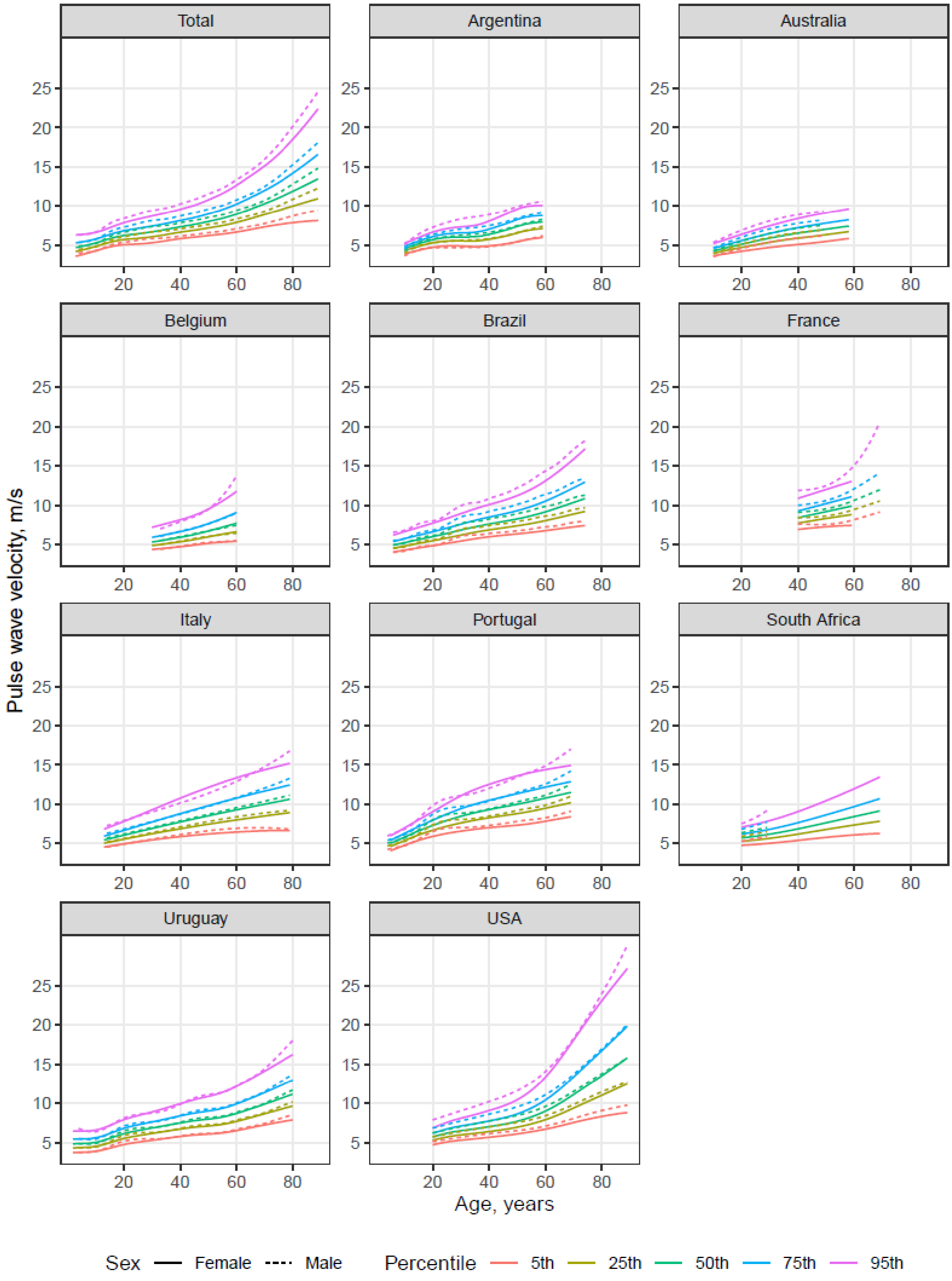

Supplemental Figure 3b: **Age- and sex- dependent distributions of cfPWV by country.**

Country-specific differences were more important than differences according to world region for cfPWV.

Supplemental Tables

Database	Search terms	Results
**PubMed**	(((arterial stiffness[Title/Abstract]) OR (arterial stiffening[Title/Abstract]) OR (artery stiffness[Title/Abstract]) OR (artery stiffening[Title/Abstract]) OR (vascular stiffness[Title/Abstract]) OR (vascular stiffening[Title/Abstract]) OR (aortic stiffness[Title/Abstract]) OR (aortic stiffening[Title/Abstract])) AND ((epidemiology[Title/Abstract]) OR (prevalence[Title/Abstract]))) OR (((PWV[Title/Abstract]) OR (pulse wave velocity[Title/Abstract]) OR (baPWV[Title/Abstract]) OR (brachial-ankle pulse wave velocity[Title/Abstract]) OR (cfPWV[Title/Abstract]) OR (carotid- femoral pulse wave velocity[Title/Abstract])) AND ((value[Title/Abstract]) OR (values[Title/Abstract]) OR (estimation[Title/Abstract]) OR (estimated[Title/Abstract]) OR (distribution[Title/Abstract]) OR (determinant[Title/Abstract]) OR (determinants[Title/Abstract]) OR (determination [Title/Abstract]) OR (assessment[Title/Abstract])))	5099
**MEDLINE**	"1 TI=(arterial stiffness OR arterial stiffening OR artery stiffness OR artery stiffening OR vascular stiffness OR vascular stiffening OR aortic stiffness OR aortic stiffening) OR AB=(arterial stiffness OR arterial stiffening OR artery stiffness OR artery stiffening OR vascular stiffness OR vascular stiffening OR aortic stiffness OR aortic stiffening)<br>2 TI=(prevalence OR epidemiology) OR AB=(prevalence OR epidemiology)<br>3 1 AND 2<br>4 TI=(PWV OR pulse wave velocity OR baPWV OR brachial-ankle pulse wave velocity OR cfPWV OR carotid-femoral pulse wave velocity)OR AB=(PWV OR pulse wave velocity OR baPWV OR brachial-ankle pulse wave velocity OR cfPWV OR carotid-femoral pulse wave velocity)<br>5 TI=(value OR values OR estimation OR estimated OR distribution OR determinant OR determinants OR determination OR assessment)OR AB=(value OR values OR estimation OR estimated OR distribution OR determinant OR determinants OR determination OR assessment)<br>6 4 AND 5<br>7 3 OR 6<br>8 (#3 OR #6) AND limit 7 to (Humans) AND limit 7 to (MEDLINE)	7605
**EMBASE**	1 (arterial-stiffness OR arterial-stiffening OR artery-stiffness OR artery-stiffening OR vascular-stiffness OR vascular-stiffening OR aortic-stiffness OR aortic-stiffening):ab,ti<br>2 (prevalence OR epidemiology) :ab,ti<br>3 #1 AND #2<br>4 (PWV OR pulse-wave-velocity OR baPWV OR brachial-ankle-pulse-wave-velocity OR cfPWV OR carotid-femoral-pulse-wave-velocity):ab,ti<br>5 (value OR values OR estimation OR estimated OR distribution OR determinant OR determinants OR determination OR assessment):ab,ti<br>6 #4 and #5<br>7 #3 OR #6<br>8 (#3 OR #6) AND ([article]/lim OR [article in press]/lim OR [conference paper]/lim OR [short survey]/lim) AND [humans]/lim AND [embase]/lim	4113

Supplemental Table 1: Literature search strategy.

Search was conducted from database inception to August 24, 2020, without language restrictions. Data on generally healthy subjects mainly included data derived from community-based observational studies. Reviews, case reports, repeated studies, patient series or studies with sample sizes below 100 were not considered.

Study	Population	Study type	PWV type	Data source	n total	n male	n female	Country	Survey year	Measurement method	Device name	Study name	Paper title	Publication year
**Achimastos et al (2007)[3]**	Adults without history of CVD, mean age 45·3±15·5 years	Cross-sectional study	Carotid-femoral	Extracted	423	297	126	Greece	2001	Tonometry	Complior		Arterial Stiffness: Determinants and Relationship to the Metabolic Syndrome	2007
**Ai et al (2011)[4]**	Adults from the Pudong New Districts of Shanghai aged 30–85 years (with and without cardiovascular risk factors)	Cross-sectional study	Brachial-ankle	Extracted	566	114	452	China	2009	Oscillometry	BP203RPE-II [VP-1000], Omron		Reference value of brachial-ankle pulse wave velocity for the eastern Chinese population and potential influencing factors	2011
**Araghi et al (2020)[5]**	Adults [(former) British civil servants] aged 65·1±5·2 years	Longitudinal cohort study	Carotid-femoral	Summary	4347	3237	1110	UK	2007-2009	Tonometry	SphygmoCor	Whitehall II	Association of aortic stiffness with cognitive decline: Whitehall II longitudinal cohort study	2020
**Avramovski et al (2016)[6]**	Adults from the general population aged 37–80 years	Longitudinal cohort study	Carotid-femoral	Summary	558	306	252	Macedonia	2012-2015	Ultrasound	Toshiba SSA-340A, Toshiba Medical System Corporation, Tokyo, Japan		Bone Strength and Arterial Stiffness Impact on Cardiovascular Mortality in a General Population	2016
**Baier et al (2018)[7]**	Adults from the general population aged 18–80 years	Longitudinal cohort study	Carotid-femoral, Brachial-ankle	Summary	16934	8246	8688	Germany	2011-2014	Oscillometry	Vicorder	LIFE-Adult	Parameters of pulse wave velocity: determinants and reference values assessed in the population-based study LIFE-Adult	2018
**Baldo et al (2017)[8]**	Adults without history of CVD aged 35–74 years (active or retired civil servants)	Longitudinal cohort study	Carotid-femoral	Individual	14722	6734	7988	Brazil	2008-2010	Tonometry	Complior	ELSA-Brasil	Racial Differences in Arterial Stiffness are Mainly Determined by Blood Pressure Levels: Results From the ELSA-Brasil Study	2017
**Benetos et al (2009)[9]**	Healthy adults aged 60–85 years	Cross-sectional study	Carotid-femoral	Individual	260	179	81	France	2005-2006	Tonometry	PulsePen	ERA	Effects of lean and fat mass on bone mineral density and arterial stiffness in elderly men	2009

Study	Population	Study type	PWV type	Data source	n total	n male	n female	Country	Survey year	Measurement method	Device name	Study name	Paper title	Publication year
**Bérard et al (2013)**[10]	Healthy adults aged 35–64 years (stratified random sample)	Longitudinal cohort study	Carotid-femoral	Individual	1007	528	479	France	1995-1997	Tonometry	Complior	Third Toulouse MONICA survey (1995-1997)	Pulse wave velocity, pulse pressure and number of carotid or femoral plaques improve prediction of cardiovascular death in a population at low risk	2013
**Bia et al (2021)**[11]	Children aged 3–17 years, adults aged 18–84 years from the general population without history of CVD	Cross-sectional study	Carotid-femoral	Individual	3543	1833	1710	Uruguay	2010-2020	Tonometry	SphygmoCor	CUiiDARTE Project (CUiiDARTE: Centro Universitario de Investigación, Innovación y Diagnóstico Arterial)	Physiological Age- and Sex-Related Profiles for Local (Aortic) and Regional (Carotid-Femoral, Carotid-Radial) Pulse Wave Velocity and Center-to-Periphery Stiffness Gradient, with and without Blood Pressure Adjustments: Reference Intervals and Agreement between Methods in Healthy Subjects (3-84 Years).	2021
**Bian et al (2012)**[12]	Adults from the general population without history of CVD aged 58·2±12·4 years	Cross-sectional study	Carotid-femoral	Extracted	2374	1137	1237	China	2007	Tonometry	Complior		Serum Uric Acid Level and Diverse Impacts on Regional Arterial Stiffness and Wave Reflection	2012
**Botha et al (2021)**[13]	Healthy black and white adults aged 20–30 years	Longitudinal cohort study	Carotid-femoral	Individual	1231	592	639	South Africa	2013-2017	Tonometry	SphygmoCor	African-PREDICT	Comparing the associations of clinic vs. ambulatory blood pressure with subclinical organ damage in young healthy adults: the AfricanPREDICT study	2021
**Cecelja et al (2011)**[14]	Female Caucasian twins aged 18–91 years	Cross-sectional study	Carotid-femoral	Summary	3041		3041	UK	2006	Tonometry	Sphygmocor	Twins UK Cohort	Arterial Stiffening Relates to Arterial Calcification But Not to Noncalcified Atheroma in Women: A Twin Study	2011

Study	Population	Study type	PWV type	Data source	n total	n male	n female	Country	Survey year	Measurement method	Device name	Study name	Paper title	Publication year
**Cecelja et al (2020)**[15]	Healthy adults aged 20–24 years	Longitudinal cohort study	Carotid-femoral	Extracted	1770	858	575	India	2009-2010	Oscillometry	Vicorder	Andhra Pradesh Children and Parents Study	Association of pulse wave velocity and intima-media thickness with cardiovascular risk factors in young adults	2020
**Ceponiene et al (2015)**[16]	Adults aged between 48–49 years	Longitudinal cohort study	Carotid-femoral	Individual	355	157	198	Lithuania	2012	Tonometry	SphygmoCor	The Kaunas Cardiovascular Risk Cohort Study	Associations between risk factors in childhood (12–13 years) and adulthood (48–49 years) and subclinical atherosclerosis: the Kaunas Cardiovascular Risk Cohort Study	2015
**Chen et al (2018)**[17]	Community–based sample of adults aged 61·2±9·5 years	Cross-sectional study	Carotid-femoral	Extracted	16644			China	2016	Tonometry	PulsePen		Association between serum total homocysteine and arterial stiffness in adults: a community-based study	2018
**Cho et al (2020)**[18]	Healthy adults aged 20–66 years	Cross-sectional study	Brachial-ankle	Individual	191	95	96	South Korea	2007-2008	Oscillometry	Omron VP-2000	Korean PWV-PTT Comparison Study	A Comparative Study of Brachial–Ankle Pulse Wave Velocity and Heart–Finger Pulse Wave Velocity in Korean Adults	2020
**Choi et al (2010)**[19]	Adults from the general population aged 50–94 years	Longitudinal cohort study	Brachial-ankle	Individual	9084	3630	5454	South Korea	2007-2009	Oscillometry	Omron VP-1000	The Dong-gu Study	Cohort Profile: The Namwon Study and the Dong-gu Study	2010
**Choi et al (2017)**[20]	Healthy adults aged 20–80 years	Cross-sectional study	Brachial-ankle	Extracted	2705	1228	1477	South Korea	2006-2013	Oscillometry	Colin VP-1000		Hyperuricemia and risk of increased arterial stiffness in healthy women based on health screening in Korean population	2017
**Chou et al (2015)**[21]	Hospital employees aged 40·1 ± 8·4 years	Cross-sectional study	Brachial-ankle	Extracted	576	85	491	China	2013	Oscillometry	VaSera VS-1000		Work-Related Psychosocial Hazards and Arteriosclerosis A Cross-Sectional Study Among Medical Employees in a Regional Hospital in Taiwan	2015

Study	Population	Study type	PWV type	Data source	n total	n male	n female	Country	Survey year	Measurement method	Device name	Study name	Paper title	Publication year
**Chuang et al (2005)[22]**	Community–based sample of adults aged 30–91 years	Cross-sectional study	Brachial-ankle	Summary	2210	1152	1058	China	2002	Oscillometry	Colin VP-1000	Kinmen Study	Combined use of brachial-ankle pulse wave velocity and ankle-brachial index for fast assessment of arteriosclerosis and atherosclerosis in a community	2005
**Cicero et al (2020)[23]**	Community–based sample of adults aged 14–84 years without history of CVD	Longitudinal cohort study	Carotid-femoral	Individual	1505	722	783	Italy	1972-2021*	Oscillometry	Vicorder	Brisighella Heart Study	Awareness of major cardiovascular risk factors and its relationship with markers of vascular aging: Data from the Brisighella Heart Study	2020
**The Lifelong Determinants of Obesity, Precursors of Chronic Diseases, Human Capital and Mental Health Study**	Community–based sample of adults aged 21–23 years	Cross-sectional study	Carotid-femoral	Individual	992	421	571	Brazil	2016-2017	Tonometry	Sphygmocor	The Lifelong Determinants of Obesity, Precursors of Chronic Diseases, Human Capital and Mental Health Study	Not published	
**Cruz et al (2020)[24]**	Community–based sample of adults aged 25–64 years	Cross-sectional study	Carotid-femoral	Individual	1609	734	875	Brazil	1999-2001	Tonometry	Complior		Arterial stiffness in black adults from Angola and Brazil	2020
**Cunha et al (2015)[25]**	Community–based sample of adults aged 18–96 years (stratified)	Longitudinal cohort study	Carotid-femoral	Extracted	1754	780	974	Portugal	2006	Tonometry	SphygmoCor		Pulse wave velocity distribution in a cohort study: fromarterial stiffness to early vascular aging	2015
**De Mendonça et al (2018)[26]**	Adults aged 69·2 ± 7·0 years	Longitudinal cohort study	Carotid-femoral	Extracted	1192			Brazil	2014-2015	Tonometry	SphygmoCor	EVOPIU	Arterial stiffness in elderly patients with normotension and hypertension in Brazil	2018
**De Oliveira Alvim et al (2015)[27]**	Community–based sample of adults aged 18–102 years	Longitudinal cohort study	Carotid-femoral	Summary	2155	864	1291	Brazil	2010-2013	Tonometry	Complior	Baependi Heart Study	Cohort profile: the Baependi Heart Study —a family-based, highly admixed cohort study in a rural Brazilian town.	2015

Study	Population	Study type	PWV type	Data source	n total	n male	n female	Country	Survey year	Measurement method	Device name	Study name	Paper title	Publication year
**Del Giorno et al (2021)[28]**	Adults from the general population aged 52 (43–60); median (IQR) (random sample)	Cross-sectional study	Carotid-femoral	Extracted	1037			Switzerland	2017-2018	Tonometry	SphygmoCor	The Ticino epidemiological stiffness (TEST) study	Comparing oscillometric and tonometric methods to assess pulse wave velocity: a population-based study	2021
**Diaz et al (2018)[29]**	Healthy children aged 9–17 years, healthy adults aged 18–87 years	Cross-sectional study	Carotid-femoral	Individual	1710	1022	688	Argentina	2010-2013	Tonometry	Arteriometer Motorola	Tandil Vela Project	Reference intervals and percentiles for carotid-femoral pulse wave velocity in a healthy population aged between 9 and 87 years	2018
**Ellins et al (2017)[30]**	Adults aged 72–92 years	Longitudinal cohort study	Carotid-femoral	Summary	1577	1577		UK	2010-2012	Oscillometry	Vicorder	British Regional Heart Study	Arterial pathophysiology and comparison of two devices for pulse wave velocity assessment in elderly men: the British regional heart study	2017
**The EVA study**	Community–based sample of adolescents aged 16–23 years	Longitudinal study	Carotid-femoral	Individual	1468	648	820	Austria	2016-2018	Tonometry	Complior	The Early Vascular Ageing (EVA) Study	Not published	
**The EVA4YOU study**	Community–based sample of adolescents aged 14–29 years	Cross-sectional study	Carotid-femoral	Individual	1228	498	730	Austria	2021-2022	Oscillometry	Vicorder	Early Vascular Ageing in the YOUth (EVA4YOU)	Not published	
**Fan et al (2017)[31]**	Community–based sample of adults aged 55·4±10·0 years (random sample)	Cross-sectional study	Brachial-ankle	Extracted	10281			China	2014	Oscillometry	Omron BP-203RPEⅢ		Urinary stone disease and cardiovascular disease risk in a rural Chinese population	2017
**Foraster et al (2017)[32]**	Population–based sample of adults aged 50–80 years	Longitudinal cohort study	Brachial-ankle	Individual	3062	1536	1526	Switzerland	1991-2017	Oscillometry	Vasera VS-1500N	SAPALDIA	Exposure to Road, Railway, and Aircraft Noise and Arterial Stiffness in the SAPALDIA Study: Annual Average Noise Levels and Temporal Noise Characteristics	2017

Study	Population	Study type	PWV type	Data source	n total	n male	n female	Country	Survey year	Measurement method	Device name	Study name	Paper title	Publication year
**Fu et al (2015)**[33]	Community–based sample of adults aged 21–96 years	Cross-sectional study	Carotid-femoral	Extracted	1540			China	2007-2009	Tonometry	Complior		Multimarker Analysis for New Biomarkers in Relation to Central Arterial Stiffness and Hemodynamics in a Chinese Community-Dwelling Population	2015
**Fujiwara et al (2018)**[34]	Community–based sample of children aged 12–15 years	Longitudinal cohort study	Brachial-ankle	Extracted	1729	930	799	Japan	2006-2008	Oscillometry	Omron BP-203RPEIII		Arterial stiffness in junior high school students: Longitudinal observations	2017
**Fukuda et al (2014)**[35]	Community–based sample of adults aged 24–84 years	Cross-sectional study	Brachial-ankle	Individual	909	590	319	Japan	2004-2012	Oscillometry	automatic waveform analyzer (Colin Medical Technology, Komaki, Japan)		Association between serum γ-glutamyltranspeptidase and atherosclerosis: a population-based cross-sectional study	2014
**Fukuhara et al (2006)**[36]	Adults aged 85 years	Cross-sectional study	Brachial-ankle	Extracted	203			Japan	2001	Oscillometry	VaSera VS-1000	8020 Data Bank Survey	Prediction of Cognitive Function by Arterial Stiffness in the Very Elderly	2006
**Gao et al (2015)**[37]	Adults aged 53·66±10·97 years with no history of CVD	Longitudinal cohort study	Brachial-ankle	Extracted	3950			China	2010-2011	Oscillometry	Omron BP-203RPE III		Changes in cardiovascular health score and atherosclerosis progression in middle-aged and older persons in China: a cohort study	2015
**Gomez-Sanchez et al (2016)**[38]	Adults aged 36–74 years with intermediate/moderate cardiovascular risk	Longitudinal cohort study	Brachial-ankle	Extracted	2315			Spain	2011-2013	Oscillometry	VaSera VS-1500	The MARK study	Association of metabolic syndrome and its components with arterial stiffness in Caucasian subjects of the MARK study: a cross-sectional trial	2016
**Gomez-Sanchez et al (2020)**[39]	Adults aged 34–77 years	Cross-sectional study	Brachial-ankle	Individual	500	249	251	Spain	2016-2017	Oscillometry	Vasera VS-1500	EVA study	Reference values of arterial stiffness parameters and their association with cardiovascular risk factors in the Spanish population	2020

Study	Population	Study type	PWV type	Data source	n total	n male	n female	Country	Survey year	Measurement method	Device name	Study name	Paper title	Publication year
**Guo et al (2020)[40]**	Community–based sample of adults aged 75·0 ± 6·5 years	Cross-sectional study	Carotid-femoral	Extracted	1272			China	2009	Tonometry	SphygmoCor		Difference in the risk profiles of carotid-femoral pulse wave velocity: results from two community-based studies in China and Sweden	2020
**Hanis et al (2016)[41]**	Adults aged 31–86 years	Longitudinal cohort study	Carotid-femoral	Individual	728	201	527	USA	2010-2014	Tonometry	SphygmoCor		Beyond type 2 diabetes, obesity and hypertension: an axis including sleep apnea, left ventricular hypertrophy, endothelial dysfunction, and aortic stiffness among Mexican Americans in Starr County, Texas	2016
**Haraguchi et al (2019)[42]**	Adults aged 39–75 years	Cross-sectional study	Brachial-ankle	Individual	3868	1381	2487	Japan	2013-2018	Oscillometry	Omron BP-203RPE II	The Japan Multi-Institutional Collaborative Cohort Study in Kyoto Field	Assessment of anthropometric indices other than BMI to evaluate arterial stiffness	2019
**Higashiura et al (2004)[43]**	Adults aged 18–90 years without CVD	Cross-sectional study	Brachial-ankle	Extracted	382			Japan	1999	Oscillometry	Colin		Correlations of adiponectin level with insulin resistance and atherosclerosis in Japanese male populations	2004
**Hou et al (2018)[44]**	Adults aged 28–42 years	Longitudinal cohort study	Carotid-femoral, Brachial-ankle	Summary	2306	1232	1074	China	2010-2011	Oscillometry	Omron BP203RPE-II	Beijing Blood Pressure Cohort Study	Childhood body mass index and blood pressure in prediction of subclinical vascular damage in adulthood: Beijing blood pressure cohort.	2018
**Ishida et al (2018)[46]**	Community–based sample of an indigenous population aged 15–75 years	Cross-sectional study	Brachial-ankle	Extracted	132	52	74	Indonesia	2014	Oscillometry	BP-203RPE III		Arterial stiffness, not systolic blood pressure, increases with age in native Papuan populations	2018

Study	Population	Study type	PWV type	Data source	n total	n male	n female	Country	Survey year	Measurement method	Device name	Study name	Paper title	Publication year
**Jang et al (2014)**[47]	Adults aged 20–69 years without history of CVD	Cross-sectional study	Carotid-femoral, Brachial-ankle	Extracted	220	102	118	South Korea	2011	Oscillometry	Colin VP-2000		Determinants of Brachial-Ankle Pulse Wave Velocity and Carotid-Femoral Pulse Wave Velocity in Healthy Koreans	2014
**Jia et al (2017)**[48]	Adults aged 58·1 ± 14·4 years without history of CVD	Cross-sectional study	Brachial-ankle	Extracted	890			China	2014	Oscillometry	Omron VP-1000		Pulse Pressure, Instead of Brachium-Ankle Pulse Wave Velocity, is Associated with Reduced Kidney Function in a Chinese Han Population	2017
**Jiang et al (2020)**[49]	Adults aged 58·86 ± 8·44 years	Cross-sectional study	Brachial-ankle	Extracted	3669			China	2014	Oscillometry	BP-203RPE III		Brachial–ankle pulse wave velocity is independently associated with urine albumin-to-creatinine ratio in a Chinese community-based cohort	2020
**Johansson et al (2014)**[50]	Adults aged 25–74 years (random sample)	Longitudinal cohort study	Carotid-femoral	Extracted	476	227	249	Finland	2007	Tonometry	SphygmoCor	The National Cardiovascular Risk Factor Survey (the Finrisk 2007 study)	Interarm blood pressure difference and target organ damage in the general population	2014
**Jung et al (2013)**[51]	Adults aged 40–89 years without history of CVD	Cross-sectional study	Brachial-ankle	Summary	5532	2116	3416	South Korea	2005-2010	Oscillometry	Colin VP-1000	The Korean Multi-Rural Communities Cohort Study (MRCOHORT)	Cohort Profile: The Korean Genome and Epidemiology Study (KoGES) Consortium.	2013

Study	Population	Study type	PWV type	Data source	n total	n male	n female	Country	Survey year	Measurement method	Device name	Study name	Paper title	Publication year
**Kabutoya et al (2018)**[52]	Adults aged 69·2±11·2 years	Longitudinal cohort study	Brachial-ankle	Extracted	1194			Japan	2015-2018	Oscillometry	Omron	Part of the Coupling Registry	Comparative Assessment of Cutoffs for the Cardio-Ankle Vascular Index and Brachial-Ankle Pulse Wave Velocity in a Nationwide Registry: A Cardiovascular Prognostic Coupling Study	2018
**Kahn et al (2019)**[53]	Children aged 10–12 years, adults from the general population aged 28–68 years	Cross-sectional study nested within national	Carotid-femoral	Individual	3460	1116	2344	Australia	2015-2016	Tonometry	SphygmoCor	Child Health CheckPoint within the Longitudinal Study of Australian Children	Vascular function and stiffness: population epidemiology and concordance in Australian children aged 11-12 years and their parents.	2019
**Kawashima-Kumagai et al (2018)**[54]	Adults aged 52·1±12·8 years	Longitudinal cohort study	Brachial-ankle	Extracted	6719			Japan	2008-2010	Ultrasound	Parks Medical Electronics, Aloha, OR	The Nagahama study	Association of retinal vessel calibers and longitudinal changes in arterial stiffness: the Nagahama study	2018
**Kido et al (2012)**[55]	Adults aged 67·1±8·0 years without history of CVD	Cross-sectional study	Brachial-ankle	Extracted	273			Japan	2006-2010	Oscillometry	Omron	J-SHIPP study	Perceived age of facial features is a significant diagnosis criterion for age-related carotid atherosclerosis in Japanese subjects: J-SHIPP study	2012
**Kim et al (2011)**[56]	Adults aged 20–80 years	Longitudinal cohort study	Brachial-ankle	Extracted	510	191	319	South Korea	2006	Oscillometry	Colin BP-203RPE II	The Korean Sarcopenic Obesity Study	Skeletal muscle mass to visceral fat area ratio is associated with metabolic syndrome and arterial stiffness: The Korean Sarcopenic Obesity Study (KSOS)	2011
**Kim et al (2012)**[57]	Adults aged 52±12 years	Cross-sectional study	Brachial-ankle	Extracted	2800			South Korea	2008-2009	Oscillometry	Omron VP-1000		The Association between Nonalcoholic Fatty Liver Disease, Metabolic Syndrome and Arterial Stiffness in Nondiabetic, Nonhypertensive Individuals	2012

Study	Population	Study type	PWV type	Data source	n total	n male	n female	Country	Survey year	Measurement method	Device name	Study name	Paper title	Publication year
**Kim et al (2014)**[58]	Adults aged 55·7±6·9 years without history of CVD	Longitudinal cohort study	Brachial-ankle	Extracted	1105			South Korea	2009-2010	Oscillometry	Colin VP-2000	Korean Genome and Epidemiology Study	Non-alcoholic fatty liver disease, metabolic syndrome and subclinical cardiovascular changes in the general population	2014
**Kim et al (2015)**[59]	Adults aged 45·8±10·0 years without history of CVD	Cross-sectional study	Brachial-ankle	Extracted	18106			South Korea	2010	Oscillometry	Omron VP 1000	The Kangbuk Samsung Health Study	Sleep Duration, Sleep Quality, and Markers of Subclinical Arterial Disease in Healthy Men and Women	2015
**Kim et al (2017)**[60]	Adults aged 40–78 years without history of CVD	Cross-sectional study	Brachial-ankle	Extracted	684			South Korea	2009-2010	Oscillometry	VP-1000; Dong-A Co. Ltd.,		Relative lower body circumferences are associated with the prevalence of metabolic syndrome and arterial stiffness	2017
**Kim et al (2020)**[61]	Adults aged 18–90 years	Cross-sectional study	Brachial-ankle	Individual	8881	4874	4007	South Korea	2010-2016	Oscillometry	Colin VP-1000		Relationship of Socioeconomic Status to Arterial Stiffness: Comparison Between Medical Aid Beneficiaries and National Health Insurance Beneficiaries	2020
**Kita et al (2003)**[62]	Adults aged 30–75 years	Cross-sectional study	Brachial-ankle	Extracted	126			Japan	1998	Oscillometry	Colin BP-203RPE		Plasma Adrenomedullin Is Closely Correlated with Pulse Wave Velocity in Middle-Aged and Elderly Patients	2003
**Ko et al (2012)**[63]	Adults aged 60·9±10·3 years	Cross-sectional study	Brachial-ankle	Extracted	778	289	489	South Korea	2004-2005	Oscillometry	Colin VP-1000		Relations of Pulse Wave Velocity to Waist Circumference Independent of Hip Circumference	2012
**Kong et al (2017)**[64]	Adults aged 22–88 years without history of CVD	Longitudinal cohort study	Carotid-femoral	Extracted	7154			China	2010	Tonometry	SphygmoCor		Arterial stiffness evaluated by carotid-femoral pulse wave velocity increases the risk of chronic kidney disease in a Chinese population-based cohort	2017

Study	Population	Study type	PWV type	Data source	n total	n male	n female	Country	Survey year	Measurement method	Device name	Study name	Paper title	Publication year
**Kotsis et al (unpublished)**	Children aged 3–17 years, adults aged 18–91 years without history of CVD	Cross-sectional study	Carotid-femoral	Summary	840	382	458	Greece	2010-2020	Tonometry	Complior		Not published	
**Kouda et al (2017)**[65]	Adults aged 69±10 years	Longitudinal cohort study	Brachial-ankle	Extracted	946			Japan	2011-2012	Oscillometry	Colin BP-203RPE II	JPOS Cohort Study	Relative Importance of Central and Peripheral Adiposities on Cardiometabolic Variables in Females: A Japanese Population-Based Study	2017
**Kozakova et al (2017)**[66]	Adolescents aged 13–17 years, adults aged 18–78 years	Cross-sectional study	Carotid-femoral	Individual	653	411	242	Italy	2010-2019*	Tonometry	Complior		Plasma Homocysteine and Cardiovascular Organ Damage in a Population with a High Prevalence of Risk Factors	2017
**Kullo et al (2005)**[67]	Adults aged 32–83 years	Cross-sectional study	Carotid-femoral	Individual	213	114	99	USA	2002-2003	Tonometry	SphygmoCor		C-Reactive Protein Is Related to Arterial Wave Reflection and Stiffness in Asymptomatic Subjects From the Community	2005
**Lamballais et al (2018a)**[45]	Community–based sample of adults aged 60–90+	Longitudinal cohort study	Carotid-femoral	Summary	3445	1465	1980	Netherlands	1997-1999	Tonometry	Complior	Rotterdam Study I, visit 3	Objectives, design and main findings until 2020 from the Rotterdam Study	2018
**Lamballais et al (2018b)**[45]	Community–based sample of adults aged 55–90+	Longitudinal cohort study	Carotid-femoral	Summary	2353	1077	1276	Netherlands	2000-2001	Tonometry	Complior	Rotterdam Study II, visit 1	Objectives, design and main findings until 2020 from the Rotterdam Study	2018
**Lee et al (2017)**[68]	Adults aged 29–84 years without history of CVD	Cross-sectional study	Brachial-ankle	Extracted	302	187	115	South Korea	2015-2016	Oscillometry	Omron VP-1000 plus		Association of Serum 25-hydroxy-vitamin D Concentration and Arterial Stiffness among Korean Adults in Single Center	2017

Study	Population	Study type	PWV type	Data source	n total	n male	n female	Country	Survey year	Measurement method	Device name	Study name	Paper title	Publication year
**Liao et al (2020)**[69]	Children aged 6–15 years	Longitudinal cohort study	Brachial-ankle	Individual	2753	1544	1209	China	1978-2017	Oscillometry	Colin BP-203RPE III	Hanzhong Adolescent Hypertensi on Cohort	Sex differences in impact of long-term burden and trends of body mass index and blood pressure from childhood to adulthood on arterial stiffness in adults: A 30-year cohort study	2020
**Lim et al (2012)**[70]	Adults aged ≥65 years	Cross-sectional study	Carotid-femoral	Extracted	556	237	319	South Korea	2006	Oscillometry	Colin VP-2000	Korean Longitudin al Study on Health and Aging	Subclinical atherosclerosis in a community-based elderly cohort: The Korean Longitudinal Study on Health and Aging	2012
**Lin et al (2009)**[71]	Adults aged ≥40 years without history of CVD	Cross-sectional study	Brachial-ankle	Extracted	1019	486	532	China	2004	Oscillometry	Colin VP-1000	Taichung Communit y Health Study, TCHS	In Addition to Insulin Resistance and Obesity, Brachial-Ankle Pulse Wave Velocity is Strongly Associated with Metabolic Syndrome in Chinese - A Population-Based Study (Taichung Community Health Study, TCHS)	2009
**Lin et al (2017)**[72]	Adults aged 58·2±9·4 years	Cross-sectional study	Brachial-ankle	Extracted	9137			China	2010	Oscillometry	Omron BP203RPEII		High glomerular filtration rate is associated with arterial stiffness in Chinese population	2017
**Liu et al (2018)**[73]	Adults aged 20–94 years	Cross-sectional study	Carotid-femoral	Extracted	979	416	564	China	2010	Tonometry	Complior SP	The Beijing Vascular Disease Patients Evaluation Study	Relationship between Serum Uric Acid and Vascular Function and Structure Markers and Gender Difference in a Real-World Population of China-From Beijing Vascular Disease Patients Evaluation Study (BEST) Study	2018
**Logan et al (2013)**[74]	Adults aged 21–60 years without history of CVD	Cross-sectional study	Carotid-femoral	Individual	101	40	61	South Korea	Missin g	Tonometry	SphygmoCor		Pulse Wave Velocity in Korean American Men and	2013

Study	Population	Study type	PWV type	Data source	n total	n male	n female	Country	Survey year	Measurement method	Device name	Study name	Paper title	Publication year
**Lopez-Sublet et al (2019)[75]**	Adults aged 45±14 years	Longitudinal cohort study	Carotid-femoral	Extracted	1334			France	2011-2016	Tonometry	Complior	The STANISLAS Cohort	Nondipping Pattern and Cardiovascular and Renal Damage in a Population-Based Study (The STANISLAS Cohort Study)	2019
**Lu et al (2020)[76]**	Adolescents aged 14–17 years, adults aged 18–96 years	2 cross-sectional studies and 1 longitudinal cohort study	Brachial-ankle	Individual	87612	56686	30926	China	2004-2016	Oscillometry	Omron BP-203 RPE III		Trajectories of Age-Related Arterial Stiffness in Chinese Men and Women	2020
**Maddock et al (2018)[77]**	Adults aged 60–64 years	Longitudinal cohort study	Carotid-femoral	Summary	1279	597	682	UK	2006-2010	Oscillometry	Vicorder	MRC National Survey for Health and Development	Adherence to a Dietary Approaches to Stop Hypertension (DASH)-type diet over the life course and associated vascular function: a study based on the MRC 1946 British birth cohort	2018
**Magalhães et al (2013)[78]**	Adults aged 22–72 years without history of CVD	Cross-sectional study	Carotid-femoral	Individual	542	273	269	Angola	2009-2010	Tonometry	Complior		Age- and gender-specific reference values of pulse wave velocity for African adults: preliminary results	2013
**Magalhães et al (2019)[79]**	Women aged 45–65 years without history of CVD	Cross-sectional study	Carotid-femoral	Extracted	277			Brazil	2011-2012	Tonometry	Complior		Migraine and Markers of Carotid Atherosclerosis in Middle-Aged Women: A Cross-Sectional Study	2019
**Maimaitiaili et al (2020)[80]**	Adults aged 71·1±6·0 years	Longitudinal cohort study	Carotid-femoral, Brachial-ankle	Extracted	6726	2934	3792	China	2017	Tonometry, Oscillometry	SphygmoCor, Omron VP1000	The Northern Shanghai Study	Relationship Between Vascular Aging and Left Ventricular Concentric Geometry in Community-Dwelling Elderly: The Northern Shanghai Study	2020

Study	Population	Study type	PWV type	Data source	n total	n male	n female	Country	Survey year	Measurement method	Device name	Study name	Paper title	Publication year
**Maldonado et al (2011)**[81]	Adults aged 18–91 years	Longitudinal cohort study	Carotid-femoral	Individual	2199	1290	909	Portugal	2005-2010	Tonometry	Complior	EDIVA - Estudo de DIstensibilidade Vascular (Study of Arterial Stiffness)	Aortic pulse wave velocity and HeartSCORE: Improving cardiovascular risk stratifi cation. A sub-analysis of the EDIVA (Estudo de DIstensibilidade VAscular) project	2011
**Matsumoto et al (2020)**[82]	Adults aged 49·5±15·1 years without history of CVD	Cross-sectional study	Brachial-ankle	Extracted	1344	535	809	Japan	2018	Oscillometry	Omron Colin Form PWV/ABI		Skin Carotenoid Level as an Alternative Marker of Serum Total Carotenoid Concentration and Vegetable Intake Correlates with Biomarkers of Circulatory Diseases and Metabolic Syndrome	2020
**Matsuoka et al (2005)**[83]	Adults aged 79·0±4·75 years	Longitudinal cohort study	Brachial-ankle	Extracted	298			Japan	2000	Oscillometry	Colin	The Longevity and Aging in Hokkaido County (LILAC) study	Arterial stiffness independently predicts cardiovascular events in an elderly community - Longitudinal Investigation for the Longevity and Aging in Hokkaido County (LILAC) study	2005
**McEniery et al (2010)**[84]	Community–based sample of adults aged 45–59 years	Longitudinal cohort study	Carotid-femoral	Extracted	825			UK	2002-2004	Tonometry	SphygmoCor	The Caerphilly Prospective Study	An Analysis of Prospective Risk Factors for Aortic Stiffness in Men 20-Year Follow-Up From the Caerphilly Prospective Study	2010
**Metsämarttila et al (2018)**[85]	Adults aged 68–69 years	Cross-sectional study	Carotid-femoral	Individual	570	232	338	Finland	2013-2015	Tonometry	SphygmoCor	1945 Oulu Birth Cohort	Effect of physical activity on pulse wave velocity in elderly subjects with normal glucose, prediabetes or Type 2 Diabetes.	2018

Study	Population	Study type	PWV type	Data source	n total	n male	n female	Country	Survey year	Measurement method	Device name	Study name	Paper title	Publication year
**Michener et al (2015)[86]**	Community–based sample of adults aged 69–96 years	Longitudinal cohort study	Carotid-femoral	Extracted	940			Iceland	2002	Tonometry	SPT-301, Millar	Age Gene/Environment Susceptibility-Reykjavik Study (AGES-RS)	Aortic stiffness and kidney disease in an elderly population	2015
**Mikumo et al (2009)[87]**	Postmenopausal women aged 57·9±8·3 years	Cross-sectional study	Brachial-ankle	Extracted	142			Japan	2004-2005	Oscillometry	Nippon Colin		Association between lumber bone mineral density and vascular stiffness as assessed by pulse wave velocity in postmenopausal women	2009
**Miljkovic et al (2013)[88]**	Adults aged 80–90+	Longitudinal cohort study	Carotid-femoral	Extracted	1071			France	2006-2008	Tonometry	PulsePen	The PARTAGE Study	Correlation Between Peripheral Blood Pressure and Pulse-Wave Velocity Values in the Institutionalized Elderly Persons 80 Years of Age and Older: The PARTAGE Study	2013
**Miyaki et al (2006)[89]**	Adults aged 20–64 years	Cross-sectional study	Brachial-ankle	Extracted	377			Japan	2001	Oscillometry	Nippon Colin VP-2000/1000		Two New Criteria of the Metabolic Syndrome:Prevalence and the Association with Branchial-Ankle Pulse Wave Velocity in Japanese Male Workers	2006
**Mokwatsi et al (2017)[90]**	Black and white boys aged 6–8 years	Cross-sectional study	Carotid-femoral	Summary	81	81		South Africa	2015	Tonometry	Complior	The Arterial Stiffness in Offspring Study (ASOS)	Ethnic differences regarding arterial stiffness of 6-8-year-old black and white boys	2017
**Muhammad et al (2017)[91]**	Community–based sample of adults aged 61–85 years	Longitudinal cohort study	Carotid-femoral	Summary	3056	1208	1848	Sweden	2007-2012	Tonometry	SphygmoCor	Malmö diet and cancer study - Cardiovascular Cohort (MDCS_CV).	Acute phase proteins as prospective risk markers for arterial stiffness: The Malmo¨ Diet and Cancer cohort	2017

Study	Population	Study type	PWV type	Data source	n total	n male	n female	Country	Survey year	Measurement method	Device name	Study name	Paper title	Publication year
**Nakagomi et al (2019)[92]**	Adults aged 25–55 years without history of CVD	Cross-sectional study	Brachial-ankle	Summary	3274	2024	1250	Japan	2017-2019	Oscillometry	Omron Colin		Sex difference in the association between surrogate markers of insulin resistance and arterial stiffness	2019
**Nakamura et al (2016)[93]**	Adults aged 30–70 years	Longitudinal cohort study	Brachial-ankle	Summary	876	297	579	Japan	2003-2013	Oscillometry	Colin BP-203RPE II	The Mikkabi Study	Serum β-cryptoxanthin and β-carotene derived from Satsuma mandarin and brachial-ankle pulse wave velocity: the Mikkabi cohort study	2016
**Nakanishi et al (2005)[94]**	Adults aged 40–69 years without history of CVD	Cross-sectional study	Brachial-ankle	Extracted	996	373	622	Japan	2003	Oscillometry	Nihon Colin	The Minoh Study	Brachial-Ankle Pulse Wave Velocity and Metabolic Syndrome in a Japanese Population: The Minoh Study	2005
**Niboshi et al (2006)[95]**	Children aged 9–17 years	Cross-sectional study	Brachial-ankle	Extracted	970	500	470	Japan	Missin g	Oscillometry	Colin formPWV/ABI		Characteristics of brachial–ankle pulse wave velocity in Japanese children	2006
**Ninomiya et al (2013)[96]**	Adults aged 40–94 years without history of CVD	Longitudinal cohort study	Brachial-ankle	Summary	2916	1246	1670	Japan	2002-2009	Oscillometry	BP-203PRE II form PWV/ABI	The Hisayama Study	Brachial-ankle pulse wave velocity predicts the development of cardiovascular disease in a general Japanese population: the Hisayama Study	2013
**Oikonomou et al (2020)[97]**	Adults from the general population aged 40–99 years	Cross-sectional study	Carotid-femoral	Extracted	1510			Greece	2015-2017	Tonometry	SphygmoCor	The Corinthia study		2020
**Oren et al (2003)[98]**	Adults aged 27–30 years	Cross-sectional study	Carotid-femoral	Extracted	524	240	284	Netherlan ds	1999-2000	Tonometry	SphygmoCor	Atheroscler osis Risk in Young Adults study	Aortic stiffness and carotid intima-media thickness: two independent markers of subclinical vascular damage in young adults?	2003

Study	Population	Study type	PWV type	Data source	n total	n male	n female	Country	Survey year	Measurement method	Device name	Study name	Paper title	Publication year
**Oughton et al (2015)[99]**	Women aged 40–79 years	Longitudinal cohort study	Carotid-femoral	Extracted	159			Australia	2005	Tonometry	SphygmoCor	The Longitudinal Assessment of Ageing in Women (LAW) study	Carotid ultrasound pulsatility indices and cardiovascular risk in Australian women	2015
**Paini et al (2020)[100]**	Adults aged 63±13 years	Cross-sectional study	Carotid-femoral	Extracted	284			Italy	2015	Tonometry	Complior		Relationship between arterial stiffness and unattended or attended blood pressure values	2020
**Pan et al (2018)[101]**	Adults aged 59·3±6·9 years	Cross-sectional study	Brachial-ankle	Extracted	1528			China	2003-2008	Oscillometry	Colin BP-203RPE	Guangzhou biobank cohort study-CVD	Relationship between pulmonary function and peripheral vascular function in older Chinese: Guangzhou biobank cohort study-CVD	2018
**Pan et al (2019)[102]**	Adults aged 85·7±5·3 years	Cross-sectional study	Brachial-ankle	Extracted	155			China	2018	Oscillometry	Colin-Omron BP-203RPE III		Carotid plague formation is associated with ankle–brachial index in elderly people	2019
**Park et al (2012)[103]**	Postmenopausal women aged 60·63±7·31 years	Cross-sectional study	Carotid-femoral	Extracted	841			South Korea	2007	Tonometry	Hanbyul PP-1000		Relationships between serum uric acid, adiponectin and arterial stiffness in postmenopausal women	2012
**Pivin et al (2015)[104]**	Adults from the general population aged 46·5±17·2 years	Cross-sectional study	Carotid-femoral	Extracted	1002			Switzerland	2009-2013	Tonometry	SphygmoCor	SKIPOGH	Inactive Matrix Gla-Protein Is Associated With Arterial Stiffness in an Adult Population–Based Study	2015
**Podolec et al (2019)[105]**	Adults aged 58·5±6·5 years	Cross-sectional study	Carotid-femoral	Extracted	720			Poland	NA	Tonometry	Complior		Association between carotid-femoral pulse wave velocity and overall cardiovascular risk score assessed by the SCORE system in urban Polish population	2019

Study	Population	Study type	PWV type	Data source	n total	n male	n female	Country	Survey year	Measurement method	Device name	Study name	Paper title	Publication year
**Poon et al (2020)[106]**	Adults aged 45 and older	Longitudinal cohort study	Carotid-femoral	Summary	4563	1853	2710	USA	2011-2013	Oscillometry	Omron VP-1000 Plus	Atherosclerosis Risk of Communities Study (ARIC)	The Atherosclerosis Risk in Communities (ARIC) Study: design and objectives. The ARIC investigators.	2020
**The PORT-VASPh Study**	Children aged 4–18 years	Longitudinal cohort study	Carotid-femoral	Individual	1924	1177	747	Portugal	2017-2021	Tonometry	Complior	Portuguese Vascular Phenotype in Children and Adolescents (PORT-VASPh) Cohort	Not published	
**The Prospective Urban and Rural Epidemiology (PURE) study**	Healthy black adults aged 35–70 years	Longitudinal cohort study	Carotid-femoral	Individual	793	248	545	South Africa	2015	Tonometry	SphygmoCor	PURE	Not published	
**Recio-Rodriguez et al (2014)[107]**	Adults aged 20–80 years	Cross-sectional study	Carotid-femoral	Extracted	265			Spain	2011-2012	Tonometry	SphygmoCor	The EVIDENT study	Association between fat amount of dairy products with pulse wave velocity and carotid intima-media thickness in adults	2014
**Reusz et al (2010)[108]**	Children aged 6 to 19 years	Cross-sectional study	Carotid-femoral	Individual	1002	493	509	Hungary, Algeria, Italy	2006-2009	Tonometry	PulsePen device		Reference Values of Pulse Wave Velocity in Healthy Children and Teenagers	2010
**Rhee et al (2018)[109]**	Adults aged 58·1±12·2 years without history of CVD	Cross-sectional study	Brachial-ankle	Extracted	539	270	269	South Korea	2012-2013	Oscillometry	Colin VP-1000		Gender difference in the association between brachial-ankle pulse wave velocity and cardiovascular risk scores	2018
**Rojek et al (2018)[110]**	Adults aged 40–65 years	Cross-sectional study	Carotid-femoral	Individual	200	136	64	Poland	2015-2016	Tonometry	SphygmoCor		The relation between blood pressure components and left atrial volume in the context of left ventricular mass index	2018

Study	Population	Study type	PWV type	Data source	n total	n male	n female	Country	Survey year	Measurement method	Device name	Study name	Paper title	Publication year
**The RPS Birth Consortium (a)**	Adults aged 18–19 years	Longitudinal cohort study	Carotid-femoral	Individual	2312	1110	1202	Brazil	2016	Tonometry	SphygmoCor	RPS Birth Consortium	Not published	
**RPS Birth Consortium (b)**	Adults aged 37–39 years	Longitudinal cohort study	Carotid-femoral	Individual	1675	813	862	Brazil	2016-2017	Tonometry	SphygmoCor	RPS Birth Consortium	Not published	
**Saijo et al (2005)[111]**	Adults aged 35–61 years without CVD	Cross-sectional study	Brachial-ankle	Summary	4266	3412	854	Japan	2003-2004	Oscillometry	Colin BP-203RPEII		Relationship of Helicobacter pylori Infection to Arterial Stiffness in Japanese Subjects	2005
**Saji et al (2012)[112]**	Adults aged 69±9 years without history of CVD	Cross-sectional study	Brachial-ankle	Extracted	240			Japan	2003-2010	Oscillometry	Omron		Association between Silent Brain Infarct and Arterial Stiffness Indicated by Brachial-ankle Pulse Wave Velocity	2012
**Samargandy et al (2020)[113]**	Women aged 42–52 years	Longitudinal cohort study	Carotid-femoral	Extracted	339			USA	1996-1997	Oscillometry	Vasera-1500	The SWAN Heart Study	Arterial Stiffness Accelerates Within 1 Year of the Final Menstrual Period The SWAN Heart Study	2020
**Sang et al (2020)[114]**	Healthy adults aged 66·5±6·8 years	Cross-sectional study	Brachial-ankle	Extracted	794			China	2014-2019	Oscillometry	Omron BP-203RPEIII		Determinants of Brachial-Ankle Pulse Wave Velocity and Vascular Aging in Healthy Older Subjects	2020
**Scuteri et al (2012)[115]**	Adolescents aged 14–17 years, adults from the general population aged 18–102 years	Cross-sectional study	Carotid-femoral	Extracted	6148			Italy	2001-2004	Ultrasound	Parks Model 810A Doppler probes	The SardiNIA Study	Associations of large artery structure and function with adiposity effects of age, gender and hypertension: The SardiNIA Study	2012
**Segers et al (2007)[116]**	Adults aged 35–44 years	Longitudinal cohort study	Carotid-femoral	Individual	2444	1176	1268	Belgium	NA	Tonometry	Vivid7, GE	Asklepios study	Noninvasive (Input) Impedance, Pulse Wave Velocity, and Wave Reflection in Healthy Middle-Aged Men and Women	2007

Study	Population	Study type	PWV type	Data source	n total	n male	n female	Country	Survey year	Measurement method	Device name	Study name	Paper title	Publication year
**Sforza et al (2019)**[117]	Adults aged 75·3±0·7 years without history of CVD	Longitudinal cohort study	Carotid-femoral	Extracted	101	42	59	France	1998-2010	Tonometry	Complior	PROOF cohort study	Arterial stiffness alteration and obstructive sleep apnea in an elderly cohort free of cardiovascular event history: the PROOF cohort study	2019
**Sheng et al (2014)**[118]	Community–based sample of adults aged 68·1±7·3 years	Longitudinal cohort study	Brachial-ankle	Extracted	3876			China	2006-2011	Oscillometry	Omron VP-1000		Brachial-Ankle Pulse Wave Velocity as a Predictor of Mortality in Elderly Chinese	2014
**Shokawa et al (2005)**[119]	Adults aged 40–79 years	Longitudinal cohort study	Carotid-femoral	Extracted	492			USA	1984	Tonometry	Fukuda Denshi MCG400	The Hawaii-Los Angeles-Hiroshima Study	Pulse Wave Velovity Predicts Cardiovascular Mortality Findings From the Hawaii-Los Angeles-Hiroshima Study	2005
**Silva et al (2016)**[120]	Children aged 7–12 years	Cross-sectional study	Carotid-femoral	Individual	195	76	119	Angola	2012-2013	Tonometry	Complior		Predictors and Reference Values of Pulse Wave Velocity in Prepubertal Angolan Children	2016
**Sonoda et al (2012)**[121]	Adults aged 61·3±8·5 years	Cross-sectional study	Brachial-ankle	Extracted	911			Japan	2009	Oscillometry	Omron Colin BP-203RPE II		Factors associated with brachial-ankle pulse wave velocity in the general population	2012
**Sougawa et al (2020)**[122]	Adolescents aged 12–18 years	Cross-sectional study	Brachial-ankle	Extracted	4524	2272	2252	Japan	2002-2018	Oscillometry	Omron BP-203RPE II/III		Brachial-ankle pulse wave velocity in healthy Japanese adolescents: reference values for the assessment of arterial stiffness and cardiovascular risk profiles	2019

Study	Population	Study type	PWV type	Data source	n total	n male	n female	Country	Survey year	Measurement method	Device name	Study name	Paper title	Publication year
**Stamatelopoulos [123] et al (2020)**	Postmenopausal women aged 33–82 years without history of CVD	Cross-sectional study	Carotid-femoral	Individual	878		878	Greece	2006-2018	Tonometry	Complior		Physical activity is associated with lower arterial stiffness in normal-weight postmenopausal women (the one we have in our studies folder: The TyG Index as a Marker of Subclinical Atherosclerosis and Arterial Stiffness in Lean and Overweight Postmenopausal Women)	2020 (the study in our folder: 2018)
**Strazhesko et al (2017)[124]**	Adults aged 23–91 years without history of CVD	Cross-sectional study	Carotid-femoral	Individual	279	96	183	Russia	2012-2013	Tonometry	SphygmoCor		Growth Hormone, Insulin-Like Growth Factor-1, Insulin Resistance, and Leukocyte Telomere Length as Determinants of Arterial Aging in Subjects Free of Cardiovascular Diseases	2017
**Su et al (2014)[125]**	Adults aged 60·8±13·7 years	Cross-sectional study	Brachial-ankle	Extracted	1059			China	2010-2011	Oscillometry	Colin VP1000		Association of Interankle Systolic Blood Pressure Difference With Peripheral Vascular Disease and Left Ventricular Mass Index	2014
**Takahashi et al (2021)[126]**	Adults aged 30–65 years without history of CVD	Longitudinal cohort study	Brachial-ankle	Individual	2840	2418	422	Japan	2013-2021	Oscillometry	Form/ABI, Omron Healthcare Co., Ltd., Kyoto, Japan		Association of pulse wave velocity and pressure wave reflection with the ankle-brachial pressure index in Japanese men not suffering from peripheral artery disease	2021

Study	Population	Study type	PWV type	Data source	n total	n male	n female	Country	Survey year	Measurement method	Device name	Study name	Paper title	Publication year
**Takashima et al (2014)[127]**	Adults aged 58·9±13·0 years without history of CVD	Longitudinal cohort study	Brachial-ankle	Extracted	4164			Japan	2002-2009	Oscillometry	Omron BP-203RPE II Form I	The Takashima Study	The relationship of brachial-ankle pulse wave velocity to future cardiovascular disease events in the general Japanese population: the Takashima Study	2014
**Tang et al (2020)[128]**	Adults aged 48·3±14·6 years	Cross-sectional study	Brachial-ankle	Extracted	578			China	2017-2018	Oscillometry	Omron BP-203RPEIII VP-1000		Relationship between body mass index and arterial stiffness in a health assessment Chinese population	2020
**Temmar et al (2013)[129]**	Adults aged 81·1±4·9 years	Longitudinal cohort study	Carotid-femoral	Extracted	321			Algeria	Missing	Tonometry	Complior	The ERASAG (Evolution de la Rigidite´ Arte´rielle Sujets Age´s Ghardaia) study (Algerian)	ElderlyAlgerian women lose their sex-advantage in terms of arterial stiffness and cardiovascular profile	2013
**Tomiyama et al (2003)[130]**	Adults aged 25–87 years without history of CVD	Cross-sectional study	Brachial-ankle	Extracted	12517	8226	4290	Japan	1998	Oscillometry	Colin Form		Influences of age and gender on results of noninvasive brachial-ankle pulse wave velocity measurement-a survey of 12 517 subjects	2003
**Topel et al (2018)[131]**	Adults aged 40–78 years	Cross-sectional study	Carotid-femoral	Individual	1119	442	677	USA	2005-2012	Tonometry	SphygmoCor	META-Health and CHDWB	Comparisons of the Framingham and Pooled Cohort Equation Risk Scores for Detecting Subclinical Vascular Disease in Blacks Versus Whites	2018
**Torigoe et al (2020)[132]**	Children aged 0–18 years	Cross-sectional study	Carotid-femoral	Summary	315	158	157	Canada	2008-2016	Ultrasound	Vivid 7, GE		New Comprehensive Reference Values for Arterial Vascular Parameters in Children.	2020

Study	Population	Study type	PWV type	Data source	n total	n male	n female	Country	Survey year	Measurement method	Device name	Study name	Paper title	Publication year
**Tsao et al (2018)[133]**	Adults aged 66±11 years	Longitudinal cohort study	Carotid-femoral	Extracted	1322	451	872	USA	2011-2016	Tonometry	Custom tonometer, Cardiovascular Engineering	JHS	Clinical Correlates of Aortic Stiffness and Wave Amplitude in Black Men and Women in the Community	2018
**Tsuchikura et al (2010)[134]**	Adults aged 60 (53–67([median (IQR)] years	Cross-sectional study	Brachial-ankle	Extracted	2806			Japan	2000-2009	Oscillometry	Colin BP-203RPE		Brachial-ankle Pulse Wave Velocity as an Index of Central Arterial Stiffness	2010
**Uetani et al (2012)[135]**	Adults aged 66·2±8·9 years without history of CVD	Cross-sectional study	Brachial-ankle	Summary	1340			Japan	2006-2011	Oscillometry	Omron BP-203RPEII		Postprandial hypertension, an overlooked risk marker for arteriosclerosis	2012
**Urbina et al (2012)[136]**	Adolescents aged 15–17 years, adults aged 18–28 years	Cross-sectional study	Carotid-femoral	Summary	1362	596	766	USA	Missing	Tonometry	SphygmoCor		Insulin resistance and arterial stiffness in healthy adolescents and young adults	2012
**Van den Munckhof et al (2017)[137]**	Adults aged 50–70 years	Cross-sectional study	Carotid-femoral	Summary	1473	725	748	Netherlands	2005-2008	Tonometry	SphygmoCor		Sex differences in fat distribution influence the association between BMI and arterial stiffness	2017
**Van Varik et al (2017)[138]**	Adults aged 62±11 years	Longitudinal cohort study	Carotid-femoral	Extracted	587			Netherlands	2012	Tonometry	Complior	The HIPPOCRATES project	Arterial stiffness and decline of renal function in a primary care population	2017
**Waldstein et al (2008)[139]**	Adults aged 54±17 years	Longitudinal cohort study	Carotid-femoral	Extracted	579			USA	2002	Ultrasound	Doppler probes Model 810A, Parks	The Baltimore Longitudinal Study of Aging (BLSA)	Pulse Pressure and Pulse Wave Velocity Are Related to Cognitive Decline in the Baltimore Longitudinal Study of Aging	2008
**Wang et al (2009)[140]**	Adults aged 30–79 years	Longitudinal cohort study	Carotid-femoral	Extracted	1271	674	598	China	2004	Ultrasound	Doppler Parks model 802		Central or peripheral systolic or pulse pressure. Which best relates to target organs and future mortality?	2009
**Wang et al (2019)[141]**	Adults aged 57·8±9·4 years	Longitudinal cohort study	Brachial-ankle	Extracted	8396			China	2010	Oscillometry	Colin VP-1000 BP203RPE II		Ideal Cardiovascular Health is Inversely Associated with Subclinical Atherosclerosis: A Prospective Analysis	2019

Study	Population	Study type	PWV type	Data source	n total	n male	n female	Country	Survey year	Measurement method	Device name	Study name	Paper title	Publication year
**Watanabe et al (2015)[142]**	Adults aged 20–25 years	Cross-sectional study	Brachial-ankle	Extracted	641			Japan	2002-2013	Oscillometry	Form PWV/ABI		Ankle-Brachial Index, Toe-Brachial Index, and Pulse Volume Recording in Healthy Young Adults	2015
**Wendell et al (2017)[143]**	Adults aged 30–64 years without history of CVD	Longitudinal cohort study	Carotid-femoral	Extracted	2270			USA	2004-2009	Ultrasound	Doppler Model 810A, Parks	The HANDLS study	Distributions of subclinical cardiovascular disease in a socioeconomically and racially diverse sample	2017
**Wijnands et al (2015)[144]**	Adults aged 40–75 years without history of CVD	Cross-sectional study	Carotid-femoral	Extracted	615			Netherlands	2010-2012	Tonometry	SphygmoCor	The Maastricht Study	Association between serum uric acid, aortic, carotid and femoral stiffness among adults aged 40^75 years without and with type 2 diabetes mellitus:The Maastricht Study	2015
**Wohlfahrt et al (2014)[145]**	Adults from the general population aged 25–64 years	Cross-sectional study	Carotid-femoral	Extracted	1031			Czech Republic	2006-2009	Tonometry	SphygmoCor	The Czech MONICA and post-MONICA study	Relationship between Measures of Central and General Adiposity with Aortic Stiffness in the General Population	2014
**Woodiwiss et al (2017)[146]**	Adults aged 44·1±18·1 years	Cross-sectional study	Carotid-femoral	Extracted	771			South Africa	Missing	Tonometry	SphygmoCor		Association of Blood Pressure Variability Ratio With Glomerular Filtration Rate Independent of Blood Pressure and Pulse Wave Velocity	2017
**Wu et al (2019)[147]**	Adults aged 50·1±0·1 years	Longitudinal cohort study	Brachial-ankle	Extracted	20310			China	2010-2015	Oscillometry	Omron BP-203RPEIII	Kailuan cohort study	Effect of brachial-ankle pulse wave velocity combined with blood pressure on cardio-cerebrovascular events	2019
**Xie et al (2013)[148]**	Adults aged 50·8±12·6 years	Cross-sectional study	Brachial-ankle	Extracted	13899			China	2007-2010	Oscillometry	Colin VP1000	The Cardiovascular Risk Survey (CRS) study	Decreased estimated glomerular filtration rate (eGFR) is not an independent risk factor of arterial stiffness in Chinese women	2013

Study	Population	Study type	PWV type	Data source	n total	n male	n female	Country	Survey year	Measurement method	Device name	Study name	Paper title	Publication year
**Xuan et al (2019)[149]**	Adults aged 83·2±12·8 years	Cross-sectional study	Brachial-ankle	Extracted	129	66	62	China	2014	Oscillometry	Omron BP-203RPEIII		Osteoporosis is inversely associated with arterial stiffness in the elderly: An investigation using the Osteoporosis Self-assessment Tool for Asians index in an elderly Chinese cohort	2019
**Yang et al (2014)[150]**	Adults aged 59·9±9·1 years	Cross-sectional study	Brachial-ankle	Extracted	5158			China	2011	Oscillometry	Colin VP-1000		Visceral Adiposity Index May Be a Surrogate Marker for the Assessment of the Effects of Obesity on Arterial Stiffness	2014
**Framingham Heart Study (FHS) Cohorts: Mitchell (2010) and Zachariah (2018)[151,152]**	Adults aged 19–91 years	Longitudinal cohort study	Carotid-femoral	Individual	6387	2943	3444	USA	1948-2005	Tonometry		FHS Third Generation, OMNI 2, and New Offspring Cohorts	Metabolic Predictors of Change in Vascular Function: Prospective Associations from a Community-Based Cohort	2018
**Zaniqueli et al (2017)[153]**	Children aged 6–18 years	Cross-sectional study	Carotid-femoral	Individual	1093	583	510	Brazil	2014-2016	Tonometry	Complior, SP; Artech Medical, Pantin, France		Ethnicity and arterial stiffness in children and adolescents from a Brazilian population	2017
**Zeng et al (2017)[154]**	Adults aged 46·8±7·8 years	Cross-sectional study	Brachial-ankle	Extracted	11014	6187	4827	China	2012-2014	Tonometry	SphygmoCor		Serum glycated albumin, glycated hemoglobin, and arterial stiffness in a general Chinese population	2017
**Zhang et al (2017)[155]**	Adults aged 18–80 years without history of CVD	Cross-sectional study	Brachial-ankle	Extracted	10197	8415	1782	China	2012-2013	Oscillometry	Arteriograph VP-1000 BP-203RPEII		Comparison of the ability to identify arterial stiffness between two new anthropometric indices and classical obesity indices in Chinese adults	2017

Study	Population	Study type	PWV type	Data source	n total	n male	n female	Country	Survey year	Measurement method	Device name	Study name	Paper title	Publication year
**Zhang et al (2020)[156]**	Adults aged 72±5 years without history of CVD	Cross-sectional study	Brachial-ankle	Extracted	1046	448	598	Japan	2016-2018	Oscillometry	Colin BP-203RPE II/III	The Wakayama Study	Muscle mass reduction, low muscle strength, and their combination are associated with arterial stiffness in community-dwelling elderly population: the Wakayama Study	2020
**Zhao et al (2014)[157]**	Community–based sample of adults aged 50–90 years	Cross-sectional study	Brachial-ankle	Extracted	1133	430	703	China	2007-2008	Oscillometry	Colin BP-203 RPE-II		Association of lipid profiles and the ratios with arterial stiffness in middle-aged and elderly Chinese	2014
**Zheng et al (2015)[158]**	Adults aged 18–85 years	Cross-sectional study	Brachial-ankle	Extracted	20748			China	2009-2012	Oscillometry	Kingrich Arteriosclerosis Detector VBP-9		Relationship between Sum of the Four Limbs' Pulse Pressure and Brachial-Ankle Pulse Wave Velocity and Atherosclerosis Risk Factors in Chinese Adults	2015
**Zhou et al (2017)[159]**	Females aged 40–60 years without history of CVD	Cross-sectional study	Brachial-ankle	Extracted	1647			China	2016	Oscillometry	Omron BP-203RPE III		Sleep disorder, an independent risk associated with arterial stiffness in menopause	2017
**Zureik et al (2002)[160]**	Adults aged 58·2±10·8 years	Longitudinal cohort study	Carotid-femoral	Extracted	564	384	180	France	1998-1999	Tonometry	Complior		Carotid plaques, but not common carotid intima–media thickness, are independently associated with aortic stiffness	2002
**Zureik et al (2006)[161]**	Adults aged 59·7 ±4·7 years	Longitudinal cohort study	Carotid-femoral	Extracted	1117			France	2002	Tonometry	Complior	SUVIMAX Vascular Study	Parental Longevity, Carotid Atherosclerosis, and Aortic Arterial Stiffness in Adult Offspring	2006

Supplemental Table 2: Description of studies included.

Published studies are identified by citation and first author of the relevant publication, unpublished studies (n=8) by the study name or principal investigator.

n indicates the number of data points available for this meta-analysis, which may differ from the number reported by individual studies because (1) some studies measured both baPWV and cfPWV in the same subjects, (2) some studies provided more data than were used for the original paper that applied additional exclusion criteria, and (3) 0.1% was trimmed from the extremes of the individual participant data distribution to remove implausible values.

Total n may not be the sum of n for males and females separately because PWV was not always reported in the sexes individually.

Study	Criteria for inclusion clearly defined	Detailed description of study subjects and setting	Measurement of exposure in a valid and reliable way	Objective standard criteria for measurement of condition	Identification of confounding factors	Statement of strategies to deal w confounding factors	Measurement of outcomes in valid and reliable way	Use of appropriate statistical analysis	Overall appraisal	Quality rating
**Achimastos et al (2007)**	Yes	Yes	Yes	N/A	Yes	Yes	Yes	Yes	Yes	High quality
**Ai et al (2011)**	Yes	Yes	Yes	N/A	Yes	Yes	Yes	Yes	Yes	High quality
**Araghi et al (2020)**	Yes	Yes	Yes	N/A	Yes	Yes	Yes	Yes	Yes	High quality
**Avramovski et al (2016)**	Yes	Yes	Yes	N/A	Yes	Yes	Yes	Yes	Yes	High quality
**Baier et al (2018)**	Yes	Yes	Yes	N/A	Yes	Yes	Yes	Yes	Yes	High quality
**Baldo et al (2017)**	Yes	Yes	Yes	N/A	Yes	Yes	Yes	Yes	Yes	High quality
**Benetos et al (2009)**	Yes	Yes	Yes	N/A	Yes	Yes	Yes	Yes	Yes	High quality
**Bérard et al (2013)**	Yes	Yes	Yes	N/A	Yes	Yes	Yes	Yes	Yes	High quality
**Bia et al (2021)**	Yes	Yes	Yes	N/A	Yes	Yes	Yes	Yes	Yes	High quality
**Bian et al (2012)**	Yes	Yes	Yes	N/A	Yes	Yes	Yes	Yes	Yes	High quality
**Botha et al (2021)**	Yes	Yes	Yes	N/A	Yes	Yes	Yes	Yes	Yes	High quality
**Cecelja et al (2011)**	Yes	Yes	Yes	N/A	Yes	Yes	Yes	Yes	Yes	High quality
**Cecelja et al (2020)**	Yes	Yes	Yes	N/A	Yes	Yes	Yes	Yes	Yes	High quality
**Ceponiene et al (2015)**	Yes	Yes	Yes	N/A	Yes	Yes	Yes	Yes	Yes	High quality
**Chen et al (2018)**	Yes	Yes	Yes	N/A	Yes	Yes	Yes	Yes	Yes	High quality
**Cho et al (2020)**	Yes	Yes	Yes	N/A	Yes	Yes	Yes	Yes	Yes	High quality
**Choi et al (2010)**	Yes	Yes	Yes	N/A	Yes	Yes	Yes	Yes	Yes	High quality
**Choi et al (2017)**	Yes	Yes	Yes	N/A	Yes	Yes	Yes	Yes	Yes	High quality
**Chou et al (2015)**	Yes	Yes	Yes	N/A	Yes	Yes	Yes	Yes	Yes	High quality
**Chuang et al (2005)**	Yes	Yes	Yes	N/A	Yes	Yes	No	Yes	Yes	Other quality
**Cicero et al (2020)**	Yes	Yes	Yes	N/A	Yes	Yes	Yes	Yes	Yes	High quality
**Cruz et al (2020)**	Yes	Yes	Yes	N/A	Yes	Yes	Yes	Yes	Yes	High quality
**Cunha et al (2015)**	Yes	Yes	Yes	N/A	Yes	Yes	Yes	Yes	Yes	High quality
**Da Silva et al (2016)**	Yes	Yes	Yes	N/A	Yes	Yes	Yes	Yes	Yes	High quality
**De Mendonça et al (2018)**	Yes	Yes	Yes	N/A	Yes	Yes	Yes	Yes	Yes	High quality
**De Oliveira Alvim et al (2015)**	Yes	Yes	Yes	N/A	Yes	Yes	Yes	Yes	Yes	High quality
**Del Giorno et al (2021)**	Yes	Yes	Yes	N/A	Yes	Yes	Yes	Yes	Yes	High quality
**Diaz et al (2018)**	Yes	Yes	Yes	N/A	Yes	Yes	Yes	Yes	Yes	High quality
**Ellins et al (2017)**	Unclear	Yes	Yes	N/A	Yes	Yes	Yes	Yes	Yes	Other quality

Study	Criteria for inclusion clearly defined	Detailed description of study subjects and setting	Measurement of exposure in a valid and reliable way	Objective standard criteria for measurement of condition	Identification of confounding factors	Statement of strategies to deal w confounding factors	Measurement of outcomes in valid and reliable way	Use of appropriate statistical analysis	Overall appraisal	Quality rating
**Fan et al (2017)**	Yes	Yes	Yes	N/A	Yes	Yes	Yes	Yes	Yes	High quality
**Forraster et al (2017)**	Yes	Yes	Yes	N/A	Yes	Yes	Yes	Yes	Yes	High quality
**Fu et al (2015)**	Yes	Yes	Yes	N/A	Yes	Yes	Yes	Yes	Yes	High quality
**Fujiwara et al (2018)**	Yes	Yes	Yes	N/A	No	No	Yes	Yes	Yes	Other quality
**Fukuda et al (2014)**	Yes	Yes	Yes	N/A	Yes	Yes	Yes	Yes	Yes	High quality
**Fukuhara et al (2006)**	Yes	Yes	Yes	N/A	Yes	Yes	Yes	Yes	Yes	High quality
**Gao et al (2015)**	Yes	Yes	Yes	N/A	Yes	Yes	Yes	Yes	Yes	High quality
**Gomez-Sanchez et al (2016)**	Yes	Yes	Yes	N/A	Yes	Yes	Yes	Yes	Yes	High quality
**Gomez-Sanchez et al (2020)**	Yes	Yes	Yes	N/A	Yes	Yes	Yes	Yes	Yes	High quality
**Guo et al (2020)**	Yes	Yes	Yes	N/A	Yes	Yes	Yes	Yes	Yes	High quality
**Hanis et al (2016)**	Yes	Yes	Yes	N/A	Yes	Yes	Yes	Yes	Yes	High quality
**Haraguchi et al (2019)**	Yes	Yes	Yes	N/A	Yes	Yes	Yes	Yes	Yes	High quality
**Higashiura et al (2004)**	Yes	Yes	Yes	N/A	Yes	Yes	Yes	Yes	Yes	High quality
**Hou et al (2018)**	Yes	Yes	Yes	N/A	Yes	Yes	Yes	Yes	Yes	High quality
**Ishida et al (2018)**	Yes	Yes	Yes	N/A	Yes	Yes	Yes	Yes	Yes	High quality
**Jang et al (2014)**	Yes	Yes	Yes	N/A	Yes	Yes	Yes	Yes	Yes	High quality
**Jia et al (2017)**	Yes	Yes	Yes	N/A	Yes	Yes	Yes	Yes	Yes	High quality
**Jiang et al (2020)**	Yes	Yes	Yes	N/A	Yes	Yes	Yes	Yes	Yes	High quality
**Johansson et al (2014)**	Yes	Yes	Yes	N/A	Yes	Yes	Yes	Yes	Yes	High quality
**Jung et al (2013)**	Yes	Yes	Yes	N/A	Yes	Yes	Yes	Yes	Yes	High quality
**Kabutoya et al (2018)**	Yes	Yes	Yes	N/A	No	No	Yes	No	Yes	Other quality
**Kahn et al (2019)**	Yes	Yes	Yes	N/A	Yes	Yes	Yes	Yes	Yes	High quality
**Kawashima-Kumagai et al (2018)**	Yes	Yes	Yes	N/A	Yes	Yes	Yes	Yes	Yes	High quality
**Kido et al (2012)**	Yes	Yes	Yes	N/A	Yes	Yes	Yes	Yes	Yes	High quality
**Kim et al (2011)**	Yes	Yes	Yes	N/A	Yes	Yes	Yes	Yes	Yes	High quality
**Kim et al (2012)**	Yes	Yes	Yes	N/A	Yes	Yes	Yes	Yes	Yes	High quality
**Kim et al (2014)**	Yes	Yes	Yes	N/A	Yes	Yes	Yes	Yes	Yes	High quality
**Kim et al (2015)**	Yes	Yes	Yes	N/A	Yes	Yes	Yes	Yes	Yes	High quality
**Kim et al (2017)**	Yes	Yes	Yes	N/A	Yes	No	Yes	Yes	Yes	Other quality

Study	Criteria for inclusion clearly defined	Detailed description of study subjects and setting	Measurement of exposure in a valid and reliable way	Objective standard criteria for measurement of condition	Identification of confounding factors	Statement of strategies to deal w confounding factors	Measurement of outcomes in valid and reliable way	Use of appropriate statistical analysis	Overall appraisal	Quality rating
**Kim et al (2020)**	Yes	Yes	Yes	No	Yes	Yes	Yes	Yes	Yes	High quality
**Kita et al (2003)**	Yes	No	Yes	N/A	Yes	Yes	Yes	Yes	Yes	Other quality
**Ko et al (2012)**	Yes	Yes	Yes	N/A	Yes	Yes	Yes	Yes	Yes	High quality
**Kong et al (2017)**	Yes	Yes	Yes	N/A	Yes	Yes	Yes	Yes	Yes	High quality
**Kouda et al (2017)**	Yes	Yes	Yes	N/A	Yes	Yes	Yes	Yes	Yes	High quality
**Kozakova et al (2017)**	Yes	Yes	Yes	N/A	Yes	Yes	Yes	Yes	Yes	High quality
**Kullo et al (2005)**	Yes	Yes	Yes	N/A	Yes	Yes	Yes	Yes	Yes	High quality
**Lamballais et al (2018a+b)**	Yes	Yes	Yes	N/A	Yes	Yes	Yes	Yes	Yes	High quality
**Lee et al (2017)**	Yes	Yes	Yes	N/A	Yes	Yes	Yes	Yes	Yes	High quality
**Liao et al (2020)**	Yes	Yes	Yes	N/A	Yes	Yes	Yes	Yes	Yes	High quality
**Lim et al (2012)**	Yes	Yes	Yes	N/A	Yes	Yes	Yes	Yes	Yes	High quality
**Lin et al (2009)**	Yes	Yes	Yes	N/A	Yes	Yes	Yes	Yes	Yes	High quality
**Lin et al (2017)**	Yes	Yes	Yes	N/A	Yes	Yes	Yes	Yes	Yes	High quality
**Liu et al (2018)**	Yes	Yes	Yes	N/A	Yes	Yes	Yes	Yes	Yes	High quality
**Logan et al (2013)**	Yes	Yes	Yes	N/A	Yes	Yes	Yes	Yes	Yes	High quality
**Lopez-Sublet et al (2019)**	Yes	Yes	Yes	N/A	Yes	Yes	Yes	Yes	Yes	High quality
**Lu et al (2020)**	Yes	Yes	Yes	N/A	Yes	Yes	Yes	Yes	Yes	High quality
**Maddock et al (2018)**	Yes	Yes	Yes	N/A	Yes	Yes	Yes	Yes	Yes	High quality
**Magalhaes et al (2013)**	Yes	Yes	Yes	N/A	Yes	Yes	Yes	Yes	Yes	High quality
**Magalhães et al (2019)**	Yes	Yes	Yes	N/A	Yes	Yes	Yes	Yes	Yes	High quality
**Maimaitiaili et al (2020)**	Yes	Yes	Yes	N/A	Yes	Yes	Yes	Yes	Yes	High quality
**Maldonado et al (2011)**	No	No	Yes	Yes	Yes	Yes	Unclear	Unclear	Yes	Other quality
**Matsumoto et al (2020)**	Yes	Yes	Yes	Yes	Yes	Yes	Yes	Yes	Yes	High quality
**Matsuoka et al (2005)**	Unclear	No	Yes	N/A	Yes	Yes	Yes	Yes	Yes	Other quality
**McEniery et al (2010)**	Yes	Yes	Yes	N/A	Yes	Yes	Yes	Yes	Yes	High quality
**Metsämarttila et al (2018)**	Yes	Yes	Yes	N/A	Yes	Yes	Yes	Yes	Yes	High quality
**Michener et al (2015)**	Yes	Yes	Yes	N/A	Yes	Yes	Yes	Yes	Yes	High quality
**Mikumo et al (2009)**	Yes	Yes	Yes	N/A	Yes	Yes	Yes	Yes	Yes	High quality
**Miljkovic et al (2013)**	Yes	Yes	Yes	N/A	Yes	Yes	Yes	Yes	Yes	High quality

Study	Criteria for inclusion clearly defined	Detailed description of study subjects and setting	Measurement of exposure in a valid and reliable way	Objective standard criteria for measurement of condition	Identification of confounding factors	Statement of strategies to deal w confounding factors	Measurement of outcomes in valid and reliable way	Use of appropriate statistical analysis	Overall appraisal	Quality rating
**Mitchell et al (2010)**	Yes	Yes	Yes	N/A	Yes	Yes	Yes	Yes	Yes	High quality
**Miyaki et al (2006)**	Yes	Yes	Yes	N/A	Yes	Yes	Yes	Yes	Yes	High quality
**Mokwatsi et al (2017)**	Yes	Yes	Yes	N/A	Yes	Yes	Yes	Yes	Yes	High quality
**Muhammad et al (2017)**	Yes	Yes	Yes	N/A	Yes	Yes	Yes	Yes	Yes	High quality
**Nakagomi et al (2019)**	Yes	Yes	Yes	N/A	Yes	Yes	Yes	Yes	Yes	High quality
**Nakamura et al (2016)**	Yes	Yes	Yes	N/A	Yes	Yes	Yes	Yes	Yes	High quality
**Nakanishi et al (2005)**	Yes	Yes	Yes	N/A	Yes	Yes	Yes	Yes	Yes	High quality
**Niboshi et al (2006)**	Yes	Yes	Unclear	N/A	Yes	Yes	Yes	Yes	Yes	Other quality
**Ninomiya et al (2013)**	Yes	Yes	Yes	N/A	Yes	Yes	Yes	Yes	Yes	High quality
**Oikonomou et al (2020)**	Yes	Yes	Yes	N/A	Yes	Yes	Yes	Yes	Yes	High quality
**Oren et al (2003)**	Yes	Yes	Yes	N/A	Yes	Yes	Yes	Yes	Yes	High quality
**Oughton et al (2015)**	Yes	Yes	Yes	N/A	Yes	Yes	Yes	Yes	Yes	High quality
**Paini et al (2020)**	Yes	Yes	Yes	N/A	Yes	No	Yes	Yes	Yes	Other quality
**Pan et al (2018)**	Yes	Yes	Yes	N/A	Yes	Yes	Yes	Yes	Yes	High quality
**Pan et al (2019)**	Yes	Yes	Yes	N/A	Yes	Yes	Yes	Yes	Yes	High quality
**Park et al (2012)**	Yes	Yes	Yes	N/A	Yes	Yes	Yes	Yes	Yes	High quality
**Pivin et al (2015)**	Yes	Yes	Yes	N/A	Yes	Yes	Yes	Yes	Yes	High quality
**Podolec et al (2019)**	Yes	Yes	Yes	N/A	Yes	Yes	Yes	Yes	Yes	High quality
**Poon et al (2020)**	Yes	Yes	Yes	N/A	Yes	Yes	Yes	Yes	Yes	High quality
**Recio-Rodriguez et al (2014)**	Yes	Yes	Yes	N/A	Yes	Yes	Yes	Yes	Yes	High quality
**Reusz et al (2010)**	Yes	Yes	Yes	N/A	Yes	Yes	Yes	Yes	Yes	High quality
**Rhee et al (2018)**	Yes	Yes	Yes	N/A	Yes	No	Yes	Yes	Yes	Other quality
**Rojek et al (2018)**	Yes	Yes	Yes	N/A	Yes	Yes	Yes	Yes	Yes	High quality
**Saijo et al (2005)**	Yes	Yes	Yes	N/A	Yes	Yes	Yes	Yes	Yes	High quality
**Saji et al (2012)**	Yes	Yes	Yes	N/A	Yes	Yes	Yes	Yes	Yes	High quality
**Samargandy et al (2020)**	Yes	Yes	Yes	N/A	Yes	Yes	Yes	Yes	Yes	High quality
**Sang et al (2020)**	Yes	Yes	Yes	N/A	Yes	Yes	Yes	Yes	Yes	High quality
**Scuteri et al (2012)**	Yes	Yes	Yes	N/A	Yes	Yes	Yes	Yes	Yes	High quality
**Segers et al (2007)**	Yes	Yes	N/A	N/A	Yes	Yes	Yes	Yes	Yes	Other quality

Study	Criteria for inclusion clearly defined	Detailed description of study subjects and setting	Measurement of exposure in a valid and reliable way	Objective standard criteria for measurement of condition	Identification of confounding factors	Statement of strategies to deal w confounding factors	Measurement of outcomes in valid and reliable way	Use of appropriate statistical analysis	Overall appraisal	Quality rating
**Sforza et al (2019)**	Yes	Yes	Yes	N/A	Yes	No	Yes	Yes	Yes	Other quality
**Sheng et al (2014)**	Yes	Yes	Yes	N/A	Yes	Yes	Yes	Yes	Yes	High quality
**Shokawa et al (2005)**	Unclear	Yes	Yes	N/A	Yes	Yes	Yes	Yes	Yes	Other quality
**Silva et al (2016)**	Yes	Yes	Yes	N/A	Yes	Yes	Yes	Yes	Yes	High quality
**Sonoda et al (2012)**	Yes	Yes	Yes	N/A	Yes	Yes	Yes	Yes	Yes	High quality
**Sougawa et al (2020)**	Yes	Yes	Yes	N/A	Yes	Yes	Yes	Yes	Yes	High quality
**Stamatelopoulos et al (2020)**	Yes	Yes	Yes	N/A	Yes	Yes	Yes	Yes	Yes	High quality
**Strazhesko et al (2017)**	Yes	Yes	Yes	N/A	Yes	Yes	Yes	Yes	Yes	High quality
**Su et al (2014)**	Yes	Yes	Yes	N/A	Yes	Yes	Yes	Yes	Yes	High quality
**Takahashi et al (2021)**	Yes	Yes	Yes	N/A	Yes	Yes	Yes	Yes	Yes	High quality
**Takashima et al (2014)**	Yes	Yes	Yes	N/A	Yes	Yes	Yes	Yes	Yes	High quality
**Tang et al (2020)**	Yes	Yes	Yes	N/A	Yes	Yes	Yes	Yes	Yes	High quality
**Temmar et al (2013)**	Yes	Yes	Yes	N/A	Yes	Yes	Yes	Yes	Yes	High quality
**Tomiyama et al (2003)**	Yes	Yes	Yes	N/A	Yes	Yes	Yes	Yes	Yes	High quality
**Topel et al (2018)**	Yes	Yes	Yes	N/A	Yes	Yes	Yes	Yes	Yes	High quality
**Torigoe et al (2020)**	Yes	Yes	Yes	N/A	Yes	Yes	Yes	Yes	Yes	High quality
**Tsao et al (2018)**	Yes	Yes	Yes	N/A	Yes	Yes	Yes	Yes	Yes	High quality
**Tsuchikura et al (2010)**	Yes	Yes	Yes	N/A	Yes	Yes	Yes	Yes	Yes	High quality
**Uetani et al (2012)**	Yes	Yes	Yes	N/A	Yes	Yes	Yes	Yes	Yes	High quality
**Urbina et al (2012)**	Yes	Yes	Yes	N/A	Yes	Yes	Yes	Yes	Yes	High quality
**Van den Munckhof et al (2017)**	Yes	Yes	Yes	N/A	Yes	Yes	Yes	Yes	Yes	High quality
**Van Varik et al (2017)**	Yes	Yes	Yes	N/A	Yes	Yes	Yes	Yes	Yes	High quality
**Waldstein et al (2008)**	Yes	Yes	Yes	N/A	Yes	Yes	Yes	Yes	Yes	High quality
**Wang et al (2009)**	Yes	Yes	Yes	N/A	Yes	Yes	Yes	Yes	Yes	High quality
**Wang et al (2019)**	Yes	Yes	Yes	N/A	Yes	Yes	Yes	Yes	Yes	High quality
**Watanabe et al (2015)**	Yes	Yes	Yes	N/A	No	No	Unclear	Yes	Yes	Other quality
**Wendell et al (2017)**	Yes	Yes	Yes	N/A	Yes	Yes	Yes	Yes	Yes	High quality
**Wijnands et al (2015)**	Yes	Yes	Yes	N/A	Yes	Yes	Yes	Yes	Yes	High quality
**Wohlfahrt et al (2014)**	Yes	Yes	Yes	N/A	Yes	Yes	Yes	Yes	Yes	High quality

Study	Criteria for inclusion clearly defined	Detailed description of study subjects and setting	Measurement of exposure in a valid and reliable way	Objective standard criteria for measurement of condition	Identification of confounding factors	Statement of strategies to deal w confounding factors	Measurement of outcomes in valid and reliable way	Use of appropriate statistical analysis	Overall appraisal	Quality rating
**Woodiwiss et al (2017)**	Yes	Yes	Yes	N/A	Yes	Yes	Yes	Yes	Yes	High quality
**Wu et al (2019)**	Yes	Yes	Yes	N/A	Yes	Yes	Yes	Yes	Yes	High quality
**Xie et al (2013)**	Yes	Yes	Yes	N/A	Yes	Yes	Yes	Yes	Yes	High quality
**Xuan et al (2019)**	Yes	Yes	Yes	N/A	Yes	Yes	Yes	Yes	Yes	High quality
**Yang et al (2014)**	Unclear	Yes	Yes	N/A	Yes	Yes	Yes	Yes	Yes	Other quality
**Zachariah et al (2018)**	Yes	Yes	Yes	N/A	Yes	Yes	Yes	Yes	Yes	High quality
**Zaniqueli et al (2017)**	Yes	Yes	Yes	N/A	Yes	Yes	Yes	Yes	Yes	High quality
**Zeng et al (2017)**	Yes	Yes	Yes	N/A	Yes	Yes	Yes	Yes	Yes	High quality
**Zhang et al (2017)**	Yes	Yes	Yes	N/A	Yes	Yes	Yes	Yes	Yes	High quality
**Zhang et al (2020)**	Yes	Yes	Yes	N/A	Yes	Yes	Yes	Yes	Yes	High quality
**Zhao et al (2014)**	Yes	Yes	Yes	N/A	Yes	Yes	Yes	Yes	Yes	High quality
**Zheng et al (2015)**	Yes	Yes	Yes	N/A	Yes	No	Yes	Yes	Yes	Other quality
**Zhou et al (2017)**	Yes	Yes	Yes	N/A	Yes	Yes	Yes	Yes	Yes	High quality
**Zureik et al (2002)**	Yes	Yes	Yes	N/A	Yes	Yes	Yes	Yes	Yes	High quality
**Zureik et al (2006)**	Yes	Yes	Yes	N/A	Yes	Yes	Yes	Yes	Yes	High quality

Supplemental Table 3: Quality rating of included studies.

Rating of study quality used the Joanna Briggs Instrument for Analytical Cross Sectional Studies. The rating item "objective standard criteria for measurement of condition" was not applicable and was not considered because only generally healthy subjects not selected for presence of any defined condition were included. Studies were considered of high quality if all other seven criteria were fulfilled. Fewer studies are shown here compared to Supplemental Table 2 because the rating instrument was not applicable to included data from unpublished studies.

							Years of age				
**Country**	**Sex**	**Percentile**	**0–9**	**10–19**	**20–29**	**30–39**	**40–49**	**50–59**	**60–69**	**70–79**	**80–89**
Total	Female	5th			8·60	8·99	9·67	10·7	12·0	13·2	13·8
		25th			9·58	10·1	10·9	12·2	13·8	15·5	16·9
		50th			10·3	10·9	11·9	13·4	15·3	17·5	19·3
		75th			11·1	11·9	13·0	14·9	17·2	19·7	21·8
		95th			12·4	13·4	15·1	17·6	20·7	23·6	25·8
	Male	5th			9·53	10·2	10·6	11·2	12·0	13·1	14·3
		25th			10·6	11·3	11·8	12·6	13·8	15·4	17·1
		50th			11·3	12·2	12·8	13·8	15·4	17·3	19·3
		75th			12·2	13·2	14·0	15·3	17·3	19·5	21·7
		95th			13·5	14·8	15·9	17·9	20·7	23·2	25·4
China	Female	5th			8·58	8·97	9·67	10·7	11·9	13·1	14·5
		25th			9·89	10·3	11·1	12·4	14·2	15·9	17·6
		50th			10·8	11·3	12·2	13·9	16·2	18·3	20·1
		75th			11·8	12·4	13·6	15·8	18·6	21·0	22·7
		95th			13·4	14·3	16·2	19·3	23·3	25·7	26·8
	Male	5th			9·50	10·1	10·6	11·1	12·0	13·1	14·3
		25th			10·8	11·5	12·0	12·7	14·0	15·6	17·3
		50th			11·8	12·6	13·2	14·1	15·8	17·7	19·7
		75th			12·8	13·8	14·6	15·9	18·1	20·3	22·5
		95th			14·5	15·9	17·3	19·5	22·6	24·9	27·2
Japan	Female	5th				8·96	9·68	10·6	11·8	12·8	
		25th				9·83	10·7	11·9	13·6	15·5	
		50th				10·5	11·6	13·0	15·1	17·6	
		75th				11·4	12·7	14·4	17·0	20·0	
		95th				12·8	14·7	17·2	20·4	23·9	
	Male	5th				10·3	10·7	11·3	12·3	13·1	
		25th				11·3	11·9	12·7	14·1	15·6	
		50th				12·1	12·8	13·8	15·7	17·8	
		75th				13·0	13·9	15·2	17·7	20·3	
		95th				14·3	15·6	17·7	21·2	24·7	
South Korea	Female	5th				9·46	10·2	10·9	12·1	13·1	13·4
		25th				10·9	11·6	12·4	13·8	15·6	16·8

Country	Sex	Percentile	0–9	10–19	20–29	30–39	40–49	50–59	60–69	70–79	80–89
						Years of age					
		50th				11·9	12·7	13·6	15·4	17·6	19·4
		75th				13·0	14·0	15·1	17·3	20·0	22·1
		95th				14·8	16·2	17·9	20·9	24·0	26·2
	Male	5th				10·7	10·9	11·2	11·9	12·6	12·6
		25th				11·9	12·2	12·7	13·8	15·2	16·3
		50th				12·9	13·3	14·0	15·4	17·3	19·0
		75th				14·0	14·6	15·6	17·4	19·7	21·7
		95th				16·1	17·0	18·5	20·9	23·6	25·6
Switzerland	Female	5th						9·98	11·1	12·5	13·3
		25th						11·0	12·5	14·3	15·3
		50th						11·9	13·6	15·7	16·9
		75th						12·9	14·9	17·4	18·8
		95th						14·7	17·2	20·3	22·2
	Male	5th						10·4	11·3	12·4	13·0
		25th						11·4	12·7	14·3	15·2
		50th						12·3	13·8	15·8	17·0
		75th						13·3	15·2	17·6	19·0
		95th						15·2	17·7	20·8	22·5

Supplemental Table 4a: Reference values for baPWV by age, sex and country.

For each group defined by country, sex, and decade of age, percentiles 5, 25, 50, 75, and 95 of baPWV in meters per second are given. “Total” denotes the estimates derived from pooling all countries.

Country	Sex	Percentile	Years of age 0–9	10–19	20–29	30–39	40–49	50–59	60–69	70–79	80–89
Total	Female	5th	3·84	4·67	5·12	5·51	5·98	6·39	6·95	7·58	8·00
		25th	4·43	5·26	5·83	6·30	6·86	7·46	8·34	9·39	10·5
		50th	4·90	5·72	6·42	6·95	7·60	8·39	9·54	11·0	12·7
		75th	5·44	6·25	7·09	7·72	8·48	9·50	11·0	12·9	15·4
		95th	6·38	7·14	8·25	9·08	10·1	11·6	13·8	16·5	20·4
	Male	5th	4·07	4·82	5·57	5·92	6·31	6·77	7·37	8·17	9·10
		25th	4·54	5·43	6·35	6·80	7·30	7·90	8·74	9·98	11·6
		50th	4·95	5·93	6·97	7·52	8·12	8·87	9·97	11·6	13·8
		75th	5·44	6·51	7·68	8·33	9·09	10·1	11·5	13·7	16·7
		95th	6·37	7·54	8·89	9·72	10·8	12·3	14·5	17·8	22·3
Argentina	Female	5th		4·27	4·83	4·81	5·08	5·72			
		25th		4·76	5·46	5·55	6·05	6·87			
		50th		5·09	5·90	6·09	6·78	7·72			
		75th		5·41	6·35	6·66	7·55	8·60			
		95th		5·87	7·01	7·53	8·74	9·93			
	Male	5th		4·23	4·66	4·70	5·02	5·78			
		25th		4·72	5·42	5·64	6·10	6·99			
		50th		5·09	6·02	6·39	6·94	7·88			
		75th		5·51	6·68	7·22	7·86	8·83			
		95th		6·19	7·79	8·59	9·31	10·3			
Australia	Female	5th		3·88	4·42	4·86	5·23	5·64			
		25th		4·29	4·99	5·59	6·07	6·51			
		50th		4·63	5·45	6·17	6·74	7·21			
		75th		5·02	5·97	6·82	7·49	8·02			
		95th		5·71	6·86	7·89	8·74	9·37			
	Male	5th		4·01	4·94	5·61	6·03				
		25th		4·45	5·50	6·27	6·75				
		50th		4·82	5·96	6·80	7·33				
		75th		5·26	6·50	7·41	7·99				
		95th		6·05	7·46	8·48	9·12				
Belgium	Female	5th				4·50	4·89	5·27	5·39		

Country	Sex	Percentile	Years of age<br>0–9	10–19	20–29	30–39	40–49	50–59	60–69	70–79	80–89
		25th				5·06	5·60	6·26	6·60		
		50th				5·56	6·23	7·14	7·69		
		75th				6·21	7·03	8·25	9·06		
		95th				7·60	8·67	10·5	11·7		
	Male	5th				4·53	4·99	5·34	5·45		
		25th				5·16	5·70	6·21	6·43		
		50th				5·69	6·31	7·05	7·44		
		75th				6·30	7·07	8·21	8·99		
		95th				7·37	8·51	11·1	13·7		
Brazil	Female	5th	4·11	4·52	5·09	5·68	6·14	6·51	6·97	7·31	
		25th	4·61	5·09	5·78	6·50	7·08	7·64	8·40	9·03	
		50th	5·02	5·57	6·35	7·18	7·88	8·62	9·68	10·6	
		75th	5·50	6·12	7·03	8·00	8·83	9·80	11·3	12·6	
		95th	6·35	7·11	8·24	9·46	10·5	12·0	14·3	16·5	
	Male	5th	4·03	4·55	5·33	6·19	6·54	6·94	7·44	7·88	
		25th	4·55	5·16	6·10	7·14	7·63	8·22	8·92	9·55	
		50th	5·00	5·68	6·73	7·91	8·53	9·32	10·3	11·1	
		75th	5·55	6·31	7·47	8·80	9·57	10·7	12·0	13·2	
		95th	6·62	7·46	8·75	10·3	11·4	13·1	15·5	17·7	
France	Female	5th					7·07	7·35			
		25th					7·98	8·55			
		50th					8·75	9·53			
		75th					9·68	10·6			
		95th					11·4	12·5			
	Male	5th					7·55	7·74	8·63		
		25th					8·46	8·93	9·99		
		50th					9·24	9·95	11·3		
		75th					10·2	11·2	13·1		
		95th					12·1	13·4	17·6		
Italy	Female	5th		4·71	5·17	5·63	6·02	6·31	6·52	6·64	
		25th		5·24	5·85	6·51	7·13	7·69	8·22	8·71	

Country	Sex	Percentile	Years of age								
			0–9	10–19	20–29	30–39	40–49	50–59	60–69	70–79	80–89
		50th		5·69	6·43	7·27	8·08	8·86	9·60	10·3	
		75th		6·23	7·13	8·20	9·24	10·2	11·2	12·1	
		95th		7·23	8·45	9·94	11·4	12·7	13·8	14·8	
	Male	5th		4·69	5·18	5·79	6·34	6·77	6·97	6·91	
		25th		5·36	5·95	6·69	7·40	8·06	8·61	9·05	
		50th		5·89	6·56	7·40	8·24	9·08	9·92	10·8	
		75th		6·50	7·25	8·20	9·17	10·2	11·4	12·7	
		95th		7·52	8·40	9·51	10·7	12·0	13·7	15·8	
Portugal	Female	5th	4·33	5·31	6·21	6·77	7·14	7·52	8·11		
		25th	4·90	5·99	7·12	7·92	8·50	9·08	9·83		
		50th	5·31	6·50	7·84	8·86	9·61	10·3	11·1		
		75th	5·73	7·04	8·64	9·93	10·9	11·7	12·5		
		95th	6·36	7·90	9·97	11·8	13·0	14·0	14·7		
	Male	5th	4·43	5·47	6·86	7·10	7·51	8·00	8·66		
		25th	4·92	6·14	7·81	8·18	8·78	9·49	10·4		
		50th	5·29	6·65	8·54	9·03	9·77	10·7	11·8		
		75th	5·69	7·20	9·32	9·95	10·9	12·0	13·4		
		95th	6·31	8·06	10·6	11·4	12·6	14·1	15·9		
South Africa	Female	5th			4·85	5·17	5·56	5·91	6·18		
		25th			5·42	5·87	6·45	7·03	7·59		
		50th			5·89	6·46	7·19	7·98	8·78		
		75th			6·44	7·16	8·08	9·11	10·2		
		95th			7·42	8·39	9·66	11·1	12·7		
	Male	5th			5·57						
		25th			6·19						
		50th			6·69						
		75th			7·26						
		95th			8·27						
Uruguay	Female	5th	3·74	4·22	4·99	5·49	5·91	6·21	6·83	7·53	7·87
		25th	4·35	4·91	5·81	6·39	6·90	7·30	8·11	9·10	9·64
		50th	4·86	5·47	6·46	7·12	7·72	8·23	9·22	10·5	11·2

Country	Sex	Percentile	0–9	10–19	20–29	30–39	40–49	50–59	60–69	70–79	80–89
							Years of age				
		75th	5·44	6·08	7·18	7·95	8·68	9·34	10·6	12·1	13·0
		95th	6·45	7·11	8·36	9·34	10·4	11·4	13·0	15·1	16·2
	Male	5th	3·75	4·42	5·35	5·54	6·00	6·29	6·98	7·92	8·52
		25th	4·32	5·04	6·14	6·43	7·06	7·42	8·24	9·41	10·1
		50th	4·81	5·54	6·75	7·12	7·91	8·37	9·33	10·8	11·7
		75th	5·40	6·11	7·41	7·89	8·89	9·48	10·6	12·5	13·7
		95th	6·51	7·06	8·47	9·12	10·5	11·4	13·1	16·0	18·0
USA	Female	5th			4·99	5·44	5·83	6·33	7·05	7·90	8·57
		25th			5·62	6·14	6·61	7·33	8·53	10·1	11·8
		50th			6·10	6·71	7·28	8·22	9·89	12·2	14·7
		75th			6·62	7·36	8·09	9·34	11·7	14·9	18·3
		95th			7·44	8·50	9·61	11·6	15·4	20·4	25·2
	Male	5th			5·35	5·87	6·29	6·73	7·49	8·52	9·42
		25th			6·02	6·66	7·23	7·88	9·01	10·6	12·1
		50th			6·58	7·33	8·03	8·90	10·4	12·5	14·8
		75th			7·23	8·12	9·00	10·2	12·2	15·2	18·5
		95th			8·39	9·55	10·8	12·6	15·8	20·8	27·0

Supplemental Table 4b: Reference values for cfPWV by age, sex and country.

For each group defined by country, sex, and decade of age, percentiles 5, 25, 50, 75, and 95 of cfPWV in meters per second are given. “Total” denotes the estimates derived from pooling all countries.

Variable	Category	All data<br>Mean PWV (95% CI)	No. participants	No. studies	P	Only fully published data<br>Mean PWV (95% CI)	No. participants	No. studies	P
All Data		7.45 (7.11, 7.79)	166375	93		7.54 (7.17, 7.90)	155143	85	
Country	Austria	7.33 (5.68, 8.98)	2696	2	<0.0001	Not available	Not available	0	<0.0001
	Brazil	7.35 (6.56, 8.14)	26027	9		7.68 (6.71, 8.64)	21048	6	
	Greece	7.89 (6.74, 9.04)	3651	4		7.38 (6.02, 8.73)	2811	3	
	Portugal	7.91 (6.58, 9.23)	5877	3		8.15 (6.51, 9.80)	3953	2	
	South Africa	6.36 (5.20, 7.52)	2876	4		6.31 (4.93, 7.68)	2083	3	
Country Income	High Income	7.32 (6.98, 7.66)	96245	64	<0.0001	7.41 (7.05, 7.77)	90785	60	<0.0001
	Other Income	7.74 (7.38, 8.09)	70130	30		7.83 (7.46, 8.21)	64358	26	
	Complior	8.48 (7.97, 8.98)	45591	28		8.59 (8.03, 9.14)	41359	25	
	Sphygmocor	7.00 (6.55, 7.46)	53799	36		7.13 (6.63, 7.62)	48027	32	
	Vicorder	6.76 (5.71, 7.82)	15815	6		6.68 (5.48, 7.87)	14587	5	
Measurement Method	Tonometry	7.62 (7.25, 7.98)	132698	76	0.080	7.72 (7.33, 8.11)	122694	69	0.071
	Oscillometry	6.77 (5.90, 7.64)	22536	11		6.77 (5.84, 7.71)	21308	10	
Path Length Measure	Difference, Measured	6.94 (6.39, 7.49)	58595	28	0.13	6.97 (6.35, 7.59)	50464	23	0.13
	Absolute, Measured	7.88 (7.31, 8.44)	50391	27		7.91 (7.31, 8.51)	48083	25	
	Absolute, Measured, C.O.A.D.	8.10 (6.95, 9.25)	13175	6		8.51 (7.21, 9.80)	12382	5	
Region	Europe	7.50 (7.07, 7.93)	68623	47	<0.0001	7.57 (7.11, 8.03)	63163	43	<0.0001
	Africa	7.91 (7.46, 8.36)	4037	8		7.98 (7.50, 8.46)	3244	7	
	South America	7.19 (6.30, 8.08)	31280	11		7.40 (6.33, 8.46)	26301	8	
Study quality	Unknown	7.89 (7.00, 8.77)	23417	11	0.44	9.36 (7.68, 11.05)	12185	3	0.078
Study Size (n)	2000+	7.71 (7.06, 8.36)	104436	21	0.52	7.84 (7.15, 8.52)	102124	20	0.47
	500-2000	7.30 (6.87, 7.73)	56693	52		7.36 (6.88, 7.83)	47773	45	
Study Year	>2015	7.11 (6.35, 7.88)	43691	15	0.33	7.22 (6.21, 8.23)	34092	9	0.51
	≤2015	7.52 (7.16, 7.89)	122684	78		7.57 (7.19, 7.96)	121051	76	

Supplemental Table 5: Sensitivity analysis investigating the impact of restricting analysis to fully published data.
Only results for cfPWV are shown because no unpublished data were used for baPWV analyses. For cfPWV, there were 6 unpublished and 2 partly published datasets, representing 11232 subjects. Only effects for which excluding unpublished datasets changed No. studies or No. subjects are shown.
Mean PWV indicates age-standardised average PWV values in meters per second and 95% confidence intervals. Number of participants and number of studies give the count of studies and participants used for each estimate in each subgroup. P (any diff.) is for any difference in standardised average PWV between category subgroups.
No.=number of. C.O.A.D., Corrected Or Alternative Distance.

References

1. Rigby RA, Stasinopoulos DM. Generalized additive models for location, scale and shape. *Journal of the Royal Statistical Society: Series C (Applied Statistics)* 2005; **54**(3): 507-54.
2. Hastie T, Tibshirani R. Varying-Coefficient Models. *Journal of the Royal Statistical Society Series B (Methodological)* 1993; **55**(4): 757-96.
3. Achimastos AD, Efstathiou SP, Christoforatos T, Panagiotou TN, Stergiou GS, Mountokalakis TD. Arterial stiffness: determinants and relationship to the metabolic syndrome. *Angiology* 2007; **58**(1): 11-20.
4. Ai ZS, Li J, Liu ZM, et al. Reference value of brachial-ankle pulse wave velocity for the eastern Chinese population and potential influencing factors. *Braz J Med Biol Res* 2011; **44**(10): 1000-5.
5. Araghi M, Shipley MJ, Wilkinson IB, et al. Association of aortic stiffness with cognitive decline: Whitehall II longitudinal cohort study. *Eur J Epidemiol* 2020; **35**(9): 861-9.
6. Avramovski P, Avramovska M, Sikole A. Bone Strength and Arterial Stiffness Impact on Cardiovascular Mortality in a General Population. *J Osteoporos* 2016; **2016**: 7030272.
7. Baier D, Teren A, Wirkner K, Loeffler M, Scholz M. Parameters of pulse wave velocity: determinants and reference values assessed in the population-based study LIFE-Adult. *Clin Res Cardiol* 2018; **107**(11): 1050-61.
8. Baldo MP, Cunha RS, Ribeiro ALP, et al. Racial Differences in Arterial Stiffness are Mainly Determined by Blood Pressure Levels: Results From the ELSA-Brasil Study. *J Am Heart Assoc* 2017; **6**(6).
9. Benetos A, Zervoudaki A, Kearney-Schwartz A, et al. Effects of lean and fat mass on bone mineral density and arterial stiffness in elderly men. *Osteoporos Int* 2009; **20**(8): 1385-91.
10. Bérard E, Bongard V, Ruidavets JB, Amar J, Ferrières J. Pulse wave velocity, pulse pressure and number of carotid or femoral plaques improve prediction of cardiovascular death in a population at low risk. *J Hum Hypertens* 2013; **27**(9): 529-34.
11. Bia D, Zócalo Y. Physiological Age- and Sex-Related Profiles for Local (Aortic) and Regional (Carotid-Femoral, Carotid-Radial) Pulse Wave Velocity and Center-to-Periphery Stiffness Gradient, with and without Blood Pressure Adjustments: Reference Intervals and Agreement between Methods in Healthy Subjects (3-84 Years). *J Cardiovasc Dev Dis* 2021; **8**(1).
12. Bian S, Guo H, Ye P, Luo L, Wu H, Xiao W. Serum uric Acid level and diverse impacts on regional arterial stiffness and wave reflection. *Iran J Public Health* 2012; **41**(8): 33-41.
13. Botha D, Breet Y, Schutte AE. Comparing the associations of clinic vs. ambulatory blood pressure with subclinical organ damage in young healthy adults: the African-PREDICT study. *Hypertens Res* 2021; **44**(7): 840-9.
14. Cecelja M, Jiang B, Bevan L, Frost ML, Spector TD, Chowienczyk PJ. Arterial stiffening relates to arterial calcification but not to noncalcified atheroma in women. A twin study. *J Am Coll Cardiol* 2011; **57**(13): 1480-6.
15. Cecelja M, Sriswan R, Kulkarni B, Kinra S, Nitsch D. Association of pulse wave velocity and intima-media thickness with cardiovascular risk factors in young adults. *J Clin Hypertens (Greenwich)* 2020; **22**(2): 174-84.
16. Ceponiene I, Klumbiene J, Tamuleviciute-Prasciene E, et al. Associations between risk factors in childhood (12-13 years) and adulthood (48-49 years) and subclinical atherosclerosis: the Kaunas Cardiovascular Risk Cohort Study. *BMC Cardiovasc Disord* 2015; **15**: 89.
17. Chen L, Wang B, Wang J, et al. Association between serum total homocysteine and arterial stiffness in adults: a community-based study. *J Clin Hypertens (Greenwich)* 2018; **20**(4): 686-93.
18. Cho J, Baek HJ. A Comparative Study of Brachial-Ankle Pulse Wave Velocity and Heart-Finger Pulse Wave Velocity in Korean Adults. *Sensors (Basel)* 2020; **20**(7).
19. Choi SW, Kim HY, Lee YH, et al. eGFR is associated with subclinical atherosclerosis independent of albuminuria: the Dong-gu Study. *Atherosclerosis* 2010; **212**(2): 661-7.

20. Choi HY, Kim SH, Choi AR, et al. Hyperuricemia and risk of increased arterial stiffness in healthy women based on health screening in Korean population. *PLoS One* 2017; **12**(6): e0180406.
21. Chou LP, Li CY, Hu SC. Work-Related Psychosocial Hazards and Arteriosclerosis. *Int Heart J* 2015; **56**(6): 644-50.
22. Chuang SY, Chen CH, Cheng CM, Chou P. Combined use of brachial-ankle pulse wave velocity and ankle-brachial index for fast assessment of arteriosclerosis and atherosclerosis in a community. *Int J Cardiol* 2005; **98**(1): 99-105.
23. Cicero AFG, Fogacci F, Tocci G, et al. Awareness of major cardiovascular risk factors and its relationship with markers of vascular aging: Data from the Brisighella Heart Study. *Nutr Metab Cardiovasc Dis* 2020; **30**(6): 907-14.
24. Cruz VPD, Gonzaga CWO, da Silva VB, et al. Arterial stiffness in black adults from Angola and Brazil. *J Clin Hypertens (Greenwich)* 2020; **22**(8): 1469-75.
25. Cunha PG, Cotter J, Oliveira P, et al. Pulse wave velocity distribution in a cohort study: from arterial stiffness to early vascular aging. *J Hypertens* 2015; **33**(7): 1438-45.
26. de Mendonça GS, de Souza DF, de Alvarenga Cunha Brunelli AC, et al. Arterial stiffness in elderly patients with normotension and hypertension in Brazil. *J Clin Hypertens (Greenwich)* 2018; **20**(9): 1285-93.
27. de Oliveira Alvim R, Mourao-Junior CA, de Oliveira CM, et al. Glycemic control and arterial stiffness in a Brazilian rural population: Baependi Heart Study. *Diabetol Metab Syndr* 2015; **7**: 86.
28. Del Giorno R, Troiani C, Gabutti S, Stefanelli K, Gabutti L. Comparing oscillometric and tonometric methods to assess pulse wave velocity: a population-based study. *Ann Med* 2021; **53**(1): 1-16.
29. Diaz A, Zócalo Y, Bia D, Wray S, Fischer EC. Reference intervals and percentiles for carotid-femoral pulse wave velocity in a healthy population aged between 9 and 87 years. *J Clin Hypertens (Greenwich)* 2018; **20**(4): 659-71.
30. Ellins EA, Smith KE, Lennon LT, et al. Arterial pathophysiology and comparison of two devices for pulse wave velocity assessment in elderly men: the British regional heart study. *Open Heart* 2017; **4**(2): e000645.
31. Fan X, Kalim S, Ye W, et al. Urinary Stone Disease and Cardiovascular Disease Risk in a Rural Chinese Population. *Kidney Int Rep* 2017; **2**(6): 1042-9.
32. Foraster M, Eze IC, Schaffner E, et al. Exposure to Road, Railway, and Aircraft Noise and Arterial Stiffness in the SAPALDIA Study: Annual Average Noise Levels and Temporal Noise Characteristics. *Environ Health Perspect* 2017; **125**(9): 097004.
33. Fu S, Luo L, Ye P, Xiao W. Multimarker Analysis for New Biomarkers in Relation to Central Arterial Stiffness and Hemodynamics in a Chinese Community-Dwelling Population. *Angiology* 2015; **66**(10): 950-6.
34. Fujiwara H, Nakajima H, Inoue F, Kosaka K, Asano H, Yoshii K. Arterial stiffness in junior high school students: Longitudinal observations. *Pediatr Int* 2018; **60**(2): 127-35.
35. Fukuda T, Hamaguchi M, Kojima T, et al. Association between serum γ-glutamyltranspeptidase and atherosclerosis: a population-based cross-sectional study. *BMJ Open* 2014; **4**(10): e005413.
36. Fukuhara M, Matsumura K, Ansai T, et al. Prediction of cognitive function by arterial stiffness in the very elderly. *Circ J* 2006; **70**(6): 756-61.
37. Gao J, Bao M, Liu Y, et al. Changes in cardiovascular health score and atherosclerosis progression in middle-aged and older persons in China: a cohort study. *BMJ Open* 2015; **5**(8): e007547.
38. Gomez-Sanchez L, Garcia-Ortiz L, Patino-Alonso MC, et al. Association of metabolic syndrome and its components with arterial stiffness in Caucasian subjects of the MARK study: a cross-sectional trial. *Cardiovasc Diabetol* 2016; **15**(1): 148.

39. Gómez-Sánchez M, Patino-Alonso MC, Gómez-Sánchez L, et al. Reference values of arterial stiffness parameters and their association with cardiovascular risk factors in the Spanish population. The EVA Study. *Rev Esp Cardiol (Engl Ed)* 2020; **73**(1): 43-52.
40. Guo QH, Muhammad IF, Borné Y, et al. Difference in the risk profiles of carotid-femoral pulse wave velocity: results from two community-based studies in China and Sweden. *J Hum Hypertens* 2020; **34**(3): 207-13.
41. Hanis CL, Redline S, Cade BE, et al. Beyond type 2 diabetes, obesity and hypertension: an axis including sleep apnea, left ventricular hypertrophy, endothelial dysfunction, and aortic stiffness among Mexican Americans in Starr County, Texas. *Cardiovasc Diabetol* 2016; **15**: 86.
42. Haraguchi N, Koyama T, Kuriyama N, et al. Assessment of anthropometric indices other than BMI to evaluate arterial stiffness. *Hypertens Res* 2019; **42**(10): 1599-605.
43. Higashiura K, Ura N, Ohata J, et al. Correlations of adiponectin level with insulin resistance and atherosclerosis in Japanese male populations. *Clin Endocrinol (Oxf)* 2004; **61**(6): 753-9.
44. Hou D, Yan Y, Liu J, Zhao X, Cheng H, Mi J. Childhood pulse pressure predicts subclinical vascular damage in adulthood: the Beijing Blood Pressure Cohort Study. *J Hypertens* 2018; **36**(8): 1663-70.
45. Ikram MA, Brusselle G, Ghanbari M, et al. Objectives, design and main findings until 2020 from the Rotterdam Study. *Eur J Epidemiol* 2020; **35**(5): 483-517.
46. Ishida A, Fujisawa M, Del Saz EG, et al. Arterial stiffness, not systolic blood pressure, increases with age in native Papuan populations. *Hypertens Res* 2018; **41**(7): 539-46.
47. Jang SY, Ju EY, Huh EH, Kim JH, Kim DK. Determinants of brachial-ankle pulse wave velocity and carotid-femoral pulse wave velocity in healthy Koreans. *J Korean Med Sci* 2014; **29**(6): 798-804.
48. Jia L, Zhang W, Ma J, et al. Pulse Pressure, Instead of Brachium-Ankle Pulse Wave Velocity, is Associated with Reduced Kidney Function in a Chinese Han Population. *Kidney Blood Press Res* 2017; **42**(1): 43-51.
49. Jiang Y, Fan F, Jia J, et al. Brachial-ankle pulse wave velocity is independently associated with urine albumin-to-creatinine ratio in a Chinese community-based cohort. *Int Urol Nephrol* 2020; **52**(4): 713-20.
50. Johansson JK, Puukka PJ, Jula AM. Interarm blood pressure difference and target organ damage in the general population. *J Hypertens* 2014; **32**(2): 260-6.
51. Jung SK, Kim MK, Lee YH, et al. Lower zinc bioavailability may be related to higher risk of subclinical atherosclerosis in Korean adults. *PLoS One* 2013; **8**(11): e80115.
52. Kabutoya T, Kario K. Comparative Assessment of Cutoffs for the Cardio-Ankle Vascular Index and Brachial-Ankle Pulse Wave Velocity in a Nationwide Registry: A Cardiovascular Prognostic Coupling Study. *Pulse (Basel)* 2019; **6**(3-4): 131-6.
53. Kahn FK, Wake M, Lycett K, et al. Vascular function and stiffness: population epidemiology and concordance in Australian children aged 11-12 years and their parents. *BMJ Open* 2019; **9**(Suppl 3): 34-43.
54. Kawashima-Kumagai K, Tabara Y, Yamashiro K, et al. Association of retinal vessel calibers and longitudinal changes in arterial stiffness: the Nagahama study. *J Hypertens* 2018; **36**(3): 587-93.
55. Kido M, Kohara K, Miyawaki S, Tabara Y, Igase M, Miki T. Perceived age of facial features is a significant diagnosis criterion for age-related carotid atherosclerosis in Japanese subjects: J-SHIPP study. *Geriatr Gerontol Int* 2012; **12**(4): 733-40.
56. Kim TN, Park MS, Lim KI, et al. Skeletal muscle mass to visceral fat area ratio is associated with metabolic syndrome and arterial stiffness: The Korean Sarcopenic Obesity Study (KSOS). *Diabetes Res Clin Pract* 2011; **93**(2): 285-91.
57. Kim BJ, Kim NH, Kim BS, Kang JH. The association between nonalcoholic fatty liver disease, metabolic syndrome and arterial stiffness in nondiabetic, nonhypertensive individuals. *Cardiology* 2012; **123**(1): 54-61.
58. Kim NH, Park J, Kim SH, et al. Non-alcoholic fatty liver disease, metabolic syndrome and subclinical cardiovascular changes in the general population. *Heart* 2014; **100**(12): 938-43.

59. Kim CW, Chang Y, Zhao D, et al. Sleep Duration, Sleep Quality, and Markers of Subclinical Arterial Disease in Healthy Men and Women. *Arterioscler Thromb Vasc Biol* 2015; **35**(10): 2238-45.
60. Kim YH, So WY. Relative lower body circumferences are associated with the prevalence of metabolic syndrome and arterial stiffness. *Technol Health Care* 2017; **25**(2): 211-9.
61. Kim HL, Lee JY, Lim WH, et al. Relationship of Socioeconomic Status to Arterial Stiffness: Comparison Between Medical Aid Beneficiaries and National Health Insurance Beneficiaries. *Am J Hypertens* 2020; **33**(8): 718-25.
62. Kita T, Kitamura K, Hashida S, Morishita K, Eto T. Plasma adrenomedullin is closely correlated with pulse wave velocity in middle-aged and elderly patients. *Hypertens Res* 2003; **26**(11): 887-93.
63. Ko MJ, Kim MK, Shin J, Choi BY. Relations of pulse wave velocity to waist circumference independent of hip circumference. *Epidemiol Health* 2010; **32**: e2010004.
64. Kong X, Ma X, Tang L, et al. Arterial stiffness evaluated by carotid-femoral pulse wave velocity increases the risk of chronic kidney disease in a Chinese population-based cohort. *Nephrology (Carlton)* 2017; **22**(3): 205-12.
65. Kouda K, Dongmei N, Tamaki J, et al. Relative Importance of Central and Peripheral Adiposities on Cardiometabolic Variables in Females: A Japanese Population-Based Study. *J Clin Densitom* 2017; **20**(1): 58-65.
66. Kozakova M, Morizzo C, La Carrubba S, et al. Associations between common carotid artery diameter, Framingham risk score and cardiovascular events. *Nutr Metab Cardiovasc Dis* 2017; **27**(4): 329-34.
67. Kullo IJ, Seward JB, Bailey KR, et al. C-reactive protein is related to arterial wave reflection and stiffness in asymptomatic subjects from the community. *Am J Hypertens* 2005; **18**(8): 1123-9.
68. Lee JH, Suh HS. Association of Serum 25-hydroxy-vitamin D Concentration and Arterial Stiffness among Korean Adults in Single Center. *J Bone Metab* 2017; **24**(1): 51-8.
69. Liao YY, Chu C, Wang Y, et al. Sex differences in impact of long-term burden and trends of body mass index and blood pressure from childhood to adulthood on arterial stiffness in adults: A 30-year cohort study. *Atherosclerosis* 2020; **313**: 118-25.
70. Lim S, Choi HJ, Shin H, et al. Subclinical atherosclerosis in a community-based elderly cohort: the Korean Longitudinal Study on Health and Aging. *Int J Cardiol* 2012; **155**(1): 126-33.
71. Lin WY, Lai MM, Li CI, et al. In addition to insulin resistance and obesity, brachial-ankle pulse wave velocity is strongly associated with metabolic syndrome in Chinese--a population-based study (Taichung Community Health Study, TCHS). *J Atheroscler Thromb* 2009; **16**(2): 105-12.
72. Lin L, Peng K, Du R, et al. High glomerular filtration rate is associated with arterial stiffness in Chinese population. *J Hypertens* 2017; **35**(2): 385-91.
73. Liu H, Liu J, Zhao H, Zhou Y, Li L, Wang H. Relationship between Serum Uric Acid and Vascular Function and Structure Markers and Gender Difference in a Real-World Population of China-From Beijing Vascular Disease Patients Evaluation Study (BEST) Study. *J Atheroscler Thromb* 2018; **25**(3): 254-61.
74. Logan JG, Barksdale DJ. Pulse wave velocity in Korean American men and women. *J Cardiovasc Nurs* 2013; **28**(1): 90-6.
75. Lopez-Sublet M, Girerd N, Bozec E, et al. Nondipping Pattern and Cardiovascular and Renal Damage in a Population-Based Study (The STANISLAS Cohort Study). *Am J Hypertens* 2019; **32**(7): 620-8.
76. Ji H, Kim A, Ebinger JE, et al. Sex Differences in Blood Pressure Trajectories Over the Life Course. *JAMA Cardiol* 2020; **5**(3): 19-26.
77. Maddock J, Ziauddeen N, Ambrosini GL, Wong A, Hardy R, Ray S. Adherence to a Dietary Approaches to Stop Hypertension (DASH)-type diet over the life course and associated vascular function: a study based on the MRC 1946 British birth cohort. *Br J Nutr* 2018; **119**(5): 581-9.
78. Magalhães P, Capingana DP, Silva AB, et al. Age- and gender-specific reference values of pulse wave velocity for African adults: preliminary results. *Age (Dordr)* 2013; **35**(6): 2345-55.

79. Magalhães JE, Barros IML, Pedrosa RP, Sampaio Rocha-Filho PA. Migraine and Markers of Carotid Atherosclerosis in Middle-Aged Women: A Cross-Sectional Study. *Headache* 2019; **59**(1): 77-85.
80. Maimaitiaili R, Teliewubai J, Zhao S, et al. Relationship Between Vascular Aging and Left Ventricular Concentric Geometry in Community-Dwelling Elderly: The Northern Shanghai Study. *Clin Interv Aging* 2020; **15**: 853-63.
81. Maldonado J, Pereira T, Polónia J, Silva JA, Morais J, Marques M. Arterial stiffness predicts cardiovascular outcome in a low-to-moderate cardiovascular risk population: the EDIVA (Estudo de DIstensibilidade VAscular) project. *J Hypertens* 2011; **29**(4): 669-75.
82. Matsumoto M, Suganuma H, Shimizu S, et al. Skin Carotenoid Level as an Alternative Marker of Serum Total Carotenoid Concentration and Vegetable Intake Correlates with Biomarkers of Circulatory Diseases and Metabolic Syndrome. *Nutrients* 2020; **12**(6).
83. Matsuoka O, Otsuka K, Murakami S, et al. Arterial stiffness independently predicts cardiovascular events in an elderly community -- Longitudinal Investigation for the Longevity and Aging in Hokkaido County (LILAC) study. *Biomed Pharmacother* 2005; **59 Suppl 1**(Suppl 1): S40-4.
84. McEniery CM, Spratt M, Munnery M, et al. An analysis of prospective risk factors for aortic stiffness in men: 20-year follow-up from the Caerphilly prospective study. *Hypertension* 2010; **56**(1): 36-43.
85. Metsämarttila E, Rodilla E, Jokelainen J, et al. Effect of physical activity on pulse wave velocity in elderly subjects with normal glucose, prediabetes or Type 2 Diabetes. *Sci Rep* 2018; **8**(1): 8045.
86. Michener KH, Mitchell GF, Noubary F, et al. Aortic stiffness and kidney disease in an elderly population. *Am J Nephrol* 2015; **41**(4-5): 320-8.
87. Mikumo M, Okano H, Yoshikata R, Ishitani K, Ohta H. Association between lumbar bone mineral density and vascular stiffness as assessed by pulse wave velocity in postmenopausal women. *J Bone Miner Metab* 2009; **27**(1): 89-94.
88. Miljkovic D, Perret-Guillaume C, Alla F, Salvi P, Erpelding ML, Benetos A. Correlation between peripheral blood pressure and pulse-wave velocity values in the institutionalized elderly persons 80 years of age and older: the PARTAGE study. *Am J Hypertens* 2013; **26**(2): 163-73.
89. Miyaki K, Hara A, Naito M, Naito T, Nakayama T. Two new criteria of the metabolic syndrome: prevalence and the association with brachial-ankle pulse wave velocity in Japanese male workers. *J Occup Health* 2006; **48**(2): 134-40.
90. Mokwatsi GG, Schutte AE, Kruger R. Ethnic differences regarding arterial stiffness of 6-8-year-old black and white boys. *J Hypertens* 2017; **35**(5): 960-7.
91. Muhammad IF, Borné Y, Östling G, et al. Acute phase proteins as prospective risk markers for arterial stiffness: The Malmö Diet and Cancer cohort. *PLoS One* 2017; **12**(7): e0181718.
92. Nakagomi A, Sunami Y, Kawasaki Y, Fujisawa T, Kobayashi Y. Sex difference in the association between surrogate markers of insulin resistance and arterial stiffness. *J Diabetes Complications* 2020; **34**(6): 107442.
93. Nakamura M, Sugiura M, Ogawa K, Ikoma Y, Yano M. Serum β-cryptoxanthin and β-carotene derived from Satsuma mandarin and brachial-ankle pulse wave velocity: The Mikkabi cohort study. *Nutr Metab Cardiovasc Dis* 2016; **26**(9): 808-14.
94. Nakanishi N, Shiraishi T, Wada M. Brachial-ankle pulse wave velocity and metabolic syndrome in a Japanese population: the Minoh study. *Hypertens Res* 2005; **28**(2): 125-31.
95. Niboshi A, Hamaoka K, Sakata K, Inoue F. Characteristics of brachial-ankle pulse wave velocity in Japanese children. *Eur J Pediatr* 2006; **165**(9): 625-9.
96. Ninomiya T, Kojima I, Doi Y, et al. Brachial-ankle pulse wave velocity predicts the development of cardiovascular disease in a general Japanese population: the Hisayama Study. *J Hypertens* 2013; **31**(3): 477-83; discussion 83.

97. Oikonomou E, Vogiatzi G, Lazaros G, et al. Relationship of depressive symptoms with arterial stiffness and carotid atherosclerotic burden in the Corinthia study. *Qjm* 2020; **113**(9): 633-42.
98. Oren A, Vos LE, Uiterwaal CS, Grobbee DE, Bots ML. Aortic stiffness and carotid intima-media thickness: two independent markers of subclinical vascular damage in young adults? *Eur J Clin Invest* 2003; **33**(11): 949-54.
99. Oughton JA, Rose S, Galloway G, Khoo SK, O'Neill S, Coulthard A. Carotid ultrasound pulsatility indices and cardiovascular risk in Australian women. *J Med Imaging Radiat Oncol* 2015; **59**(1): 20-5.
100. Paini A, Aggiusti C, Bertacchini F, et al. Relationship between arterial stiffness and unattended or attended blood pressure values. *J Hypertens* 2020; **38**(2): 243-8.
101. Pan J, Xu L, Lam TH, et al. Relationship between pulmonary function and peripheral vascular function in older Chinese: Guangzhou biobank cohort study-CVD. *BMC Pulm Med* 2018; **18**(1): 74.
102. Pan FF, Xu CC, Hu TJ, Fu GX, Zhong Y. Carotid plague formation is associated with ankle-brachial index in elderly people. *Aging Clin Exp Res* 2020; **32**(11): 2217-23.
103. Park JS, Kang S, Ahn CW, Cha BS, Kim KR, Lee HC. Relationships between serum uric acid, adiponectin and arterial stiffness in postmenopausal women. *Maturitas* 2012; **73**(4): 344-8.
104. Pivin E, Ponte B, Pruijm M, et al. Inactive Matrix Gla-Protein Is Associated With Arterial Stiffness in an Adult Population-Based Study. *Hypertension* 2015; **66**(1): 85-92.
105. Podolec M, Siniarski A, Pająk A, et al. Association between carotid-femoral pulse wave velocity and overall cardiovascular risk score assessed by the SCORE system in urban Polish population. *Kardiol Pol* 2019; **77**(3): 363-70.
106. Poon AK, Meyer ML, Tanaka H, et al. Association of insulin resistance, from mid-life to late-life, with aortic stiffness in late-life: the Atherosclerosis Risk in Communities Study. *Cardiovasc Diabetol* 2020; **19**(1): 11.
107. Recio-Rodriguez JI, Gomez-Marcos MA, Patino-Alonso MC, et al. Association between fat amount of dairy products with pulse wave velocity and carotid intima-media thickness in adults. *Nutr J* 2014; **13**: 37.
108. Reusz GS, Cseprekal O, Temmar M, et al. Reference values of pulse wave velocity in healthy children and teenagers. *Hypertension* 2010; **56**(2): 217-24.
109. Rhee TM, Kim HL, Oh S, et al. Gender difference in the association between brachial-ankle pulse wave velocity and cardiovascular risk scores. *Korean J Intern Med* 2019; **34**(3): 539-48.
110. Rojek M, Rajzer M, Wojciechowska W, Gąsowski J, Pizoń T, Czarnecka D. The relation between blood pressure components and left atrial volume in the context of left ventricular mass index. *Medicine (Baltimore)* 2017; **96**(52): e9459.
111. Saijo Y, Utsugi M, Yoshioka E, et al. Relationship of Helicobacter pylori infection to arterial stiffness in Japanese subjects. *Hypertens Res* 2005; **28**(4): 283-92.
112. Saji N, Kimura K, Shimizu H, Kita Y. Association between silent brain infarct and arterial stiffness indicated by brachial-ankle pulse wave velocity. *Intern Med* 2012; **51**(9): 1003-8.
113. Samargandy S, Matthews KA, Brooks MM, et al. Arterial Stiffness Accelerates Within 1 Year of the Final Menstrual Period: The SWAN Heart Study. *Arterioscler Thromb Vasc Biol* 2020; **40**(4): 1001-8.
114. Sang Y, Wu X, Miao J, Cao M, Ruan L, Zhang C. Determinants of Brachial-Ankle Pulse Wave Velocity and Vascular Aging in Healthy Older Subjects. *Med Sci Monit* 2020; **26**: e923112.
115. Scuteri A, Orru M, Morrell CH, et al. Associations of large artery structure and function with adiposity: effects of age, gender, and hypertension. The SardiNIA Study. *Atherosclerosis* 2012; **221**(1): 189-97.
116. Segers P, Rietzschel ER, De Buyzere ML, et al. Noninvasive (input) impedance, pulse wave velocity, and wave reflection in healthy middle-aged men and women. *Hypertension* 2007; **49**(6): 1248-55.

117. Sforza E, Millasseau S, Hupin D, Barthélémy JC, Roche F. Arterial stiffness alteration and obstructive sleep apnea in an elderly cohort free of cardiovascular event history: the PROOF cohort study. *Sleep Breath* 2019; **23**(1): 201-8.
118. Sheng CS, Li Y, Li LH, et al. Brachial-ankle pulse wave velocity as a predictor of mortality in elderly Chinese. *Hypertension* 2014; **64**(5): 1124-30.
119. Shokawa T, Imazu M, Yamamoto H, et al. Pulse wave velocity predicts cardiovascular mortality: findings from the Hawaii-Los Angeles-Hiroshima study. *Circ J* 2005; **69**(3): 259-64.
120. Silva AB, Capingana DP, Magalhães P, Molina Mdel C, Baldo MP, Mill JG. Predictors and Reference Values of Pulse Wave Velocity in Prepubertal Angolan Children. *J Clin Hypertens (Greenwich)* 2016; **18**(8): 725-32.
121. Sonoda H, Takase H, Dohi Y, Kimura G. Factors associated with brachial-ankle pulse wave velocity in the general population. *J Hum Hypertens* 2012; **26**(12): 701-5.
122. Sougawa Y, Miyai N, Utsumi M, Miyashita K, Takeda S, Arita M. Brachial-ankle pulse wave velocity in healthy Japanese adolescents: reference values for the assessment of arterial stiffness and cardiovascular risk profiles. *Hypertens Res* 2020; **43**(4): 331-41.
123. Stamatelopoulos K, Tsoltos N, Armeni E, et al. Physical activity is associated with lower arterial stiffness in normal-weight postmenopausal women. *J Clin Hypertens (Greenwich)* 2020; **22**(9): 1682-90.
124. Strazhesko ID, Tkacheva ON, Akasheva DU, et al. Growth Hormone, Insulin-Like Growth Factor-1, Insulin Resistance, and Leukocyte Telomere Length as Determinants of Arterial Aging in Subjects Free of Cardiovascular Diseases. *Front Genet* 2017; **8**: 198.
125. Su HM, Lin TH, Hsu PC, et al. Association of interankle systolic blood pressure difference with peripheral vascular disease and left ventricular mass index. *Am J Hypertens* 2014; **27**(1): 32-7.
126. Takahashi T, Tomiyama H, Aboyans V, et al. Association of pulse wave velocity and pressure wave reflection with the ankle-brachial pressure index in Japanese men not suffering from peripheral artery disease. *Atherosclerosis* 2021; **317**: 29-35.
127. Takashima N, Turin TC, Matsui K, et al. The relationship of brachial-ankle pulse wave velocity to future cardiovascular disease events in the general Japanese population: the Takashima Study. *J Hum Hypertens* 2014; **28**(5): 323-7.
128. Tang B, Luo F, Zhao J, et al. Relationship between body mass index and arterial stiffness in a health assessment Chinese population. *Medicine (Baltimore)* 2020; **99**(3): e18793.
129. Temmar M, Watfa G, Joly L, et al. Elderly Algerian women lose their sex-advantage in terms of arterial stiffness and cardiovascular profile. *J Hypertens* 2013; **31**(11): 2244-50; discussion 50.
130. Tomiyama H, Yamashina A, Arai T, et al. Influences of age and gender on results of noninvasive brachial-ankle pulse wave velocity measurement--a survey of 12517 subjects. *Atherosclerosis* 2003; **166**(2): 303-9.
131. Topel ML, Shen J, Morris AA, et al. Comparisons of the Framingham and Pooled Cohort Equation Risk Scores for Detecting Subclinical Vascular Disease in Blacks Versus Whites. *Am J Cardiol* 2018; **121**(5): 564-9.
132. Torigoe T, Dallaire F, Slorach C, et al. New Comprehensive Reference Values for Arterial Vascular Parameters in Children. *J Am Soc Echocardiogr* 2020; **33**(8): 1014-22.e4.
133. Tsao CW, Washington F, Musani SK, et al. Clinical Correlates of Aortic Stiffness and Wave Amplitude in Black Men and Women in the Community. *J Am Heart Assoc* 2018; **7**(21): e008431.
134. Tsuchikura S, Shoji T, Kimoto E, et al. Brachial-ankle pulse wave velocity as an index of central arterial stiffness. *J Atheroscler Thromb* 2010; **17**(6): 658-65.
135. Uetani E, Tabara Y, Igase M, et al. Postprandial hypertension, an overlooked risk marker for arteriosclerosis. *Atherosclerosis* 2012; **224**(2): 500-5.
136. Urbina EM, Gao Z, Khoury PR, Martin LJ, Dolan LM. Insulin resistance and arterial stiffness in healthy adolescents and young adults. *Diabetologia* 2012; **55**(3): 625-31.

137. van den Munckhof ICL, Holewijn S, de Graaf J, Rutten JHW. Sex differences in fat distribution influence the association between BMI and arterial stiffness. *J Hypertens* 2017; **35**(6): 1219-25.
138. van Varik BJ, Vossen LM, Rennenberg RJ, et al. Arterial stiffness and decline of renal function in a primary care population. *Hypertens Res* 2017; **40**(1): 73-8.
139. Waldstein SR, Rice SC, Thayer JF, Najjar SS, Scuteri A, Zonderman AB. Pulse pressure and pulse wave velocity are related to cognitive decline in the Baltimore Longitudinal Study of Aging. *Hypertension* 2008; **51**(1): 99-104.
140. Wang KL, Cheng HM, Chuang SY, et al. Central or peripheral systolic or pulse pressure: which best relates to target organs and future mortality? *J Hypertens* 2009; **27**(3): 461-7.
141. Wang L, Niu JY, Zhao ZY, et al. Ideal Cardiovascular Health is Inversely Associated with Subclinical Atherosclerosis: A Prospective Analysis. *Biomed Environ Sci* 2019; **32**(4): 260-71.
142. Watanabe Y, Masaki H, Yunoki Y, et al. Ankle-Brachial Index, Toe-Brachial Index, and Pulse Volume Recording in Healthy Young Adults. *Ann Vasc Dis* 2015; **8**(3): 227-35.
143. Wendell CR, Waldstein SR, Evans MK, Zonderman AB. Distributions of Subclinical Cardiovascular Disease in a Socioeconomically and Racially Diverse Sample. *Stroke* 2017; **48**(4): 850-6.
144. Wijnands JM, Boonen A, van Sloten TT, et al. Association between serum uric acid, aortic, carotid and femoral stiffness among adults aged 40-75 years without and with type 2 diabetes mellitus: The Maastricht Study. *J Hypertens* 2015; **33**(8): 1642-50.
145. Wohlfahrt P, Somers VK, Cifkova R, et al. Relationship between measures of central and general adiposity with aortic stiffness in the general population. *Atherosclerosis* 2014; **235**(2): 625-31.
146. Woodiwiss AJ, Norton GR, Ben-Dov IZ, Gavish B, Bursztyn M. Association of Blood Pressure Variability Ratio With Glomerular Filtration Rate Independent of Blood Pressure and Pulse Wave Velocity. *Am J Hypertens* 2017; **30**(12): 1177-88.
147. Wu Y, Zhang Y, Gao J, et al. Effect of brachial-ankle pulse wave velocity combined with blood pressure on cardio-cerebrovascular events. *Exp Ther Med* 2019; **18**(6): 4555-66.
148. Xie X, Ma YT, Yang YN, et al. Decreased estimated glomerular filtration rate (eGFR) is not an independent risk factor of arterial stiffness in Chinese women. *Blood Press* 2013; **22**(2): 73-9.
149. Xuan Y, Wang W, Zhang H, et al. Osteoporosis is inversely associated with arterial stiffness in the elderly: An investigation using the Osteoporosis Self-assessment Tool for Asians index in an elderly Chinese cohort. *J Clin Hypertens (Greenwich)* 2019; **21**(3): 405-11.
150. Yang F, Wang G, Wang Z, et al. Visceral adiposity index may be a surrogate marker for the assessment of the effects of obesity on arterial stiffness. *PLoS One* 2014; **9**(8): e104365.
151. Zachariah JP, Rong J, Larson MG, et al. Metabolic Predictors of Change in Vascular Function: Prospective Associations From a Community-Based Cohort. *Hypertension* 2018; **71**(2): 237-42.
152. Mitchell GF, Wang N, Palmisano JN, et al. Hemodynamic correlates of blood pressure across the adult age spectrum: noninvasive evaluation in the Framingham Heart Study. *Circulation* 2010; **122**(14): 1379-86.
153. Zaniqueli D, Alvim RO, Luiz SG, Oliosa PR, de Sá Cunha R, Mill JG. Ethnicity and arterial stiffness in children and adolescents from a Brazilian population. *J Hypertens* 2017; **35**(11): 2257-61.
154. Zeng Q, Dong SY, Wang ML, et al. Serum glycated albumin, glycated hemoglobin, and arterial stiffness in a general Chinese population. *Clin Chim Acta* 2017; **468**: 33-8.
155. Zhang J, Fang L, Qiu L, Huang L, Zhu W, Yu Y. Comparison of the ability to identify arterial stiffness between two new anthropometric indices and classical obesity indices in Chinese adults. *Atherosclerosis* 2017; **263**: 263-71.
156. Zhang Y, Miyai N, Abe K, et al. Muscle mass reduction, low muscle strength, and their combination are associated with arterial stiffness in community-dwelling elderly population: the Wakayama Study. *J Hum Hypertens* 2021; **35**(5): 446-54.

157. Zhao W, Gong W, Wu N, et al. Association of lipid profiles and the ratios with arterial stiffness in middle-aged and elderly Chinese. *Lipids Health Dis* 2014; **13**: 37.
158. Zheng Y, Li Z, Shu H, Liu M, Chen Z, Huang J. Relationship between sum of the four limbs' pulse pressure and brachial-ankle pulse wave velocity and atherosclerosis risk factors in Chinese adults. *Biomed Res Int* 2015; **2015**: 434516.
159. Zhou Y, Yang R, Li C, Tao M. Sleep disorder, an independent risk associated with arterial stiffness in menopause. *Sci Rep* 2017; **7**(1): 1904.
160. Zureik M, Temmar M, Adamopoulos C, et al. Carotid plaques, but not common carotid intima-media thickness, are independently associated with aortic stiffness. *J Hypertens* 2002; **20**(1): 85-93.
161. Zureik M, Czernichow S, Courbon D, et al. Parental longevity, carotid atherosclerosis, and aortic arterial stiffness in adult offspring. *Stroke* 2006; **37**(11): 2702-7.
