# Supplementary material for: Global distributions of age- and sex-related arterial stiffness: systematic review and meta-analysis of 167 studies with 509,743 participants
Source: eBioMedicine. 2023 May 23;92:104619. doi: 10.1016/j.ebiom.2023.104619 (PMC10327869; doi:10.1016/j.ebiom.2023.104619)
Supplement: Protocol for a systematic review and meta analysis [file mmc2.docx]

**Protocol for a** **systematic review and meta analysis of global distributions of age- and sex-related arterial stiffness**

Yao Lu^*^, Sophia J. Kiechl^*^, Jie Wang, Qingbo Xu, Stefan Kiechl, and Raimund Pechlaner

**Clinical Research Center, The Third Xiangya Hospital, Central South University, Changsha, China** (Prof Y Lu, J Wang MD); **School of Life Course Sciences, King’s College London, London, United Kingdom** (Prof Y Lu); **Department of Neurology, Medical University of Innsbruck, Innsbruck, Austria** (S J Kiechl PhD, Prof S Kiechl, R Pechlaner PhD); **Department of Neurology, Hochzirl Hospital, Zirl, Austria** (S J Kiechl PhD); **Research Centre on Vascular Ageing and Stroke, Innsbruck, Austria** (S J Kiechl PhD, Prof S Kiechl); **Centre for Clinical Pharmacology, William Harvey Research Institute, Barts and The London School of Medicine and Dentistry, Queen Mary University of London, United Kingdom** (Prof Q Xu)

* These authors contributed equally.

Correspondence to:

Stefan Kiechl, Department of Neurology, Medical University of Innsbruck, Austria
**Stefan.kiechl@i-med.ac.at**

Yao Lu, Clinical Research Center, The Third Xiangya Hospital, Central South University, Changsha, China *and* Department of Life Science and Medicine, King’s College London, London, United Kingdom;
**yao.lu@kcl.ac.uk**

Raimund Pechlaner, Department of Neurology, Medical University of Innsbruck, Austria
**raimund.pechlaner@i-med.ac.at**

**Author Contributions:** YL, JW and RP drafted the protocol. SJK reviewed the protocol. All authors have read and approved the protocol.

**Guarantors:** SK and YL

**Funding:**

This meta-analysis will be supported by the excellence initiative VASCage (Research Centre on Vascular Ageing and Stroke, project number 868624) of the Austrian Research Promotion Agency FFG (COMET program–Competence Centers for Excellent Technologies) funded by the Federal Ministry for Climate Protection, Environment, Energy, Transport, Innovation and Technology; the Federal Ministry for Labour and Economy; and the federal states Tyrol (via Standortagentur), Salzburg, and Vienna (via Vienna Business Agency); as well as by the National Science Foundation of China (81800393, 81870171, and 81570271) and the Science and Technology Planning Project of Hunan Province (2019RS2014).

The funders hat no role in preparation of this protocol and will have no role in the analysis itself or the decision to submit for publication.

**Synopsis**

**Background**

Arterial stiffening is a powerful predictor and cause of diverse vascular pathologies and mortality. However, its clinical and research use are limited by a lack of established reference values. Thus, we aim at creating a global compendium of arterial stiffness distributions and reference values.

**Methods**

A systematic review and meta-analysis will be conducted. The electronic databases MEDLINE, Web of Science, and EMBASE will be searched without language restrictions using predefined search terms to identify eligible studies that report measurements of carotid-femoral or brachial-ankle pulse wave velocity (PWV; cfPWV or baPWV) in generally healthy humans. Both individual participant and summary data will be considered. Reviews, case reports, patient series, experimental studies, or very small studies will not be included. Methodological quality of included studies will be graded using the Joanna Briggs Instrument. Standardization of PWV by age and sex, identification of regional differences in PWV, and construction of reference percentiles for PWV will be performed by mixed-effects meta-regression.

**Discussion**

The review and meta-analysis will be the first to provide global age- and sex-dependent differences in and reference distributions for PWV in generally healthy individuals, which may facilitate use of PWV as a marker of vascular ageing, for prediction of vascular risk and death, and for designing future therapeutic interventions.

**Introduction**

Cardiovascular disease (CVD) is the leading cause of mortality globally and a major contributor to reduced quality of life.^1^ Estimation of the epidemiological burden of arterial stiffness can serve as a basis for prevention and management of CVD. Arterial stiffness is a strong predictor and cause of CVD, and may be particularly useful for detection of subclinical vascular disease. Pulse wave velocity (PWV) is a validated, non-invasive measure of arterial stiffness^2,3^ that represents the speed of the arterial pressure wave propagation along an artery and increases in parallel with stiffness of the vessel wall. Aortic PWV is a powerful predictor of CVD independent of traditional risk factors^4,5^ and of all-cause mortality with a 15% increase for each one m/s increase in PWV.^6^

Defining global distributions of PWV is a prerequisite for the development of effective strategies for primary prevention and improved management of CVD. Although measurement of PWV is straightforward,^7^ its interpretation is made difficult by a lack of established reference values, impeding identification of subjects with early, subclinical vascular disease. Prior meta-analyses that have attempted the creation of PWV reference values included only small fractions of all available PWV measurements and were restricted to carotid-femoral PWV (cfPWV) and to Caucasian or European populations.^8,9^ Here, we plan a global meta-analysis including all available data on PWV measurements in generally healthy individuals to define global differences and reference distributions of PWV, including also brachial-ankle PWV (baPWV) measurements and non-Caucasian populations, and focusing on regional differences in PWV.

**Methods**

This protocol has been created in accordance with the Meta-analyses Of Observational Studies in Epidemiology (MOOSE) checklist and the Preferred Reporting Items for Systematic Review and Meta-Analysis Protocols (PRISMA-P) guidelines.^10,11^ The completed PRISMA-P checklist is available in Table S1. The aim of this systematic review and meta analysis will be to create age- and sex-dependent distributions and reference values for baPWV and cfPWV based on PWV measurements in generally healthy individuals without regional restrictions. This meta-analysis will be the first large-scale analysis created by the *Global Pulse Wave Velocity Study Group*.

**Data sources, search terms, and search strategy**

The systematic review will identify studies by searching the electronic databases MEDLINE, Web of Science, and EMBASE without language restrictions. The search strategy is shown in Table 1. The search strategy consists of possible combinations of terms identifying measurements of cfPWV/baPWV. The search will be adjusted according to the requirements of each specific database (i.e., use of operators and symbols) as detailed in Table S2. The risks of selection and detection bias are considered moderate for this meta-analysis because no associations with clinical outcomes are to be considered and the focus lies solely on aggregating measured PWV values.

**Inclusion and exclusion criteria**

Studies will be included if they provide measurements, at the individual or sample level, of cfPWV or baPWV in generally healthy humans. Study or study group participants will be considered generally healthy if they can reasonably be seen as representative of the unselected general community. Thus, medical conditions may be present in the same prevalence as in the general community source population of each study sample, but individuals and groups defined by the presence of medical conditions, by intake of medications, or by receiving experimental or other medical interventions will not be eligible. Overall, most eligible studies are expected to derive from community-based observational studies. Reviews, case reports, repeated studies, patient series, animal studies, or very small studies (sample size less than 100) will not be included. If multiple articles be found that provide appropriate data from the same survey, we will include that with the largest sample size.

Studies that do not report PWV in continuous form will not be included, but all continuous PWV measurements, including mean, standard deviation (SD), median, interquartile range, and other quantile measures will be considered.

All researchers of studies identified by the systematic literature search will be contacted and invited to join the Global Pulse Wave Velocity Study Group, and asked to provide anonymized individual participant data or, if this is not possible, summary statistics for PWV in the defined format of mean and SD in decades of age, by sex. Further studies or unpublished data suggested by Global PWV Study Group members and studies cited by included studies will also be considered.

No language restrictions will be applied to the literature search. For studies that are not in English, Chinese, German, French, Italian, or Spanish, machine translation will be used to judge eligibility. No restrictions by publication year will be applied.

Only studies using the common PWV measurement methods tonometry, oscillometry, or ultrasound will be considered, and studies measuring PWV by magnetic resonance or studies employing pulse contour analysis will be excluded.

References identified will be imported into reference manager software (EndNote X9, Thomson Research Soft, US). In a first pass, duplicate references will be deleted using the software. In a second pass, two researchers will independently review the titles and abstracts of remaining articles and exclude articles for which such exclusion is possible based on the information in title and abstract. Finally, two researchers will independently evaluate the full text of remaining references. If the two researchers disagree on whether an article should be included, disagreement will be resolved by consulting a third, senior researcher.

**Participants and Data extraction**

The meta-analysis is planned to have a broad focus and include all available PWV measurements in generally healthy humans, with generally healthy understood as representative of the general community. Applying this definition will include subjects with medical conditions in prevalence and severities as in the general community of each included country, but exclude individuals selected by medical conditions or interventions. No restrictions will be placed on participants’ gender, ethnicity, or other demographic characteristics.

Data will be extracted by two independent researchers using a pre-defined data extraction and coding template. The following data will be extracted and documented: PWV or PWV summary statistics, PWV type, data source (individual-participant data provided by study authors, summary data provided by study authors, or summary data extracted from article), age and sex of participants or for study groups, sample sizes, study country, device used to measure PWV, path length method used (if and how landmarks on the body surface were defined for the calculation of PWV), first author name, and year of survey as well as publication year.

Individual-participant and summary data will be allocated to groups defined by decade of age, sex, study, and study country. Participants aged 90 years or older will be included in a ≥90 age group. If results of the two independent reviewers’ extractions disagree, a third, senior researcher will arbitrate to facilitate consensus.

For extracted data that are reported PWV as quantile measures, single regression imputation based on models fitted in individual participant data will be used to transform the available information on PWV measurements to means and SDs utilizing all information on the distributions of PWV available. For extracted data that are not reported in decades of age, data will be pooled or divided into decades of age by weighted averaging. The researchers are aware of a large number of studies reporting PWV values such that there is availability of a sufficient minimum number of studies.

**Assessment of methodological quality and risk of bias**

The Joanna Briggs Instrument (JBI) for Analytical Cross Sectional Studies will be used to assess the quality of included studies. The individual items of this checklist are shown in Table 1. Item 4: “Were objective, standard criteria used for measurement of the condition?” will not be used because data from individuals selected by presence of defined medical conditions will not be included. Two independent researchers will independently assess the quality of all studies, and disagreement will be resolved by consulting a third, senior researcher.

For unpublished datasets that may also be included, the JBI will not be applicable as it grades information reported in manuscripts. In this case, equivalent quality assurance will be performed by direct correspondence with the researchers providing the unpublished data.

**Statistical analysis plan**

Two main lines of analysis are planned: A comparison of average PWV between different world regions, countries and other groups, and the construction of country-specific PWV reference values.

Differences in average PWV will be investigated using inverse variance weighted linear mixed-effects meta-regression utilizing averages of PWV by sex and decade of age as predictors. Nonlinear fixed age effects are anticipated^12^ and will be modelled using restricted cubic splines. Autocorrelation of PWV by age will be accounted for by random effects with an autoregressive AR(1) covariance structure for age in addition to random intercepts for individual studies. For this analysis, individual participant data will be averaged by study, decade of age, and sex, and pooled with summary data. In addition to differences by country and world region, differences in PWV will be investigated by country income, study quality, size, and year, and measurement method, device, and path length measure used in PWV measurement. Age standardization will be performed by weighted averaging of model predictions using the age distribution of the World Health Organization World 2000-2025 Standard Million population. Results will be reported as average PWV overall and in subgroups.

Reference values and distributions of PWV, overall and by country, will be created using Generalized Additive Models for Location, Scale and Shape (GAMLSS).^13^ The best-fitting distribution for PWV will be determined by minimizing Bayesian Information Criterion and model fit will be investigated using worm plots and visual examination of statistical moments conditional on the predictors. The full age- and sex-conditional distribution of PWV will be modeled using smooth spline fits of all statistical moments of the selected distribution, and study-wise variation accounted for by including random intercepts. Adequate sample sizes within subgroups and adequate variability in age will be determined based on the data gathered, although it is anticipated that meaningful reference curves will not be feasible with very low subject counts (<100) or low variation in age (<30 years). Results will be reported as 5^th^, 25^th^, 50^th^, 75^th^, and 95^th^ percentile of PWV overall and in subgroups.

Deviations from this analysis plan will be made explicit in the manuscript and appropriately justified.

**Discussion**

This systematic review and meta-analysis will systematically aggregate all available global and country-specific measurements of baPWV and cfPWV from generally healthy, community-dwelling individuals. It will thereby enable a comparison of PWV between countries and world regions that was hitherto unavailable, define determinants of PWV at the population level, and improve interpretation of measured PWV values by allowing incorporation of country-specific normal values. The anticipated findings will increase the usefulness of PWV in clinical and research applications such as prediction of vascular disease and death.

**Limitations**

The planned meta-analysis aims to include all available PWV measurements in generally healthy individuals, which will yield a compendium of global PWV distributions of unprecedented size and breadth. This broad scope implies that heterogeneous studies will be combined and that the analysis will be agnostic to the impact of risk factors or other environmental factors. Smaller meta-analyses with a narrower focus have attempted at providing reference values for PWV conditional on environmental covariables.^8^ Overall, heterogeneity of studies is expected to be smoothed by an expected large number of included studies.

Furthermore, preliminary literature searches indicate that the number of relevant studies performed for baPWV and cfPWV respectively, as well as different measurement methodologies are unevenly distributed among world regions and countries, such that for some countries no data may be available, and also such that the simultaneous impact of different determinants of PWV may not be identifiable.

Further potential limitations include that some studies may report insufficient demographic characteristics and their exclusion could introduce publication bias. We will mitigate this risk by contacting all researchers of such studies to request relevant additional information.

Table 1. JBI Critical appraisal checklist for analytical cross sectional studies. Item 4 will not be considered.

| Reviewer : | | | | |
| --- | --- | --- | --- | --- |
| Date: | | | | |
| Author: | | | | |
| Year: | | Record Number: | | |
|  | Yes | No | Unclear | Not applicable |
| 1. Were the criteria for inclusion in the sample clearly defined? | □ | □ | □ | □ |
| 2. Were the study subjects and the setting described in detail? | □ | □ | □ | □ |
| 3. Was the exposure measured in a valid and reliable way? | □ | □ | □ | □ |
| 4. Were objective, standard criteria used for measurement of the condition? | □ | □ | □ | □ |
| 5. Were confounding factors identified? | □ | □ | □ | □ |
| 6. Were strategies to deal with confounding factors stated? | □ | □ | □ | □ |
| 7. Were the outcomes measured in a valid and reliable way? | □ | □ | □ | □ |
| 8. Was appropriate statistical analysis used? | □ | □ | □ | □ |
| Overall appraisal: Include □ Exclude □ Seek further info □ | | | | |
| Comments (Including reason for exclusion) | | | | |

References

1 Roth GA, Mensah GA, Johnson CO, *et al.* Global Burden of Cardiovascular Diseases and Risk Factors, 1990–2019. *J Am Coll Cardiol.* 2020; **76**: 2982–3021.

2 Schellinger IN, Mattern K, Raaz U. The Hardest Part. *Arterioscler Thromb Vasc Biol.* 2019; **39**: 1301–6.

3 Munakata M. Brachial-Ankle Pulse Wave Velocity: Background, Method, and Clinical Evidence. *Pulse.* 2016; **3**: 195–204.

4 Ohkuma T, Ninomiya T, Tomiyama H, *et al.* Brachial-Ankle Pulse Wave Velocity and the Risk Prediction of Cardiovascular Disease. *Hypertension.* 2017; **69**: 1045–52.

5 Niiranen TJ, Kalesan B, Mitchell GF, Vasan RS. Relative Contributions of Pulse Pressure and Arterial Stiffness to Cardiovascular Disease. *Hypertension.* 2019; **73**: 712–7.

6 Vlachopoulos C, Aznaouridis K, Stefanadis C. Prediction of Cardiovascular Events and All-Cause Mortality With Arterial Stiffness: A Systematic Review and Meta-Analysis. *J Am Coll Cardiol.* 2010; **55**: 1318–27.

7 Tanaka H, Munakata M, Kawano Y, *et al.* Comparison between carotid-femoral and brachial-ankle pulse wave velocity as measures of arterial stiffness. *J Hypertens.* 2009; **27**: 2022–7.

8 The Reference Values for Arterial Stiffness’ Collaboration. Determinants of pulse wave velocity in healthy people and in the presence of cardiovascular risk factors: ‘establishing normal and reference values’. *Eur Heart J.* 2010; **31**: 2338–50.

9 Khoshdel AR, Thakkinstian A, Carney SL, Attia J. Estimation of an age-specific reference interval for pulse wave velocity: a meta-analysis. *J Hypertens.* 2006; **24**: 1231–7.

10 Stroup DF, Berlin JA, Morton SC, *et al.* Meta-analysis of observational studies in epidemiology: a proposal for reporting. Meta-analysis Of Observational Studies in Epidemiology (MOOSE) group. *JAMA.* 2000; **283**: 2008–12.

11 Moher D, Shamseer L, Clarke M, *et al.* Preferred reporting items for systematic review and meta-analysis protocols (PRISMA-P) 2015 statement. *Syst Rev.* 2015; **4**: 1.

12 Lu Y, Pechlaner R, Cai J, *et al.* Trajectories of Age-Related Arterial Stiffness in Chinese Men and Women. *J Am Coll Cardiol.* 2020; **75**: 870–80.

13 Rigby RA, Stasinopoulos DM. Generalized additive models for location, scale and shape. *Appl Stat.* 2005; **54**: 507–54.
