# Supplementary material for: Global distributions of age- and sex-related arterial stiffness: systematic review and meta-analysis of 167 studies with 509,743 participants
Source: eBioMedicine. 2023 May 23;92:104619. doi: 10.1016/j.ebiom.2023.104619 (PMC10327869; doi:10.1016/j.ebiom.2023.104619)
Supplement: Supplementary Appendix to Protocol [file mmc3.docx]

**Table S1 PRISMA-P Checklist.**

| **Section/topic** | **#** | **Checklist item** | **Information reported** | | **Line number(s)** |
| --- | --- | --- | --- | --- | --- |
|  |  |  | **Yes** | **No** |  |
| **ADMINISTRATIVE INFORMATION** | | | | | |
| **Title** | | | | | |
| Identification | 1a | Identify the report as a protocol of a systematic review | x |  | 1 |
| Update | 1b | If the protocol is for an update of a previous systematic review, identify as such |  | x |  |
| **Registration** | 2 | If registered, provide the name of the registry (e.g., PROSPERO) and registration number in the Abstract |  | x |  |
|  | | | | | |
| Contact | 3a | Provide name, institutional affiliation, and e-mail address of all protocol authors; provide physical mailing address of corresponding author | x |  | 6 |
| Contributions | 3b | Describe contributions of protocol authors and identify the guarantor of the review | x |  | 29 |
| **Amendments** | 4 | If the protocol represents an amendment of a previously completed or published protocol, identify as such and list changes; otherwise, state plan for documenting important protocol amendments |  | x |  |
|  | | | | | |
| Sources | 5a | Indicate sources of financial or other support for the review | x |  | 31 |
| Sponsor | 5b | Provide name for the review funder and/or sponsor |  | x |  |
| Role of sponsor/funder | 5c | Describe roles of funder(s), sponsor(s), and/or institution(s), if any, in developing the protocol | x |  | 41 |
|  | | | | | |
| **Rationale** | 6 | Describe the rationale for the review in the context of what is already known | x |  | 67 |
| **Objectives** | 7 | Provide an explicit statement of the question(s) the review will address with reference to participants, interventions, comparators, and outcomes (PICO) | x |  | 85 |
| **METHODS** | | | | | |
| **Eligibility criteria** | 8 | Specify the study characteristics (e.g., PICO, study design, setting, time frame) and report characteristics (e.g., years considered, language, publication status) to be used as criteria for eligibility for the review | x |  | 111-139 |
| **Information sources** | 9 | Describe all intended information sources (e.g., electronic databases, contact with study authors, trial registers, or other grey literature sources) with planned dates of coverage | x |  | 102 |
| **Search strategy** | 10 | Present draft of search strategy to be used for at least one electronic database, including planned limits, such that it could be repeated | x |  | 106 |
| ***STUDY RECORDS*** | | | | | |
| Data management | 11a | Describe the mechanism(s) that will be used to manage records and data throughout the review | x |  | 135 |
| Selection process | 11b | State the process that will be used for selecting studies (e.g., two independent reviewers) through each phase of the review (i.e., screening, eligibility, and inclusion in meta-analysis) | x |  | 139 |
| Data collection process | 11c | Describe planned method of extracting data from reports (e.g., piloting forms, done independently, in duplicate), any processes for obtaining and confirming data from investigators | x |  | 155 |
| **Data items** | 12 | List and define all variables for which data will be sought (e.g., PICO items, funding sources), any pre-planned data assumptions and simplifications | x |  | 157 |
| **Outcomes and prioritization** | 13 | List and define all outcomes for which data will be sought, including prioritization of main and additional outcomes, with rationale | x |  | 145, 112, 104 |
| **Risk of bias in individual studies** | 14 | Describe anticipated methods for assessing risk of bias of individual studies, including whether this will be done at the outcome or study level, or both; state how this information will be used in data synthesis | x |  | 176 |
| ***DATA*** | | | | | |
| **Synthesis** | 15a | Describe criteria under which study data will be quantitatively synthesized | x |  | 193-216 |
|  | 15b | If data are appropriate for quantitative synthesis, describe planned summary measures, methods of handling data, and methods of combining data from studies, including any planned exploration of consistency (e.g., *I* ^2^, Kendall’s tau) | x |  | 205, 216 |
|  | 15c | Describe any proposed additional analyses (e.g., sensitivity or subgroup analyses, meta-regression) | x |  | 199-202 |
|  | 15d | If quantitative synthesis is not appropriate, describe the type of summary planned |  | x |  |
| **Meta-bias(es)** | 16 | Specify any planned assessment of meta-bias(es) (e.g., publication bias across studies, selective reporting within studies) |  | x |  |
| **Confidence in cumulative evidence** | 17 | Describe how the strength of the body of evidence will be assessed (e.g., GRADE) | x |  | 57 |

**Table S2. Literature search strategy.**

| **Database** | **Search terms** |
| --- | --- |
| **PubMed** | (((arterial stiffness[Title/Abstract]) OR (arterial stiffening[Title/Abstract]) OR (artery stiffness[Title/Abstract]) OR (artery stiffening[Title/Abstract]) OR (vascular stiffness[Title/Abstract]) OR (vascular stiffening[Title/Abstract]) OR (aortic stiffness[Title/Abstract]) OR (aortic stiffening[Title/Abstract])) AND ((epidemiology[Title/Abstract]) OR (prevalence[Title/Abstract]))) OR (((PWV[Title/Abstract]) OR (pulse wave velocity[Title/Abstract]) OR (baPWV[Title/Abstract]) OR (brachial-ankle pulse wave velocity[Title/Abstract]) OR (cfPWV[Title/Abstract]) OR (carotid- femoral pulse wave velocity[Title/Abstract])) AND ((value[Title/Abstract]) OR (values[Title/Abstract]) OR (estimation[Title/Abstract]) OR (estimated[Title/Abstract]) OR (distribution[Title/Abstract]) OR (determinant[Title/Abstract]) OR (determinants[Title/Abstract]) OR (determination [Title/Abstract]) OR (assessment[Title/Abstract]))) |
| **MEDLINE** | "1 TI=(arterial stiffness OR arterial stiffening OR artery stiffness OR artery stiffening OR vascular stiffness OR vascular stiffening OR aortic stiffness OR aortic stiffening ) OR AB=(arterial stiffness OR arterial stiffening OR artery stiffness OR artery stiffening OR vascular stiffness OR vascular stiffening OR aortic stiffness OR aortic stiffening )  2 TI=(prevalence OR epidemiology) OR AB=(prevalence OR epidemiology)  3 1 AND 2  4 TI=(PWV OR pulse wave velocity OR baPWV OR brachial-ankle pulse wave velocity OR cfPWV OR carotid-femoral pulse wave velocity)OR AB=(PWV OR pulse wave velocity OR baPWV OR brachial-ankle pulse wave velocity OR cfPWV OR carotid-femoral pulse wave velocity)  5 TI=(value OR values OR estimation OR estimated OR distribution OR determinant OR determinants OR determination OR assessment)OR AB=(value OR values OR estimation OR estimated OR distribution OR determinant OR determinants OR determination OR assessment)  6 4 AND 5  7 3 OR 6  8 (#3 OR #6)AND 物种:(Humans) AND 状态:(MEDLINE) " |
| **EMBASE** | 1 (arterial-stiffness OR arterial-stiffening OR artery-stiffness OR artery-stiffening OR vascular-stiffness OR vascular-stiffening OR aortic-stiffness OR aortic-stiffening ):ab,ti  2 (prevalence OR epidemiology) :ab,ti  3 #1 AND #2  4 (PWV OR pulse-wave-velocity OR baPWV OR brachial-ankle-pulse-wave-velocity OR cfPWV OR carotid-femoral-pulse-wave-velocity):ab,ti  5 (value OR values OR estimation OR estimated OR distribution OR determinant OR determinants OR determination OR assessment):ab,ti  6 #4 and #5  7 #3 OR #6  8 (#3 OR #6) AND ([article]/lim OR [article in press]/lim OR [conference paper]/lim OR [short survey]/lim) AND [humans]/lim AND [embase]/lim |
